# Combination of Chinese herbal medicine and conventional western medicine for coronavirus disease 2019: a systematic review and meta-analysis

**DOI:** 10.3389/fmed.2023.1175827

**Published:** 2023-07-17

**Authors:** Lei Tong, Zhenyu Ma, Yixiao Zhou, Shuping Yang, Yalin Yang, Jingran Luo, Junbo Huang, Fucai Wang

**Affiliations:** School of Medicine, Huaqiao University, Quanzhou, China

**Keywords:** Chinese herbal medicine, COVID-19, systematic review, meta-analysis, randomized controlled trials

## Abstract

**Objective:**

This study aimed to assess the efficacy and safety of Chinese herbal medicine (CHM) plus conventional western medicine (CWM) in comparison with CWM against COVID-19.

**Methods:**

We searched eight electronic databases and three trial registers spanning from January 1, 2020 to May 18, 2023. We included randomized controlled trials (RCTs) comparing the effectiveness and safety of CHM plus CWM and CWM against COVID-19 in our study. The Cochrane Risk of Bias tool 2.0 (RoB2) was applied to evaluate the methodological quality of the included RCTs. The Grading of Recommendations, Assessment, Development, and Evaluation (GRADE) system was employed to assess the certainty of evidence. Statistical analysis was implemented in R version 4.1.2.

**Results:**

Our study included 50 RCTs involving 11,624 patients. In comparison with sole CWM, CHM plus CWM against COVID-19 significantly enhanced clinical effective rate (RR = 1.18, 95% CI [1.13, 1.22]), improved chest image (RR = 1.19, 95% CI [1.11, 1.28]), inhibited clinical deterioration (RR = 0.45, 95% CI [0.33, 0.60]), lowered mortality (RR = 0.53, 95% CI [0.40, 0.70]), and reduced the total score of TCM syndrome (SMD = −1.24, 95% CI [−1.82, −0.66]). SARS-CoV-2 nucleic acid conversion time (MD = −2.66, 95% CI [−3.88, −1.44]), duration of hospitalization (MD = −2.36, 95% CI [−3.89, −0.82]), and clinical symptom (fever, cough, fatigue, and shortness of breath) recovery times were shorter in CHM plus CWM groups than in CWM groups. Further, CHM plus CWM treatment was more conducive for some laboratory indicators returning to normal levels. No statistical difference was found in the incidence of total adverse reactions between the two groups (RR = 0.97, 95% CI [0.88, 1.07]). We assessed the risk of bias for 246 outcomes, and categorized 55 into “low risk”, 151 into “some concerns”, and 40 into “high risk”. Overall, the certainty of the evidence ranged from moderate to very low.

**Conclusions:**

Potentially, CHM listed in this study, as an adjunctive therapy, combining with CWM is an effective and safe therapy mode for COVID-19. However, more high-quality RCTs are needed to draw more accurate conclusions.

**Clinical trial registration:**

https://www.crd.york.ac.uk/PROSPERO/display_record.php?RecordID=293963.

## Introduction

The coronavirus disease 2019 (COVID-19) is a respiratory infectious disease that poses a severe threat to human health, caused by a novel coronavirus (SARS-CoV-2) (1). Since December 2019, when a large number of COVID-19 cases were detected in Wuhan, China, COVID-19 has evolved and spread rapidly worldwide (2). On March 11, 2020, due to the rapid transmission of the virus and the continued increase in confirmed cases, the World Health Organization (WHO) classified the current 2019 coronavirus disease outbreak as a global pandemic (3). At present, the global situation is still quite grim, and COVID-19 remains a threat to human health.

As the SARS-CoV-2 transmits from person to person, the virus is still in the process of evolution. Based on the impact of variants on transmission, disease severity, and capacity for immune escape, WHO has designated five variants as SARS-CoV-2 Variants of Concern (VOC). In addition, the “Omicron” variant, rampant across the world, is unlikely to be the final VOC (4, 5). The original SARS-CoV-2 and the new variants have posed enormous challenges and threats to global epidemic prevention and control. Scientific research has led to the development of vaccines (6), monoclonal antibodies, biologically active natural products (7–9), and small molecule formulations (10), to the extent that significant progress has been made in mitigating the threat of COVID-19. However, there is still no specific and effective drug to eliminate the virus, and conventional therapy for COVID-19 is mainly symptomatic and supportive treatment by Western medicine (11, 12). In addition, COVID-19 vaccines cannot stop the pandemic completely, as some people will still be infected after vaccination (13). From previous clinical experience, CHM is an option to combat various infectious diseases, such as SARS (14), influenza (15), and Ebola (16). During the early stage of COVID-19 outbreak in China, when the disease was not well-understood and no vaccine was available, Chinese doctors employed CHM to treat COVID-19 and achieved remarkable clinical effects (17). CHM was still widely applied in China for the treatment of COVID-19 patients. The combination of CHM and conventional western medicine (CWM) has been used in 92% of diagnosed COVID-19 cases, and more than 90% of patients received significant therapeutic effects (18). For patients with mild and moderate disease, early CHM treatment could be effective in preventing the progression to severe or critical cases. A lot of clinical practices have shown that early CHM intervention in patients with COVID-19 improved clinical cure rate, delayed disease progression, and lowered the risk of death (19). In the face of this epidemic, a range of Chinese herbal medicines have been recognized as very promising anti-SARS-CoV-2 agents, including active ingredients, monomer preparations, crude extracts, and formulas. All these agents have potential activity against SARS-CoV-2 and have attracted significant attention due to their activities both *in vitro* and in clinical practice (20). Therefore, CHM therapy has been included in the diagnosis and treatment guidelines for COVID-19 in China. Many CHMs, such as Qingfei Paidu Decoction (Granules), Jinhua Qinggan Granules, Lianhua Qingwen Capsules, and Xuanfei Baidu Granules, were proposed as adjunctive medicines for the treatment of COVID-19 (21).

Relevant systematic reviews concerning CHM efficacy assessment have been published, but with limitations (22–29). Some articles incorporated non-RCTs (e.g., observational studies), or pooled the data from non-RCTs and RCTs together (22, 23). Some articles only centered on a certain type of CHM, while others only focused on mild and moderate participants (18–20). Many articles didn't assess the certainty of evidence (23–25, 27, 28). Some failed to address the risk of bias for each outcome due to the inappropriate use of Cochrane Risk of Bias Tool 2.0 (RoB2) (22, 26, 29). A previous systematic review has only pooled the outcomes with low risk of bias, which could lead to omission of evidence (30). Additionally, some clinical trials employing new CHMs for the treatment of COVID-19 have been published, and the best evidence are in the process of constant change. Currently, no systematic review including data on patients infected with SRAS-CoV-2 Omicron variant has been published. Therefore, building upon published clinical studies, we conducted an assessment on the clinical indicators and the certainty of clinical evidence on the effectiveness and safety of CHM in the treatment of COVID-19 in this systematic review and meta-analysis. In the present study, we summarized the available evidence for CHM as an adjunctive treatment for COVID-19.

## Materials and methods

Our systematic review was reported in compliance the Preferred Reporting Items for Systematic Reviews and Meta-Analyses (PRISMA) 2020 statement (31). Our study was registered on International Prospective Register of Systematic Reviews (PROSPERO) with registration number CRD42021293963.

### Eligibility criteria

Inclusion criteria: (a) participants included confirmed COVID-19 patients (including asymptomatic patients), regardless of age, gender, ethnicity or severity of the disease; (b) participants in the intervention group received CHM plus CWM treatments, regardless of dosage, dosage forms, components, administration frequency and administration method; (c) participants in the control group received CWM treatments alone or CWM plus placebo treatments; (d) the study reported our outcome of interest; and (e) only randomized controlled trials (RCTs) were included in our systematic review.

Exclusion criteria: Studies including acupuncture, moxibustion, cupping therapy, massage, qigong therapy and music therapy as well as cohort studies, case-control studies, cross-sectional studies, case reports, clinical experiences, interviews, comments, letters, abstracts, and animal experiments were excluded.

### Search strategy

Researchers Z.M. and Y.Z. searched eight databases, including PubMed, Embase, the Cochrane Library, Web of Science, Chinese National Knowledge Infrastructure Database (CNKI), Chinese Science and Technology Journals Database (VIP), Chinese Biomedical Literature Database (CBM) and Wanfang Database. Chinese Clinical Trial Registration Center (ChiCTR), WHO International Clinical Trials Registry Platform (ICTRP), and ClinicalTrials.gov were also searched, ranging from January 1, 2020 to May 18, 2023, without any language and nationality limitations. Taking “PubMed” for example, literature search was conducted by means of a combination of MeSH terms and free-text terms. Specific search strategies were modified based on the characters of different databases and registers. All of the search strategies were listed in Supplementary Table S1.

### Study selection

Four reviewers (S.Y., Y.Y., Y.Z., and J.L.) were divided into two groups. All literature was divided into two parts and screened by two groups respectively. Then each of the researchers within the two groups made study selection by reading the title, abstract and full text independently in NoteExpress version 3.5. Any disagreements in results collation were settled by discussion or consulting to a third reviewer (J.H.).

### Data extraction

Data were extracted independently by two reviewers (SY and YY) from the included studies by means of a pre-designed data collection form. Any discrepancies between the two reviewers were settled by discussion or consulting to a third reviewer (FW). Moreover, all data collected were re-checked to ensure their accuracy. The extracted data included: (a) basic information (title, first author's name, and publication year); (b) study details (study design, original places of participants, sample size, severity condition of participants, age, and gender of participants); (c) interventions and controls (CHM, CWM, administration frequency, administration methods, dosage, components, and treatment duration); and (d) outcome measures and adverse reactions. If continuous data were reported as medians and interquartile ranges (IQRs), they would be converted into mean and SD values by mathematical methods, or obtained from other published meta-analyses (32, 33). In case of missing or incomplete information in outcome data, we contacted the corresponding authors via email. If no response was made, the data were re-calculated through the plots digitizer software; otherwise, the data would be excluded.

### Outcome details

We selected the outcomes in line with a core outcome set for COVID-19 based on traditional Chinese and Western medicine, as well as advice from clinicians (34). Due to the discrepancy between outcomes reported in the included original studies and our pre-determined outcomes, we adjusted and updated our registration on PROSPERO (on April 4, 2022). The primary outcomes were clinical effective rate and SARS-CoV-2 nucleic acid conversion time, while the secondary outcomes included chest image improvement, duration of hospitalization, condition of disease conversion, death, clinical symptoms recovery time (fever, cough, fatigue, and shortness of breath), total score of traditional Chinese medicine (TCM) syndrome, laboratory indicators, and adverse reactions. The conditions of disease were classified into mild, moderate, severe, or critical. The clinical classification was based on the protocol issued by the National Health Commission of the People's Republic of China, as follows, (1) mild cases: the clinical symptoms were mild and there were no signs of pneumonia on images; (2) moderate cases: COVID-19-related clinical manifestations such as fever and/or respiratory symptoms, and there were radiological findings of pneumonia; (3) severe cases: respiratory distress (respiratory rate ≥ 30 breaths/min), oxygen saturation ≤ 93% on air intake at rest, arterial partial pressure of oxygen (PaO_2_)/fraction of inspired oxygen (FiO_2_) ≤ 300 mmHg (1 mmHg = 0.133 kPa), or chest imaging showing significant lesion progression within 24 to 48 h > 50%; and (4) critical cases: respiratory failure requiring mechanical ventilation, shock, or other organ failures requiring intensive care unit (ICU) care (21).

### Risk of bias assessment

RoB2 was employed to assess the risk of bias of each outcome included in the meta-analysis in 5 domains: randomization process, deviation from intended intervention, missing outcome data, outcome measurement, and selection of the reported result (35). Two Signaling questions in the five domains were answered by two reviewers (ZM and YZ) separately, and the discrepancies were resolved by discussion or consulting to a third reviewer (LT). Each domain was categorized as “low risk”, “some concerns” or “high risk”. When all five domains were assessed as “low risk”, the overall bias of the outcome was considered as “low risk”.

### Data synthesis and statistical analysis

The R (version 4.1.2) was used to implement the statistical analysis (36). Considering the heterogeneity in different CHM interventions, we selected the random-effects model for the meta-analysis. The risk ratio (RR) with 95% confidence interval (CI) was evaluated for dichotomous outcomes (e.g., clinical effective rate). The mean difference (MD) with 95% CI was evaluated for continuous outcomes (e.g., SARS-CoV-2 nucleic acid conversion time). Whereas, due to the different rating scales, the total score of TCM syndrome was pooled by using the standardized mean difference (SMD). A significant difference was considered when *P* < 0.05. The *I*^2^ statistic was used to evaluate statistical heterogeneity between studies, and the values of 25, 50, and 75% signified the level of low, moderate, and high heterogeneity, respectively (37). Subgroup and sensitivity analysis were performed to search for sources of heterogeneity and test the robustness of the synthesized results. Egger's test and contour-enhanced funnel plots were accepted to evaluate the publication bias (38, 39). If a quantitative meta-analysis was not feasible, we conducted a qualitative analysis to show differences.

### Certainty of evidence assessment

The certainty of evidence was assessed through the Grading of Recommendations, Assessment, Development, and Evaluations (GRADE) system (40), which is downgraded for study limitations, inconsistency of results, indirectness of evidence, imprecision, and reporting bias. Optimal information size (OIS) is the number of patients required for an adequately powered trial. OIS was estimated assuming a type I error (α) of 0.05 and power of test (1 – β) of 0.8. If the number of observations did not satisfy the OIS principle, the certainty would potentially be downgraded due to imprecision. If the 95% CI overlaps the no-effect value (RR = 1.0), the certainty of evidence may be downgraded regardless of the OIS (41). The certainty of evidence was classified as high, moderate, low, and very low.

## Results

### Study search and selection result

**Figure 1:**
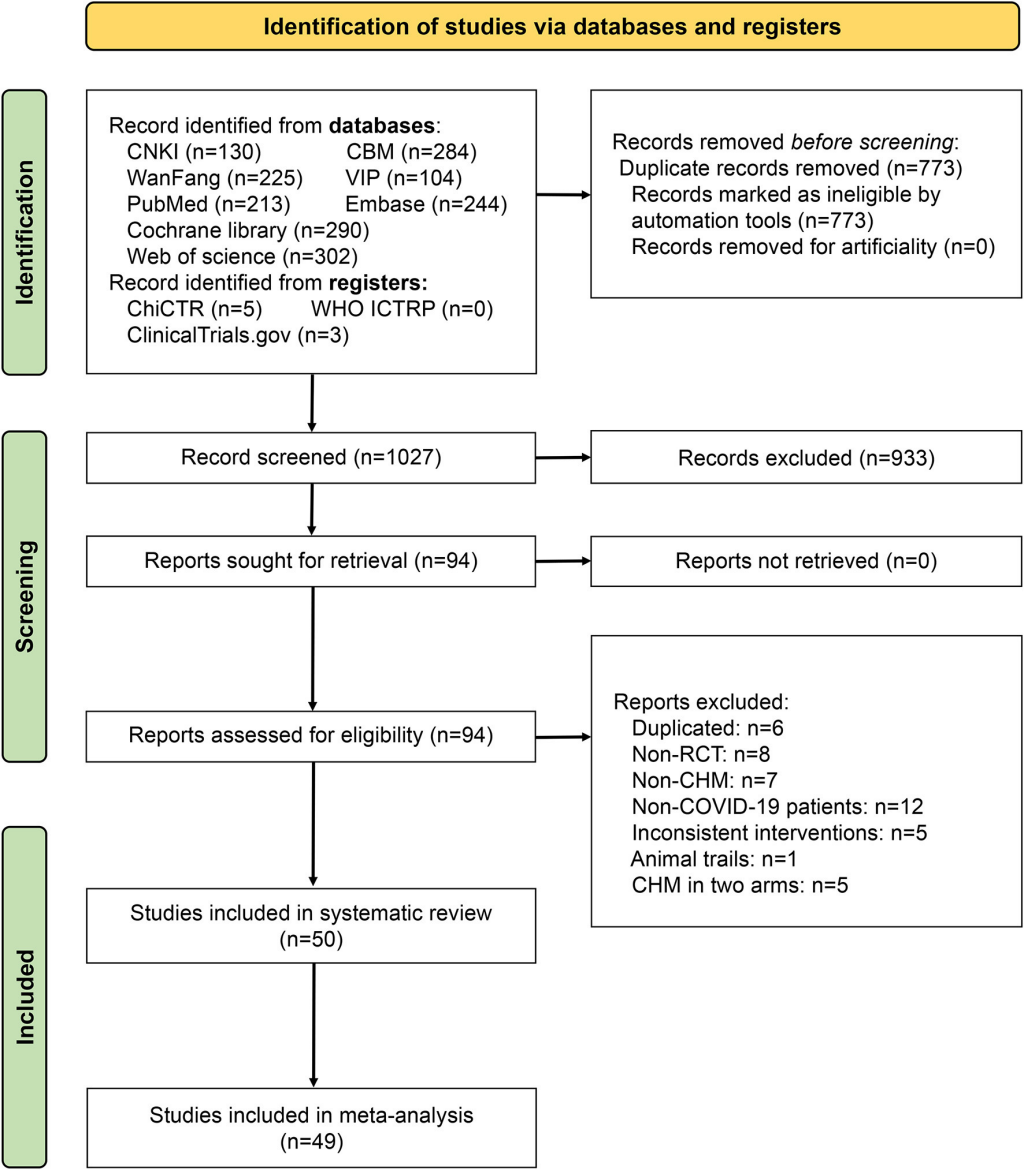
Flow diagram of study search and selection.

A total of 1,800 studies were selected from eight databases and three registers. A total 773 of which were deleted because of duplication, and 933 were excluded for their titles and abstracts. In 94 studies were assessed for eligibility by reading the full texts, and 44 of which were excluded for the following reasons: (a) duplicated (*n* = 6); (b) non-RCT (*n* = 8); (c) non-CHM (*n* = 7); (d) non-COVID-19 patients (*n* = 12); (e) inconsistent interventions (*n* = 5); (f) animal trails (*n* = 1); (g) CHM in two arms (*n* = 5). Our systematic review included 50 studies, 49 of which were included in the meta-analysis (Figure 1), 21 were in English, while the rest were in Chinese. Publication times (online) ranged from 2020 to 2023. The funding sources for the included studies were summarized in Supplementary Table S2.

### Study characteristics

**Figure 2:**
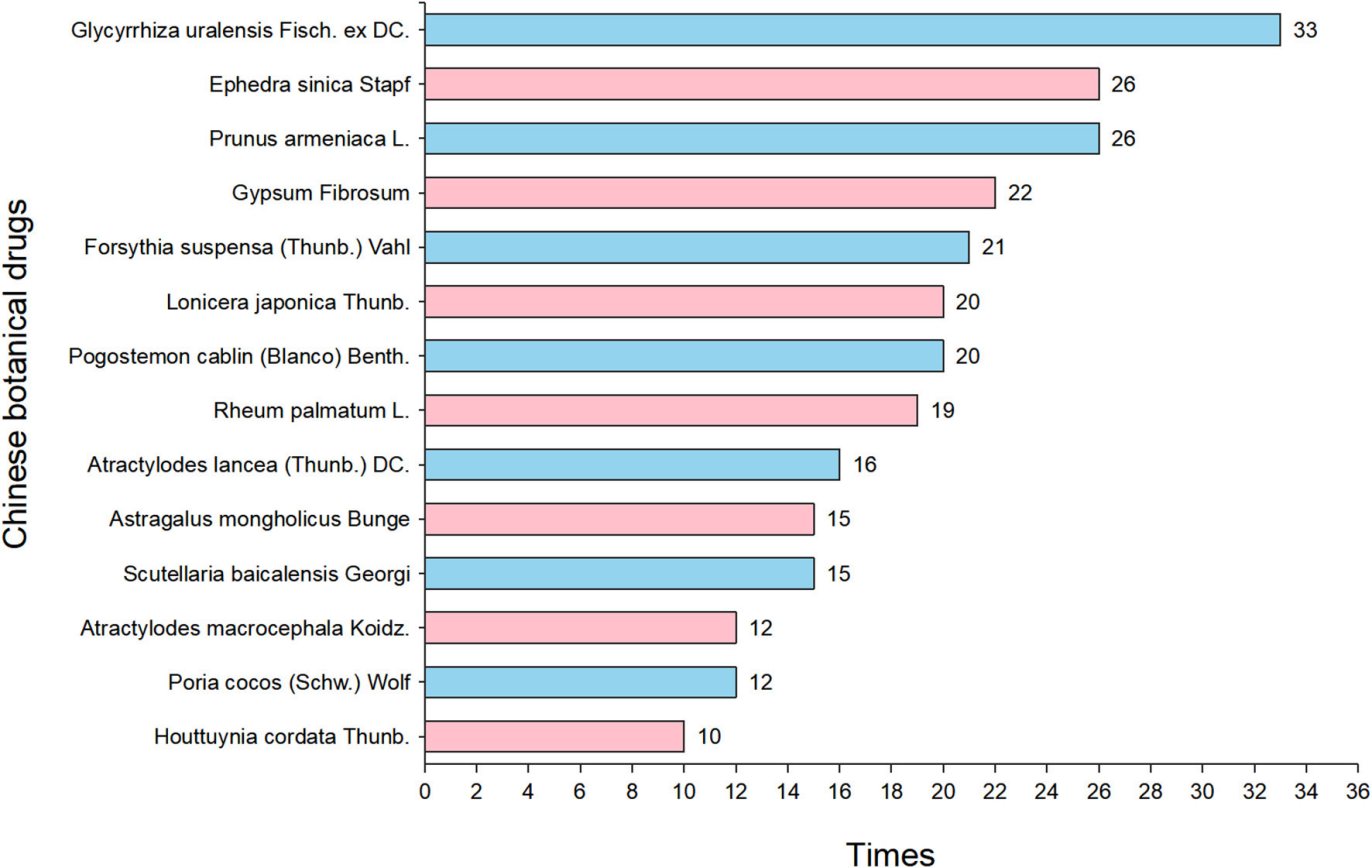
Summary of Chinese herbs use frequency.

The details of the study design and participants of included studies are shown in Table 1. A total of 11,624 participants were randomized, 11,377 of whom were included in the final analysis, the others were rejected for drop-out, refusing medication, violation of the protocol, or for other reasons. The sample size of included studies ranged from 12 to 3,243, and all of them were from China. The severity of disease in participants was categorized into the level of low, moderate, severe, or critical. One of the studies enrolled asymptomatic COVID-19 patients (42). Among the 50 included studies, 32 were single-center studies, 17 were multi-center studies, and 1 did not report the study design. CHM plus CWM treatment was used in intervention groups and the same CWM treatment in control groups for all of the 50 studies. Information of the name, detailed dosage, use frequency, and administration method of medicine was shown in Table 2. Duration of treatment ranged from 5 to 21 days. In the treatment, 29 RCTs employed Chinese patent medicine, 19 used CHM formulas, and 2 used a combination of both. Totally, CHMs in the 50 included studies have 7 dosage forms, including powder, pill/tablet, decoction, oral liquid, granule, capsule, and injection. The following CHM prescriptions were repeated in the included studies: Lianhua Qingwen capsule (granule) (LHQW), Lianhua Qingke capsule (tablet) (LHQK), Jinhua Qinggan granule (JHQG), Xuebijing injection (XBJ), Huashi Baidu granule (HSBD), Qingfei Paidu decoction (QFPD), Jinyinhua oral liquid (JYH), Maxing Shigan decoction (MXSG), Buzhong Yiqi decoction (BZYQ), and so on. Names of Chinese botanical drugs were consulted in the *Chinese pharmacopoeia* version 2020 (https://db.ouryao.com) and https://mpns.science.kew.org, and the detailed information of the specific components and contents of CHM prescriptions was shown in Supplementary Table S3. A total of 130 Chinese botanical drugs were used in all CHMs, with *Gancao* (Glycyrrhiza uralensis Fisch. ex DC.) being the most frequently used option. Botanical drugs with use frequency over ten times were shown in Figure 2.

**Table 1:** Study design and participants' details of included studies.

**References**	**Study type**	**Severity of involved participants**	**Sample size (randomized/analyzed)**	**I/C (M/F)**	**Age (yrs)**	**Original places of participants**
Ai et al. (49)	Single-center	Mild/Moderate/ Severe	98/98	I: 55 (24/31) C: 43 (17/26)	I: 43.98 ± 12.6 C: 45.95 ± 18.3	Guangzhou
Chen et al. (46)	Single-center	Mild/Moderate	60/57	I: 30 (17/13) C: 30 (18/12)	I: 50.16 ± 5.11 C: 49.52 ± 5.06	Shenzhen
Duan et al. (47)	Single-center	Mild	123/102	I: 82 (39/43) C: 41 (23/18)	I: 51.99 ± 13.88 C: 50.29 ± 13.17	Wuhan
Fu et al. (69)	Single-center	Moderate	73/73	I: 37 (19/18) C: 36 (19/17)	I: 45.26 ± 7.25 C: 44.68 ± 1.35	Guangzhou
He et al. (70)	Single-center	Mild	72/71	I: 36 C: 35	NA	Wuhan
Hu et al. (65)	Multi-center	Moderate	300/187	I1 (60 mL): 100 (46/54) I2 (120 mL): 100 (49/51) C: 100 (55/45)	I1: 44.02 ± 13.29 I2: 47.00 ± 14.06 C: 49.28 ± 11.14	Wuhan, Xiaogan, Xianning
Hu et al. (50)	Multi-center	NA	284/284	I: 142 (79/63) C: 142 (71/71)	I: 50.4 ± 15.2 C: 51.8 ± 14.8	China
Liao et al. (90)	Single-center	NA	70/70	I: 35 (20/15) C: 35 (18/17)	I: 65.25 ± 7.42 C: 67.16 ± 8.64	Xishuangbanna
Liu et al. (82)	Single-center	Moderate	204/195	I: 99 (36/63) C: 96 (37/59)	^*^I: 56.00 (48.50–62.00) C: 56.50 (48.75–62.25)	Wuhan
Liu et al. (72)	Single-center	Mild	88/88	I: 44 (16/28) C: 44 (15/29)	I: 48.51 ± 4.56 C: 48.43 ± 4.52	Wuhan
Luo et al. (53)	Single-center	Severe	60/57	I: 29 C: 28	I: 60.26 ± 15.62 C: 56.35 ± 18.28	Jingzhou
Xu et al. (43)	Multi-center	Mild/Moderate/ Severe	157/157	I: 77 (43/34) C: 80 (44/36)	I: 49.1 ± 15.7 C: 50.4 ± 16.0	China
Yang et al. (74)	Multi-center	Mild/Moderate	60/60	I: 30 (16/14) C: 30 (17/13)	I: 45.83 ± 3.72 C: 45.27 ± 3.69	Shandong
Ye et al. (58)	Single-center	Severe	42/42	I: 28 (3/25) C: 14 (4/10)	^*^I: 65 (53.5–69) C: 59 (47–67)	Wuhan
Ye et al. (75)	Single-center	Moderate	100/100	I: 50 (25/25) C: 50 (23/27)	I: 43.32 ± 10.21 C: 42.64 ± 11.39	Wuhan
Yu et al. (76)	Single-center	Mild/Moderate	295/295	I: 147 (82/65) C: 148 (89/59)	I: 48.27 ± 9.56 C: 47.25 ± 8.67	Wuhan
Zeng et al. (83)	Single-center	Mild/Moderate	59/59	I: 30 (19/11) C: 29 (21/8)	I: 50.7 ± 12.3 C: 53.3 ± 15.8	Wenzhou
Zhang et al. (89)	NA	Moderate	120/120	I: 80 (50/30) C: 40 (23/17)	I: 53.4 ± 13.70 C: 52.0 ± 14.10	Hubei
Zhang et al. (84)	Multi-center	Mild/Moderate	130/130	I: 65 (32/33) C: 65 (28/37)	I: 44.31 ± 13.45 C: 48.25 ± 14.22	Ganzhou, Ji'an, Fengcheng, Nanchang
Zhao et al. (79)	Single-center	Severe	40/39	I: 15 (8/7) C: 24 (14/10)	NA	Hefei
Zheng et al. (81)	Multi-center	Moderate/ Critical	130/130	I: 65 (42/23) C: 65 (44/21)	I: 17–84 (range) C: 18–85 (range)	Wuhan
Zhou et al. (6)	Multi-center	Severe/Critical	122/111	I: 57 (33/24) C: 54 (38/16)	^*^66 (56.0–72.0)	Wuhan, Huangshi
Ni et al. (51)	Multi-center	Mild/Moderate/ Severe	235/235	I1 (Low-dose): 54 (23/31) I2 (Middle-dose): 61 (33/28) I3 (High-dose): 59 (49/10) C: 59 (25/34)	I1: 54.00 (42.00–62.25) I2: 56.00 (44.00–65.00) I3: 53.00 (41.50–63.00) ^*^C: 51.00 (38.50–65.00)	Wuhan, Harbin, Nanjing, Hefei
Ping et al. (56)	Single-center	Mild/Moderate	60/54	I: 30 (16/14) C: 24 (10/14)	I: 40.75 (23–58) mean (IQR) C: 41.22 (25–64) mean (IQR)	Jiujiang
Qiu et al. (86)	Single-center	Moderate	50/50	I: 25 (13/12) C: 25 (14/11)	I: 53.35 ± 18.35 C: 51.32 ± 14.62	Chongqing
Sun et al. (52)	Multi-center	Mild/Moderate	57/57	I: 32 (17/15) C: 25 (11/14)	I: 45.4 ± 14.10 C: 42.0 ± 11.70	Tangshan, Hengshui, Cangzhou
Sun et al. (66)	Single-center	Moderate/ Severe	80/80	I: 40 (20/20) C: 40 (14/26)	I: 63.08 ± 9.97 C: 60.28 ± 12.64	Wuhan
Tan et al. (44)	Multi-center	Mild	66/66	I: 33 (19/14) C: 33 (17/16)	I: 42.53 ± 0.23 C: 42.53 ± 0.12	Guangzhou
Wang et al. (87)	Single-center	Moderate	30/30	I1 (CHM): 10 (5/5) I2 (CHM+Vc): 10 (4/6) C: 10 (5/5)	I1: 39.2 ± 10.01 I2: 54.90 ± 3.61 C: 55.90 ± 3.71	Xi'an
Wang et al. (42)	Single-center	Moderate/ Asymptomatic	38/38	I1 (Moderate): 11 (6/5) I2 (Asymptomatic): 8 (5/3) C1 (Moderate): 11 (5/6) C2 (Asymptomatic): 8 (4/4)	I1: 43.43 ± 17.51 C1: 41.73 ± 15.16 I2: 30.11 ± 17.94 C2: 36.32 ± 12.43	Shijiazhuang
Wang et al. (73)	Single-center	Moderate	140/140	I: 70 (35/35) C: 70 (36/34)	I: 48 ± 13.2 C: 49.4 ± 13.3	Xiangyang
Wen et al. (88)	Single-center	Severe	60/60	I: 40 (23/17) C: 20 (9/11)	I1 (50 mL): 49.1 ± 4.8 I2 (100 mL): 47.1 ± 5.2 C: 47.7 ± 5.7	Changsha
Xiong et al. (91)	Single-center	Mild/Moderate/ Severe	42/42	I: 22 C: 20	I: 57.10 ± 14.00 C: 62.40 ± 12.30	Wuhan
An et al. (55)	Multi-center	Mild/Moderate	34/32	I: 24 C: 8	NA	Wuhan
Li et al. (71)	Multi-center	Severe	12/12	I: 6 (2/4) C: 6 (3/3)	I: 52.00 ± 6.56 C: 50.00 ± 10.00	Shanxi
Xiao et al. (57)	Multi-center	NA	188/182	I: 119 (68/51) C: 63 (35/28)	I1 (LHQW): 54.58 ± 13.76 I2 (LHQW + HXZQ): 54.31 ± 11.63 C: 54.06 ± 13.90	Wuhan
Wang et al. (48)	Single-center	NA	48/47	I: 24 (14/10) C: 23 (12/11)	I: 46.8 ± 14.4 C: 51.4 ± 17.6	Beijing
Zhang et al. (77)	Multi-center	Mild/Moderate	144/144	I: 72 (23/49) C: 72 (24/48)	I: 49.56 ± 14.88 C: 52.81 ± 14.83	Shijiazhuang, Xingtai, Harbin
Zhao et al. (78)	Single-center	Mild	408/408	I: 204 (88/116) C: 204 (94/110)	I: 52.0 (39.0, 58.0) C: 49 (37.8, 58.0)	Wuhan
Chai et al. (68)	Multi-center	Mild/Moderate/ Severe/Critical	137/137	I: 96 C: 41 (21/20)	I: NA C: 49.68 ± 12.43	Zhejiang
Yang et al. (85)	Single-center	Mild/Severe	60/60	I1 (CHM): 20 (11/9) I2 (CHM+Vc): 20 (11/9) C: 20 (9/11)	I1: 50.2 ± 9.6 (Mild) 49.0 ± 7.1 (Severe) I2: 47.9 ± 10.1 (Mild) 50.3 ± 9.5 (Severe) C: 47.7 ± 8.9 (Mild) 46.1 ± 9.2 (Severe)	Xi'an
Zhao et al. (80)	Multi-center	Moderate	96/96	I: 51 (26/25) C: 45 (30/15)	I: 46.81 ± 9.89 C: 45.07 ± 8.97	Jingzhou
Soleiman et al. (54)	Multi-center	Moderate	213/195	I: 91 (66/28) C: 104 (74/30)	I: 52.7 ± 19.6 C: 54.6 ± 15.2	Khanevadeh, Be'sat, Golestan, and Imam Reza hospitals
Wang et al. (45)^¶^	Single-center	Mild	3,243/3,243	I: 667 (493/174) C: 2,576 (1,844/732)	I: 42.1 ± 13.1 C: 43.5 ± 12.4	Shanghai
Zhang et al. (64)^¶^	Single-center	Mild	240/234	I: 117 (62/55) C: 117 (63/54)	I: 41.8 ± 9.8 C: 41.2 ± 13.2	Shanghai
Chen et al. (63)	Single-center	Moderate	60/60	I: 30 (17/13) C: 30 (16/14)	I: 34.57 ± 8.96 C: 36.50 ± 9.76	Xiaogan
Hu et al. (62)	Single-center	Initial heat-up phase of COVID-19	86/86	I: 43 (19/24) C: 43 (20/23)	I: 49.05 ± 9.72 C: 49.53 ± 10.68	Zhejiang
Wang et al. (61)	Single-center	Severe/Critical	135/120	I: 80 (42/38) C: 40 (26/14)	I: 65.50 (54.00, 74.75) C: 69.00 (56.00, 73.00)	Hubei
Xu et al. (59)^¶^	Single-center	Mild	2,830/2,800	I: 1,411 (823/588) C: 1,407 (839/568)	I: 47 (33, 55) C: 45 (32, 56)	Shanghai
Zhang et al. (60)^¶^	Single-center	Mild	145/144	I: 97 (52/45) C: 47 (22/25)	I: 67.0 ± 14.0 C: 66.0 ± 11.4	Shanghai

* Median (interquartile range); IQR, interquartile range;

¶ Patients infected with SARS-CoV-2 Omicron variant; LHQW, Lianhua Qingwen; HXZQ, Huoxiang Zhengqi; I, Chinese herbal medicine plus conventional western medicine group; C, conventional western medicine group; M, male; F, female; NA, not applicable; Vc, vitamin C.

**Table 2:** Treatment details and outcomes of included studies.

**References**	**Intervention**	**Control**	**Treatment duration (days)**	**Adverse reactions report**	**Outcome measures**
Ai et al. (49)	“Pneumonia No. 1” granules (100 mL, bid, po) + C	Antiviral therapy including abidor, Lopinavir and ritonavir, chloroquine; Symptomatic therapy including oxygen therapy, anti-inflammatory therapy and expectorant treatment	12	Y	①④⑨⑩
Chen et al. (46)	Lianhua Qingwen capsule (4 capsules, tid, po) + C	CWM including lopinavir and ritonavir tablets (2 pills, bid, po), interferon-α2b (5 million IU, bid, inhal)	10	Y	②⑤⑧⑩
Duan et al. (47)	Jinhua Qinggan granules (10 g, tid, po) + C	Antiviral therapy and antibacterial therapy	5	Y	⑤⑨
Fu et al. (69)	Toujie Quwen granules (bid, po) + C	CWM including abidor (0.2 g, tid, po), ambroxol (30 mg, tid, po).	15	Y	①⑤⑨
He et al. (70)	Buzhong Yiqi decoction (0.5 dose, bid, po) + C	Abidor (0.2 g, tid, po)	10	N	①⑨⑩
Hu et al. (65)	Jinyinhua oral liquid (60 mL/120 mL, tid, po) + C	CWM including lopinavir and ritonavir tablets (0.6 g, bid, po), interferon-α2b (5 million IU, bid, im)	10	Y	②④⑤
Hu et al. (50)	Lianhua Qingwen capsules (4 capsules, tid, po) + C	Supportive treatment such as oxygen therapy, antiviral medications and symptomatic therapies	14	Y	①②③⑤⑧
Liao et al. (90)	CHM decoction (1 dose, qd, po) + C	Symptomatic therapy, oxygen therapy, antiviral therapy, lopinavir (2 pills, bid, po).	7	Y	NA
Liu et al. (82)	Huashi Baidu granule (10 g, bid, po) + C	Standard care in accordance with the NHC-NATCM-China guidelines	14	Y	②③⑤⑧
Liu et al. (72)	Lianhua Qingwen capsule (1.4 g, tid, po) + “Pneumonia No. 2” formula (0.5 dose, bid) + C	CWM including abidor (0.2 g, tid, po) and oseltamivir (0.015 g, bid, po)	21	Y	①
Luo et al. (53)	Xuebijing injection (50 mL, bid) + C	Nutritional support, oxygen therapy, antiviral therapy with interferon-inhalation, antibiotic agents, noninvasive and invasive ventilation if necessary	14	Y	⑤⑥⑦⑧⑩
Xu et al. (43)	Reduning injection (20 mL, qd, iv) + C	Supportive treatment (oxygen), antiviral treatment, and symptomatic treatment	14	Y	②④⑦⑧
Yang et al. (74)	CHM decoction (200 mL, bid, po) + C	CWM treatment according to the “Diagnosis and Treatment Protocol for COVID-19 (Trial version 6)”	14	N	①
Ye et al. (58)	CHM decoction (200 mL, bid, po) + C	Hemodynamic monitoring, laboratory testing, supplementary oxygen, intravenous fluids, and routine pharmaceutical medications and other medical care when deemed appropriate by on-duty physicians. Oral ribavirin/arbidole (not remdesivir) was part of the standard care	7	N	①③⑤⑥⑨⑩
Ye et al. (75)	Modified Shengjiang Powder (0.5 dose,bid,po)+C	Abidor (0.2 g, tid, po)	6	Y	①⑩
Yu et al. (76)	Lianhua Qingwen granules (6 g, tid, po) + C	Abidor (0.2 g, tid, po), moxifloxacin hydrochloride tablets (0.4g, qd, po) and ambroxol hydrochloride tablets (30 mg, tid, po) Ambroxol Hydrochloride Dispersible Tablets (30 mg, bid, po)	7	Y	①③⑤⑦⑩
Zeng et al. (83)	Maxingshigan-Weijing decoction (200 mL, bid, po) + C	Routine supportive care alone including staying in bed, oxygen therapy provided by a nasal cannula, broad-spectrum antibiotics and antivirals	14	Y	②④⑤⑧⑨⑩
Zhang et al. (89)	Jinyinhua oral liquid (60 mL, tid, po) + C	CWM including lopinavir and ritonavir tablets (2 pills, bid, po), α-Interferon (5 million U, bid, im)	10	Y	⑤
Zhang et al. (84)	Xiyanping injection (10 mg/kg, the maximum daily dose ≤ 500 mg, qd,iv) + C	Standard symptomatic treatments including supplemental oxygen therapy, antiviral medicines, antibiotic agents and immune modulators	7-14	Y	⑤⑤⑧
Zhao et al. (79)	Yidu-toxicity blocking lung decoction + C	CWM treatment including bed rest and supportive treatments, ensuring sufficient calories and water intake, maintaining water electrolyte balance and homeostasis	14	N	①④⑧⑩
Zheng et al. (81)	Xiaochaihu decoction with Maxing Shigan decoction (100 mL, tid, po)/Sanren decoction (100 mL, tid, po) + C	CWM including α-interferon (inhal), lopinavir and ritonavir tablets (po), abidor (po), moxifloxacin, whey protein powder, methylprednisolone, warm saline for patients with diarrhea (1,000 mL/day, po)	14	N	①
Zhou et al. (67)	Shenhuang granule (2 bags/day) + C	CWM treatment including injection of interferon -α2b, receiving lopinavir–ritonavir, receiving vasopressors, renal replacement therapy, highest oxygen therapy support (non-invasive mechanical ventilation, invasive mechanical ventilation, extracorporeal membrane oxygenation or mechanical ventilation, antibiotic, corticosteroids therapy after trial enrollment, other oral patent CHM product)	14	Y	①⑤⑥⑦
Ni et al. (51)	Shuanghuanglian oral liquids (20 mL/40 mL/60 mL, tid, po) + C	Supportive treatments including supplemental oxygen therapy, daily symptom and vital sign monitoring, clinical laboratory testing, correction of water, electrolyte and acid base imbalances, and administration of antiviral agents and antibiotic agents if bacterial infection was found	14	Y	④⑩
Ping et al. (56)	Jiawei Yupingfeng powder (bid, po) +C	CWM including lopinavir and ritonavir tablets (400 mg/100 mg, bid, po) and α-interferon (bid, inhal)	14	Y	①②④⑩
Qiu et al. (86)	Maxing Xuanfei Jiedu decoction (150 mL, tid, po) + C	CWM including lopinavir and ritonavir tablets (400 mg/100 mg, bid, po) and α-interferon (bid, inhal)	10	N	③⑤⑧⑨
Sun et al. (52)	Lianhua Qingke granule (1 bag, tid, po) + C	CWM including lopinavir and ritonavir tablets (400 mg/100 mg, bid, po) and α-interferon (bid, inhal)	14	Y	③⑤⑧
Sun et al. (66)	Liu Shen capsule (31.25 mg, tid, po) + C	Abidor (200 mg, tid, po)	7	Y	①⑧⑨
Tan et al. (44)	Lianhua Qingwen capsule (1.4 g, bid, po) + C	Antiviral therapy (interferon, abidor, ritonavir, etc.), antibacterial therapy, anti-inflammatory therapy (glucocorticoid), immunoregulatory therapy, oxygen therapy, invasive and non-invasive mechanical ventilation, ECMO, symptomatic and supportive treatment	NA	N	①
Wang et al. (87)	T1: CHM decoction (bid, po) + C T2: CHM decoction (bid, po) + vitamin C (10 g/60 kg, bid, iv) + C	CWM including ribavirin, antibacterial drugs and supportive drugs	7	N	③
Wang et al. (42)	Qingre Kangdu oral liquid (20 mL, tid, po) + C	CWM including interferon-α2b (bid, inhal) and arbidor (0.2 g, tid, po)	10	N	②③④⑧
Wang et al. (73)	Qingfei Paidu decoction (100 mL, bid, po) + C	Symptomatic treatment, nutritional support, antiviral therapy, antibacterial therapy, moxifloxacin (0.4 g, qd, po), arbidor (0.2 g, tid, po)	10	Y	①④⑨⑩
Wen et al. (88)	Xuebijing injection (50 mL/100 mL, bid, po) + C	CWM treatment according to the “Diagnosis and Treatment Protocol for COVID-19”	7	Y	⑤⑥⑩
Xiong et al. (91)	Xuanfei Baidu decoction (200 mL, bid, po) + C	CWM treatment according to the “COVID-19 Prevention and Control Program (Trial)”	7	Y	NA
An et al. (55)	Jinhua Qinggan granules (5g, tid, po) + C	Antiviral therapy including treatment with oseltamivir (75 mg, qd, po), abidor (200 mg, tid, po), for antimicrobial therapy, bacteriological monitoring was intensified, and antimicrobial drugs were administered promptly when there was evidence of secondary bacterial infection; penicillin, cephalosporins, floxacins, and macrolides were administered orally	14	N	⑤
Li et al. (71)	Qingfei Paidu decoction (bid, po) + C	Symptomatic and supportive treatment including nutritional support, maintenance of water, electrolytes, acid-base balance, relieving cough and reducing sputum, antibacterial drugs and antiviral drugs (α-interferon, ribavirin)	8	Y	①④⑩
Xiao et al. (57)	T1: LHQW: Lianhua Qingwen granules (1 bag, tid, po) + C T2: LHQW + HXZQ:Huoxiang Zhengqi dropping pills (1 bag, bid, po) + Lianhua Qingwen granules (1 bag, tid, po) + C	CWM including oseltamivir (75 mg, qd, po), abidor (200 mg, tid, po), ribavirin (150 mg, tid, po); antimicrobial therapy: strengthen bacteriological monitoring and use antibiotics when there is evidence of a secondary bacterial infection using oral penicillins, cephalosporins, ofloxacin, and macrolide etc	14	N	⑤
Wang et al. (48)	Keguan-1 (19.4 g, bid, po) + C	CWM including α-interferon (50 μg, bid, inhal) and lopinavir/ritonavir (400 mg/100 mg, bid, po)	14	Y	②③⑦⑧⑩
Zhang et al. (77)	Lianhua Qingke tablets (4 tablets, tid, po) + C	Supportive oxygen therapy, administration of antivirals, and symptom management	14	Y	①③⑦⑧
Zhao et al. (78)	Huashibaidu granule (20 g, bid, po) + C	Bed rest, sufficient food and water intake, frequent monitoring of vital signs, and bedside oxygen therapy if necessary, administration of arbidol hydrochloride	7	Y	①④⑤⑧
Chai et al. (68)	“Pneumonia No. 1” formula/“Pneumonia No. 2” formula/“Pneumonia No. 3” formula (150 mL, tid, po) + C	CWM treatment according to the “Diagnosis and Treatment Protocol for COVID-19 (Trial version 5)”	7–14	Y	①②
Yang et al. (85)	T1: Qi-nourishing essence-replenishing decoction + Hu-Huang decoction + Bai-Mu decoction (50 g, po) + C T2: Hu-Huang decoction (50 g, intal) + vitamin C (10 g, intal)/(10 g/60 kg, bid, iv) + (3 g, tid, po) + vitamin E (200 mg, tid, po) + folic acid (10 mg, tid, po) + C	CWM treatment according to the “Diagnosis and Treatment Protocol for COVID-19 (Trial version 5)”	NA	Y	②⑩
Zhao et al. (80)	“Antivirus No.1” formula (450 mL/day, po) + C	Bed rest, supportive treatment, ensure sufficient calories, Maintain the steady state of internal environment, Monitor vital signs and oxyhemoglobin saturation of finger tip, etc CWM including α-interferon (3 million U, bid, intal) and lopinavir and ritonavir tablets (2 tablets, bid, po)	9	N	①③⑨⑩
Zhang et al. (60)^¶^	Ganjiang Xiaochaihu decoction (qd, po) + C	Basic therapy including controlling the underlying disease and giving the necessary symptomatic treatment when the condition changes	5	N	①②⑨
Wang et al. (61)	CHM decoction (200ml, bid, po)/Chinese medicine injection/Oral Chinese patent medicine + C	Basic therapy including anti-inflammatory, antispasmotic, antiasthmatic, expectorant, correction of water and electrolyte balance, selection of antiviral and antibacterial drugs according to auxiliary examination and clinical experience, and invasive or non-invasive assisted ventilation	7	Y	①②⑦⑨⑩
Hu et al. (62)	CHM decoction (200 ml, bid, po) + C	All patients were treated with conventional western medicine, including basic treatment: bed rest, attention to water and electrolyte balance, close monitoring of vital signs and blood oxygen saturation, and selective use of high-flux oxygen assistance, non-invasive mask ventilation, low tidal volume lung protective ventilation and other auxiliary treatment according to the condition Oseltamivir phosphate capsules (75 mg, tid, po)/Lopinavir ritonavir tablets (2 tablets, tid, po)/ribavirin injection (0.5 g, bid/tid, iv)/Arbidol hydrochloride granules (0.2 g, tid, po) and Levofloxacin tablets (0.5 g/tablet)/cefprozil tablets (0.25 g/tablet)	14	Y	①④⑤⑦⑧
Chen et al. (63)	CHM decoction (200 ml, bid, po) + C	Antiviral treatment, Recombinant human interferon α1b injection (5 million units, tid, intal)/Lopinavir-ritonavir tablets (400 mg/100 mg, tid, po)/Ribavirin injection (500 mg, tid, po)/Chloroquine phosphate tablets (500 mg, tid, po)/Arbidol hydrochloride tablets (200 mg, tid, po)	7	N	①②③⑧⑩
Xu et al. (59)^¶^	Reyanning mixture (20 ml, qid, po) + C	Standard treatments including physical condition monitoring, antiviral, antibacterial, symptomatic treatment, and underlying disease treatment	7	Y	②④⑤
Zhang et al. (64)^¶^	Shufeng Jiedu capsule (4 capsules, tid, po) + C	Best supportive care	7	Y	①②⑥
Wang et al. (45)^¶^	Longyizhengqi granule (5 g, bid, po) + C	Conventional treatment including rest in bed, strengthening supportive treatment, close monitoring of vital signs, and specified effective oxygenation measures.	NA	Y	②④
Soleiman et al. (54)	Licorice syrup (10 ml, tid, po) + C	Standard treatment including hydroxychloroquine plus lopinavir/ritonavir, oxygen supplement, and analgesics	7	Y	④⑤⑦

① clinical efficacy; ②SARS-CoV-2 nucleic acid conversion time; ③chest image improvement; ④duration of hospitalization; ⑤conversion to severe cases; ⑥conversion to mild cases; ⑦death; ⑧clinical symptoms recovery time; ⑨total score of TCM syndrome; ⑩laboratory indicators. C, control; qd, once per day; bid, twice per day; tid, three times per day; po, take orally; im, intramuscular injection; iv, intravenous injection; intal, inhalation; NA, not applicable; Y, yes; N, no; ECMO, extracorporeal membrane oxygenation.

¶ Patients infected with SARS-CoV-2 Omicron variant.

### Risk of bias

**Figure 3:**
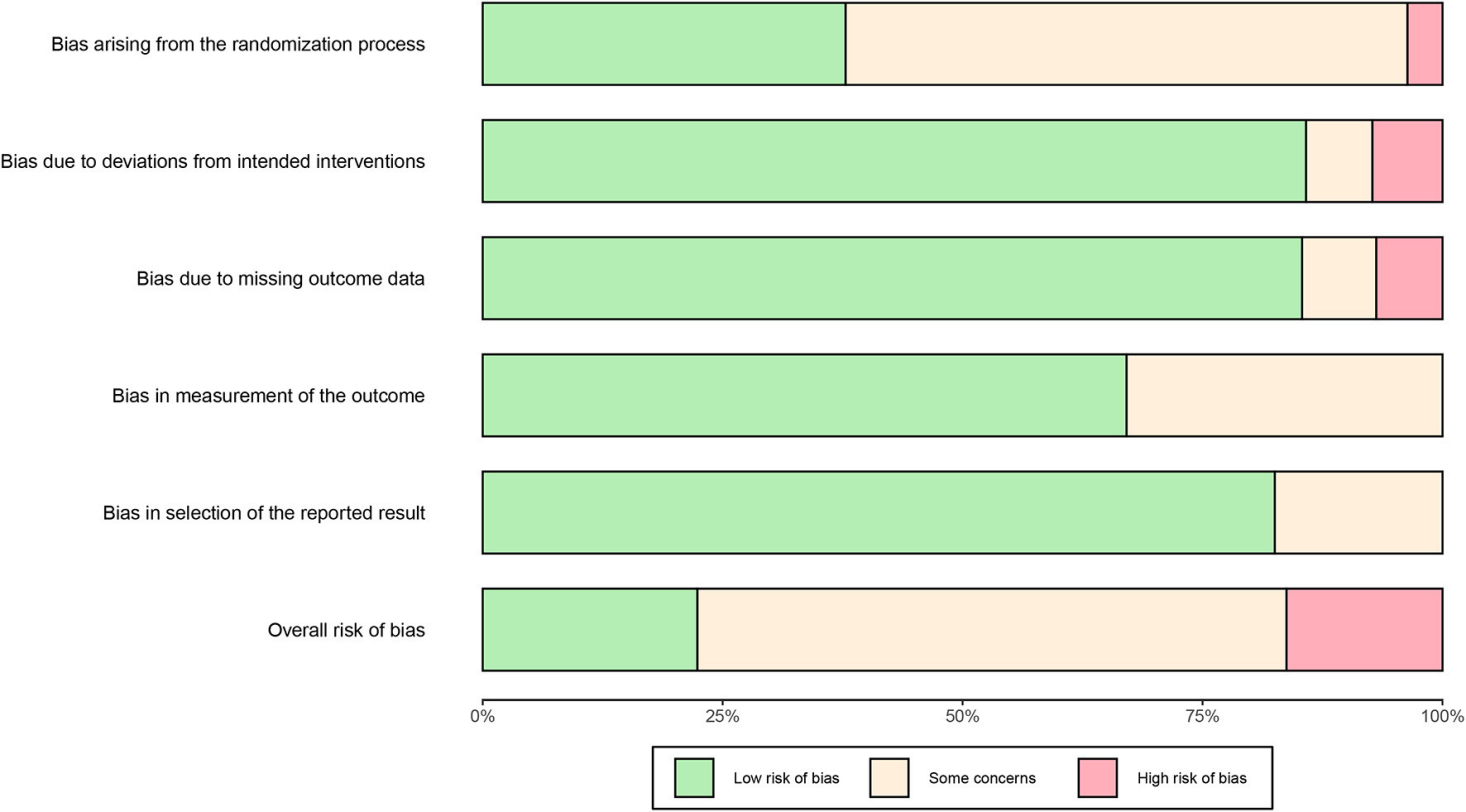
Risk of bias graph.

The assessment results of the risk of bias are shown in Figure 3 and Supplementary Table S4. Totally, 246 outcomes were assessed in 50 studies, a majority of which was at a moderate level of risk of bias: 55 outcomes (22.4%) were categorized into “low risk”, 151 (61.4%) into “some concerns” and 40 (16.3%) into “high risk”.

- Bias arising from the randomization process

As 29 studies failed to mention their allocation concealment implementation, 144 outcomes were determined to be “some concerns” in this domain. Additionally, three studies (43–45) grouped the participants using inappropriate randomization methods, as a result, 9 outcomes were assessed as “high risk” in the three studies.

- Bias due to deviations from intended interventions

Three studies reported deviations from intended interventions, or estimated the effects of assignment to intervention by inappropriate analysis (46–48); therefore, 17 outcomes were assessed as being of “some concerns”. Four studies deviated from the intended intervention on a considerable extent (49–52), leading to imbalances between the two arms, consequently, 14 outcomes were determined to be “high risk”. As failure to appropriately analyze participants in the groups may impose substantial impacts on the results, the results “death” in one study (48) and “conversion to severe cases” in another (46) were evaluated as “high risk” in this domain.

- Bias due to missing outcome data

In total of 19 outcomes were determined to be “some concerns” in the domain of missing outcome data, due to 4 studies failed to report the complete or near complete outcome data from all participants (46, 47, 53, 54). Seventeen outcomes were classified as “high risk” in 8 studies, as missing data in the outcomes might depend on their true values (50, 53–59).

- Bias in measurement of the outcome

As no blind methods were adopted or no information about blind methods were mentioned in 31 studies, 81 outcomes were identified to be “some concerns” in the domain of outcome measurement. Despite of the fact that the participants were aware of the interventions they received, some objective outcomes data was free from influence. Thus, these outcomes were evaluated as “low risk” in this domain.

- Bias in selection of the reported result

The majority of the outcomes were evaluated as “low risk” in the domain of selection of the reported result. A total of 43 outcomes in 9 studies were assessed as being of “some concerns” in this domain (46, 47, 50, 55, 56, 60–63).

### Meta-analysis of primary outcomes

#### Clinical effective rate

**Figure 4:**
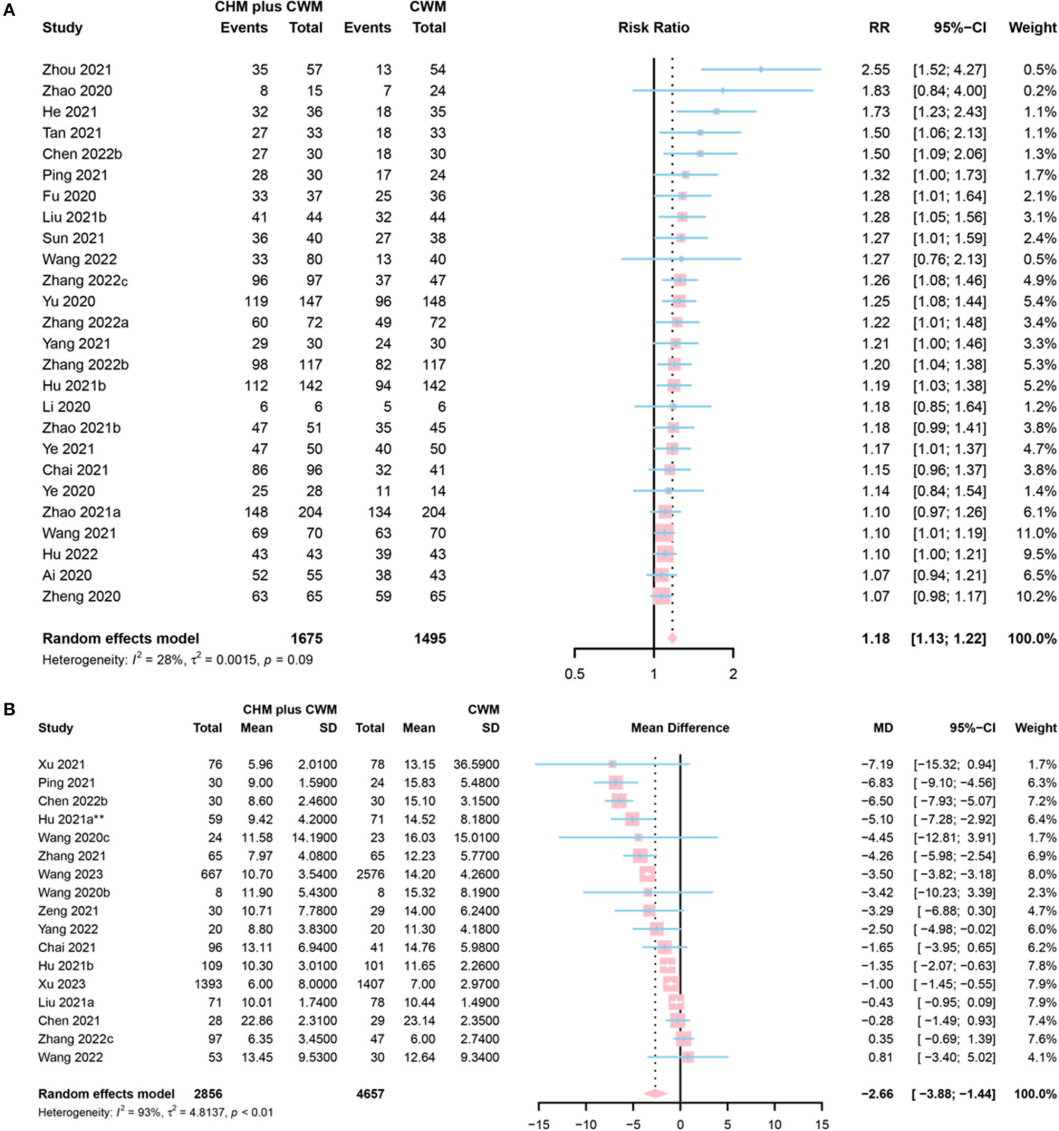
Forest plot of **(A)** clinical efficacy, and **(B)** SARS-CoV-2 nucleic acid conversion time. *Data from the 60 mL CHM group were included in the meta-analysis.

The clinical effective rate is defined as [(the number of cured participants in each group + the number of improved participants in each group)/the total number of participants in each group] × 100%. Among the 26 studies that reported the clinical effective rate, 4 were assessed as “low risk” (58–67), 20 as “some concerns” (56, 68–81) and 2 as “high risk” (44, 49). CHM formulas were given in 13 studies (58, 60–63, 68, 70, 71, 73, 74, 79–81), Chinese patent medicine in 12 studies (44, 49, 56, 64–67, 69, 75–78), and a combination of both in 1 study (72). A total of 1,675 participants involved in CHM plus CWM groups and 1,495 in CWM groups. The meta-analysis results indicated that the clinical effective rate of CHM plus CWM treatment groups was better than that of CWM treatment groups (RR = 1.18, 95% CI [1.13, 1.22], *I*^2^ = 28%, *P* < 0.0001, low certainty) (Figure 4A).

#### SARS-CoV-2 nucleic acid conversion time

A total of 17 studies evaluated SARS-CoV-2 nucleic acid conversion time. Five of them were assessed as “low risk” (59, 65, 82–84), 8 as “some concerns” (41, 42, 46, 48, 60, 61, 63, 85), and 4 as “high risk” (43, 45, 56, 65). Among them, 16 studies enrolled symptomatic cases (41, 43, 45, 46, 48, 56, 59–61, 63, 65, 82–85) and the rest one (42) evaluated nucleic acid conversion time in asymptomatic patients with COVID-19. A total of 2,856 participants involved in CHM plus CWM groups, and 4,657 involved in CWM groups. The results of the meta-analysis showed that CHM plus CWM groups had a shorter SARS-CoV-2 nucleic acid conversion time than those in CWM group (MD = −2.66, 95% CI [−3.88, −1.44], *I*^2^ = 93%, *P* < 0.0001, low certainty) (Figure 4B).

### Meta-analysis of secondary outcomes

#### Chest image improvement

**Figure 5:**
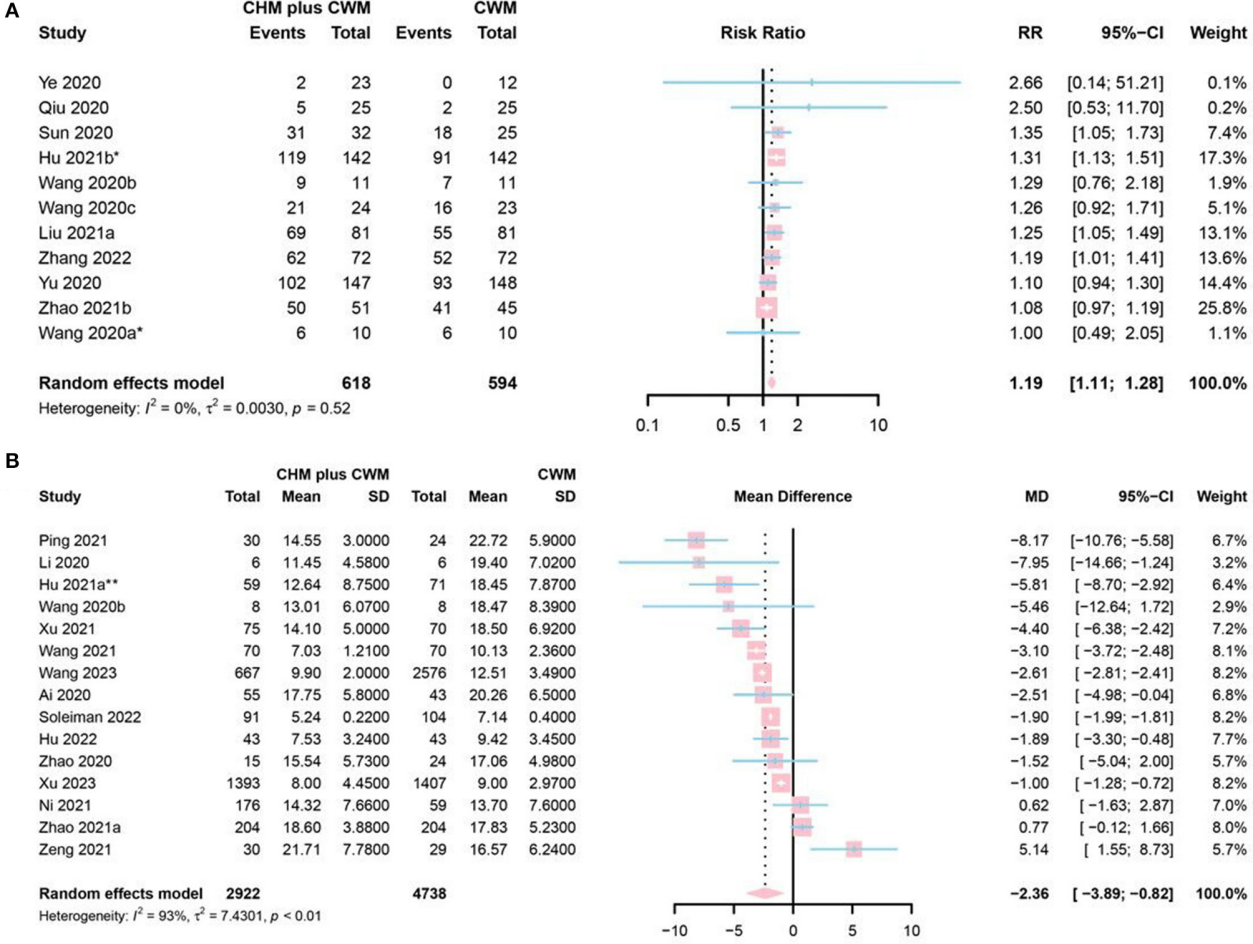
Forest plot of **(A)** chest image improvement, and **(B)** duration of hospitalization. *Data from the oral CHM plus inhalation treatment group were included in the meta-analysis; **Data from the 60 mL CHM group were included in the meta-analysis.

A total of 11 studies mentioned chest image improvement. Two studies were assessed as “low risk” (50, 82), 7 as “some concerns” (42, 48, 76, 77, 80, 86, 97) and 2 as “high risk” (52, 58). A total of 618 participants involved in CHM plus CWM groups, and 594 in CWM groups. The meta-analysis revealed a significantly increasing chest image improvement in CHM plus CWM groups (RR = 1.19, 95% CI [1.11, 1.28], *I*^2^ = 0%, *P* < 0.0001, moderate certainty) (Figure 5A).

#### Duration of hospitalization

Fifteen studies mentioned duration of hospitalization, 1 study identified as “low risk” (83), 8 as “some concerns” (42, 60–62, 71, 73, 78, 79), and 5 as “high risk” (43, 49, 51, 56, 65). A total of 2,922 participants involved in CHM plus CWM groups and 4,738 in CWM groups. The results of the meta-analysis showed that CHM plus CWM groups had a shorter duration of hospitalization than CWM groups (MD = −2.36, 95% CI [−3.89, −0.82], *I*^2^ = 93%, *P* = 0.0026, very low certainty) (Figure 5B).

### Condition of disease conversion

#### Conversion to severe cases

**Figure 6:**
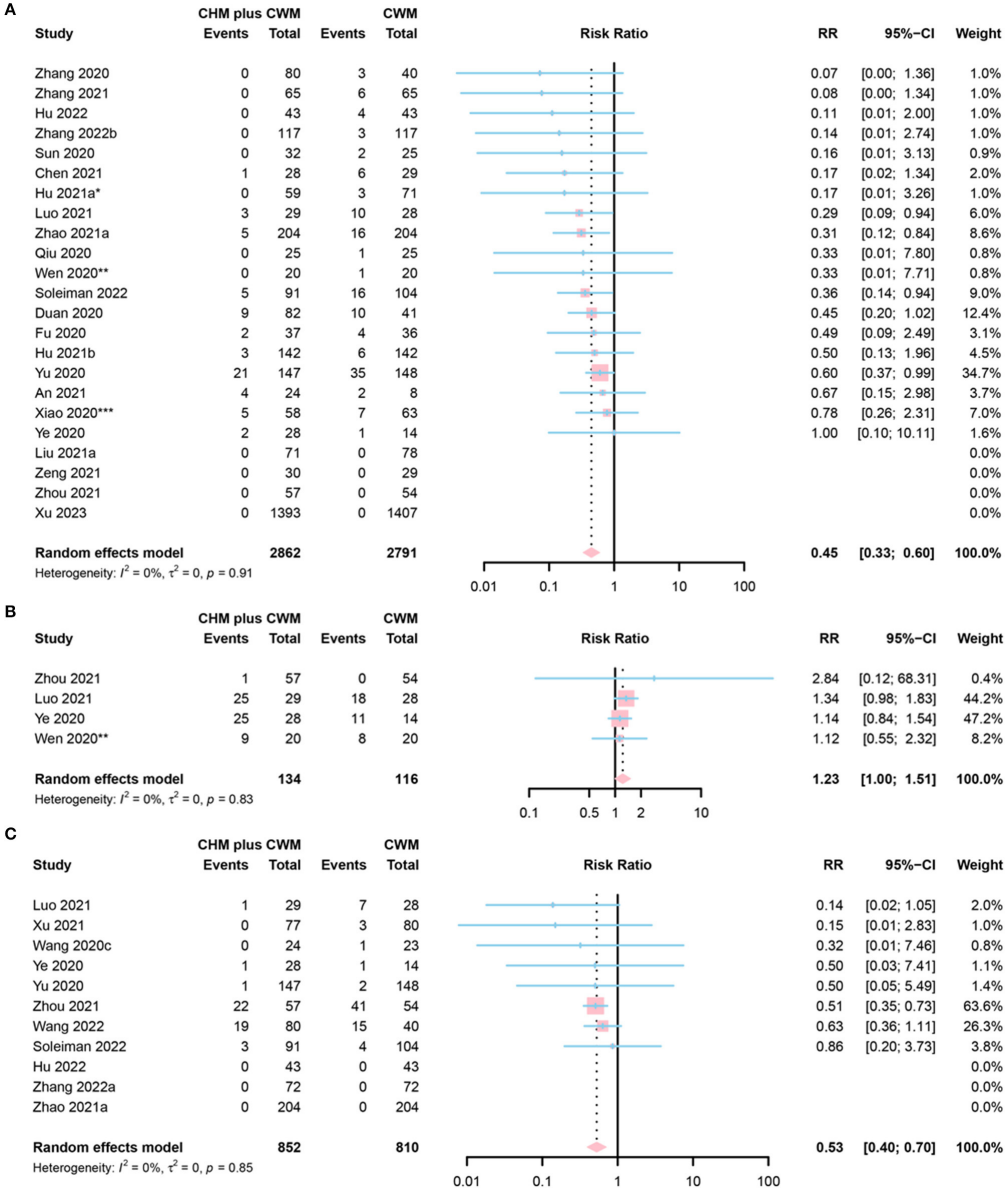
Forest plot of **(A)** conversion to severe cases, **(B)** conversion to mild cases, and **(C)** death. *Data from the 60 mL CHM group were included in the meta-analysis; **Data from the 50 mL CHM group were included in the meta-analysis; ***Data from the Lianhua Qingwen group were included in the meta-analysis.

Conversion to severe cases was defined as (a) changing from mild to moderate, severe or critical condition; (b) changing from moderate to severe or critical condition; or (c) changing from severe to critical condition. A total of 23 studies reported conversion to severe cases. Six of them were assessed as “low risk” (50, 58, 59, 67, 83, 84), 11 as “some concerns” (47, 54, 62, 64, 69, 76, 78, 82, 86, 88, 89), and 6 as “high risk” (46, 52, 53, 55, 57, 65). A total of 2,862 participants involved in CHM plus CWM groups, and 2,791 in CWM group. The disease severity in 4 studies changed from severe to critical (53, 58, 67, 88), and in 16 studies from mild or moderate to severe or critical (46, 47, 52, 54, 55, 59, 62, 64, 65, 69, 76, 82–84, 86, 89). Three studies provided no detailed definition of “rate of conversion to severe cases” (50, 57, 78). The meta-analysis results showed the rate of conversion to severe cases in CHM plus CWM groups was lower than in CWM groups (RR = 0.45, 95% CI [0.33, 0.60], *I*^2^= 0%, *P* < 0.0001, low certainty) (Figure 6A).

#### Conversion to mild cases

Conversion to mild cases was defined as: (a) changing from critical to severe, moderate or mild condition; (b) changing from severe to moderate or mild condition; or (c) changing from moderate to mild condition. Of the 4 studies that reported conversion to mild cases, 3 were assessed as “low risk” (43, 47, 50), and 1 as “some concerns” (71). A total of 134 participants involved in CHM plus CWM groups and 116 in CWM groups. The disease severity in 3 studies changed from severe to mild or moderate (43, 47, 71), and in 1 study from critical to severe (50). The results of the meta-analysis indicated that the rate of conversion to mild cases in CHM plus CWM groups was equivalent to that in CWM groups (RR = 1.23, 95% CI [1.00, 1.51], *I*^2^ = 0%, *P* = 0.0539, low certainty) (Figure 6B).

### Death

The results of the meta-analysis on 11 studies reporting mortality indicated that the death rate in CHM plus CWM groups was lower than that in CWM groups (RR = 0.53, 95% CI [0.40, 0.70], *I*^2^ = 0%, *P* < 0.0001, moderate certainty). Among 11 studies, 5 were classified into “low risk” (54, 58, 67, 77, 78), 3 into “some concerns” (61, 62, 76), and 3 into “high risk” (43, 48, 53). A total of 852 participants in CHM plus CWM groups and 810 in CWM groups. A forest plot of death was shown in Figure 6C.

### Clinical symptoms recovery time

#### Fever

**Figure 7:**
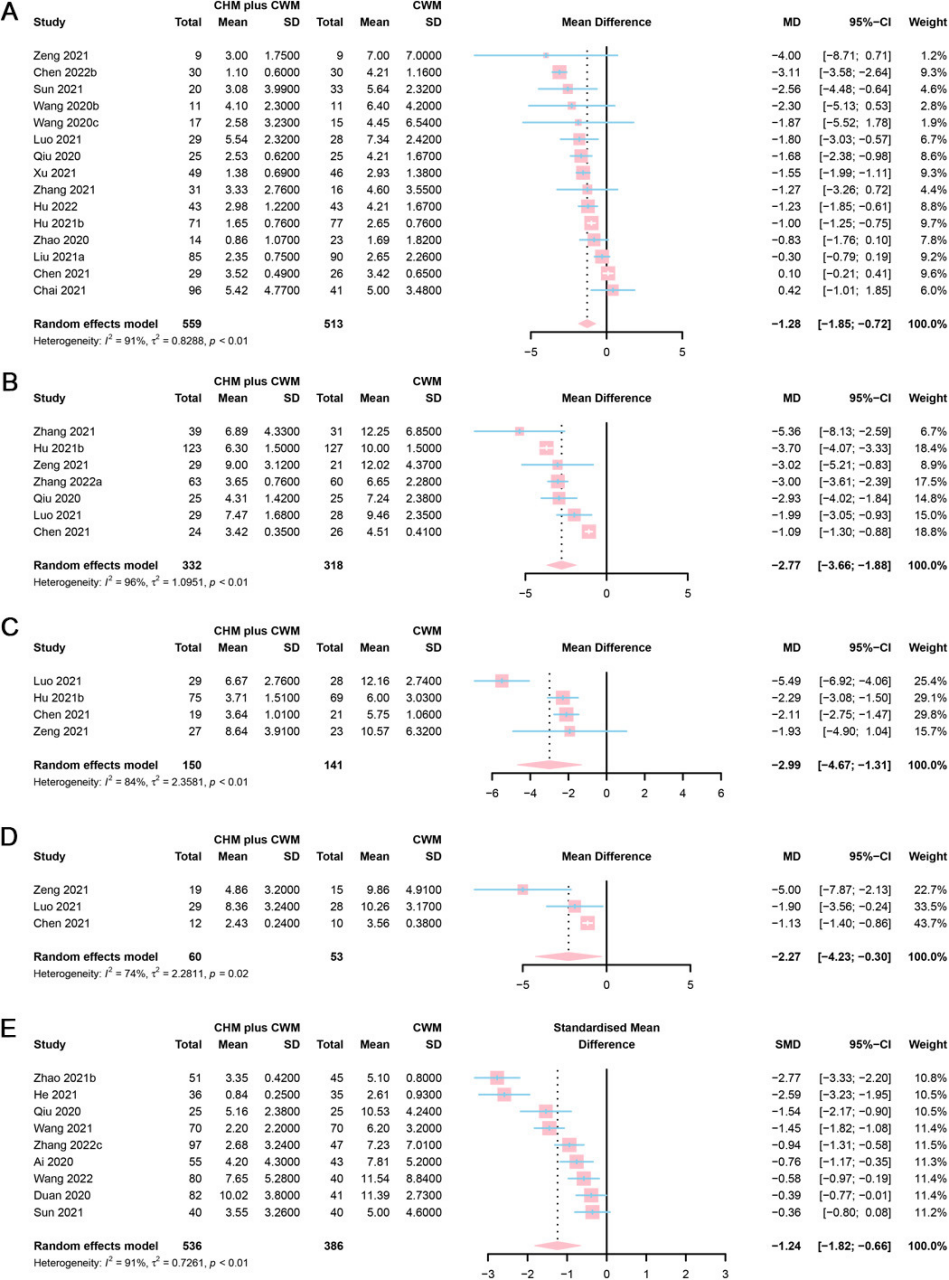
Forest plot of **(A)** fever, **(B)** cough, **(C)** fatigue, **(D)** shortness of breath recovery time, and **(E)** total score of TCM syndrome.

Sixteen studies provided fever recovery time. Six studies were assessed as “low risk” (50, 66, 77, 82–84), 9 as “some concerns” (42, 46, 48, 53, 62, 63, 68, 79, 86), and 1 as “high risk” (43). However, a study (77), only reporting the median time to fever symptom recovery of two arms (CHM plus CWM vs. CWM = 2 vs. 3 days, *P* = 0.0007), was excluded from the meta-analysis. The remaining 15 studies were included in meta-analyses. A total of 559 participants involved in CHM plus CWM groups and 513 in CWM groups. The results of meta-analysis demonstrated that fever recovery time was shorter in CHM plus CWM groups than in CWM groups (MD = −1.28, 95% CI [−1.85, −0.72], *I*^2^ = 91%, *P* < 0.0001, very low certainty) (Figure 7A).

#### Cough

Among the 8 studies focused on cough recovery time, 3 were assessed as “low risk” (50, 83, 84), 4 as “some concerns” (46, 53, 77, 86), and 1 as “high risk” (52). However, a study (52), only reporting the median time to cough symptom recovery of two arms (CHM plus CWM vs. CWM = 4 vs. 7 days, *P* < 0.05), was excluded from the meta-analysis. A total of 332 participants involved in CHM plus CWM groups and 318 in CWM groups. The results of the meta-analysis showed that cough recovery time was shorter in CHM plus CWM groups than in CWM groups (MD = −2.77, 95% CI [−3.66, −1.88], *I*^2^ = 96%, *P* < 0.0001, low certainty) (Figure 7B).

#### Fatigue

Four studies addressed fatigue recovery time. Two of them were assessed as “low risk” (50, 83), while the other 2 as “some concerns” (46, 53). As the target data in 2 studies were absent, they were excluded from the meta-analysis (50, 83). A total of 150 participants involved in CHM plus CWM groups and 141 in CWM groups. The results demonstrated that the fatigue recovery time was shorter in CHM plus CWM groups than in CWM groups (MD = −2.99, 95% CI [−4.67, −1.31], *I*^2^ = 84%, *P* = 0.0005, low certainty) (Figure 7C).

#### Shortness of breath

Three studies reported shortness of breath recovery time. One study was assessed as “low risk” (83), while the other 2 as “some concerns” (46, 53). However, 1 study was excluded for the unavailability of mean and SD values (83). A total of 60 participants involved in CHM plus CWM groups and 53 in CWM groups. The results of the meta-analysis showed that shortness of breath recovery time was shorter in CHM plus CWM groups than in CWM groups (MD = −2.27, 95% CI [−4.23, −0.30], *I*^2^ = 74%, *P* = 0.0238, low certainty) (Figure 7D).

### Total score of TCM syndrome

Ten studies included the total score of TCM syndrome. One study was assessed as “low risk” (83), 8 as “some concerns” (47, 60, 61, 66, 70, 73, 80, 86), and 1 as “high risk” (49). Different scoring systems were applied. For example, the scoring system used by Ai et al. study in the measurement of 8 primary symptoms, 3 secondary symptoms, and TCM tongue pulse, with total score ranging from 0 to 66 (49). Higher scores indicated more severe disease conditions. Meanwhile, He et al. adopted a TCM syndrome rating scale with 7 symptoms and a total score ranging from 0 to 21 (70), and Wang et al. used a rating scale with 10 symptoms and a total score ranging from 0 to 60 (73). Thus, the scores were pooled using SMD. However, 1 study was excluded as the absence of necessary data (83). A total of 536 participants involved in CHM plus CWM groups and 386 in CWM groups. The results of the meta-analysis showed that the total score of TCM syndrome of CHM plus CWM groups was lower than CWM groups (SMD = −1.24, 95% CI [−1.82, −0.66], *I*^2^ = 91%, *P* < 0.0001, very low certainty). A forest plot of total score of TCM syndrome was showed in Figure 7E.

### Laboratory indicators

In 20 studies, laboratory indicators C-reactive protein (CRP), lymphocyte (LYM), tumor necrosis factor-α (TNF-α), white blood cell (WBC), neutrophil (NEU), high sensitive C-reactive protein (hsCRP), erythrocyte sedimentation rate (ESR), and procalcitonin (PCT) were tested after treatments. Two studies were assessed as “low risk” (58, 83), 15 as “some concerns” (46, 48, 53, 61, 63, 69–71, 73, 75, 76, 79, 80, 85, 88), and 3 as “high risk” (49, 51, 56). As the required data were not obtained from articles and authors, some studies were excluded. Moreover, 1 study was excluded from the meta-analysis of CRP due to the discrepancy in data reported in figures and in texts (53). One study was excluded from the meta-analysis of PCT level, as there was a problem with the units of measurement used in it (46). The results of the meta-analysis were shown in Table 3.

**Table 3:** Results of meta-analysis of laboratory indicators.

**Laboratory indicators**	**Study**	**Sample size**	**Statistical method**	**Effect estimate (95% CI)**	***I* ^2^**	***P*-value**	**Certainty of evidence (GRADE)**
LYM count	10 [(49)^#^; (53)^†^; (56)^#^; (58)^¶^; (61)^†^; (69)^†^; (76)^†^; (79)^†^; (80); (88)^*^]	914	MD, Random	0.24 (0.12, 0.37)	92%	< 0.0001	Low
LYM percentage	3 [(73)^†^; (75)^†^; (83)^¶^]	299	MD, Random	0.19 (−3.85, 4.23)	90%	0.9265	Very low
NEU count	2 [(48)^;^ (58)^¶^]	94	MD, Random	0.90 (−1.08, 2.88)	4%	0.3726	Low
NEU percentage	2 [(75)^†^; (83)^¶^]	159	MD, Random	−2.98 (−5.93, −0.04)	0%	0.0469	Low
WBC count	12 [(48)^†^; (58)^¶^; (61)^†^; (63)^†^; (69)^†^; (71)^†^; (73)^†^; (75)^†^; (76)^†^; (79)^†^; (83)^¶^; (88)^*^]	1027	MD, Random	0.12 (−0.15, 0.39)	69%	0.3787	Very low
CRP	10 [(58)^¶^; (61)^†^; (63)^†^; (69)^†^; (73)^†^; (75)^†^; (76)^†^; (79)^†^; (80); (88)^*^]	1005	MD, Random	−3.62 (−5.05, −2.20)	74%	< 0.0001	Low
hsCRP	4 [(46)^†^; (56)^#^; (58)^¶^; (70)^†^]	224	MD, Random	−5.30 (−5.85, −4.75)	0%	< 0.0001	Moderate
PCT	3 [(58)^¶^; (76)^†^; (80)^†^]	433	MD, Random	−0.005 (−0.02, 0.01)	89%	0.4990	Very low
TNF-α	2 [(53)^†^; (79)^†^]	93	MD, Random	−7.66 (−19.11, 3.80)	93%	0.1902	Very low
ESR	5 [(56)^#^; (58)^¶^; (61)^†^; (70)^†^; (88)^*^]	327	MD, Random	−9.70 (−16.29, −3.10)	65%	0.0040	Low

* Data from the 50 mL TCM group were included in the meta-analysis;

¶ A low level of risk of bias;

† A medium level of risk of bias;

# A high level of risk of bias; LYM, lymphocyte (× 10^9^/L); NEU, neutrophil; WBC, white blood cell ( × 10^9^/L); CRP, C-reactive protein (mg/L); hsCRP, high sensitive C-reactive protein (mg/L); PCT, procalcitonin (ng/L); TNF-α, tumor necrosis factor-α (pg/mL); ESR, erythrocyte sedimentation rate (mm/H).

### Adverse reactions

**Figure 8:**
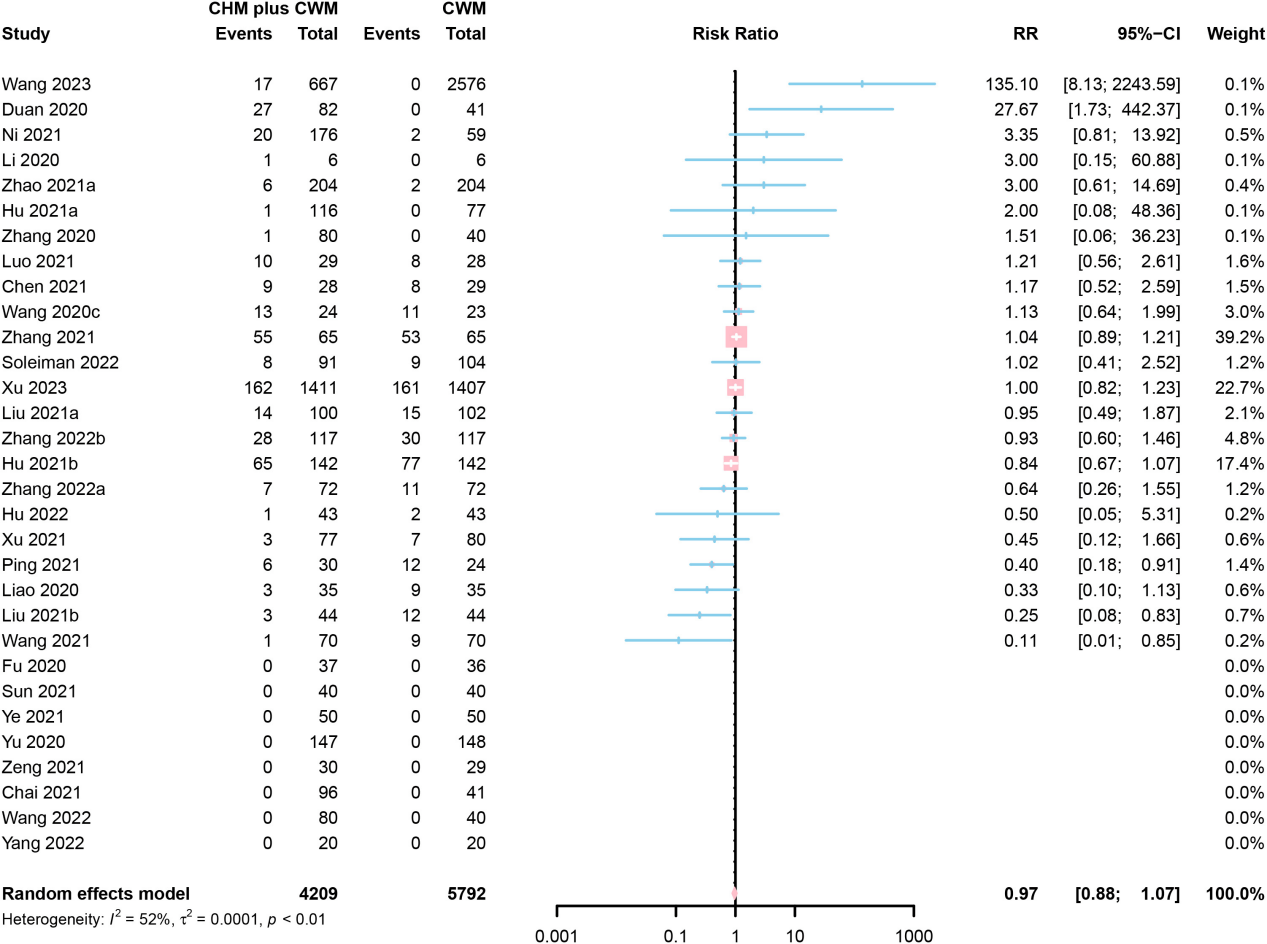
Forest plot of adverse reactions.

Of the 36 studies reporting adverse reactions, 5 were assessed as “low risk” (50, 53, 67, 83, 84), 22 as “some concerns” (46–48, 61, 62, 64, 66, 68, 69, 71–73, 82, 85, 88, 90–92), and 9 as “high risk” (43, 45, 49, 51, 52, 54, 56, 59, 65). The main adverse reactions contained diarrhea (45–48, 50, 51, 56, 59, 62, 64, 65, 67, 72, 75, 78, 82, 84, 89, 91), vomiting (46, 48, 50, 51, 56, 64, 67, 72, 75, 82, 89, 91), nausea (46, 48, 50, 51, 56, 64, 67, 72, 75, 89, 90), loss of appetite (43, 50, 51, 64, 82), abnormal liver function (43, 46, 50, 53, 62, 77, 83), itchy skin (52, 71, 75, 82, 89, 90), and rash (51, 53, 59, 67, 75, 89–91). We analyzed the differences in the incidence of adverse reactions between the two groups, involving a total of 26 types of CHM preparations (Supplementary Table S5). Commonly, the incidence of adverse reactions in CHM plus CWM treatment is equivalent to CWM group. In comparison to CWM treatment, severe/critical patients after administration of Shenhuang granule had lower risk of some adverse reactions (e.g., hypoalbuminemia, increased blood glucose, thrombocytopenia, increased total bilirubin, increased white blood cell count, abnormal serum sodium, respiratory failure or acute respiratory distress syndrome, cardiopulmonary failure, multiple organ dysfunction syndrome, total serious adverse reactions, and so on), and patients undergoing LHQW had lower incidence of diarrhea (5.6 vs. 13.4%, *P* = 0.0431); however, mild patients in JHQG group had a significantly increased risk of diarrhea (32.9 vs. 0.0%, *P* < 0.0001). Four out of 36 studies reported no adverse reactions in CHM plus CWM groups and provided no information about adverse reactions in CWM groups (49, 52, 88, 91); furthermore, the incidence of total adverse reactions is unclear in Zhou et al.'s study (50). Thus, the remaining 31 studies of adverse reactions were included in the meta-analysis. A total of 4,209 participants involved in CHM plus CWM groups and 5,792 in CWM groups. The results of the meta-analysis demonstrated no significant difference in adverse reactions rate between CHM plus CWM groups and CWM groups (RR = 0.97, 95% CI [0.88, 1.07], *I*^2^ = 52%, *P* = 0.5111, very low certainty) (Figure 8).

### Subgroup and sensitivity analysis

We further conducted subgroup analysis concerning two primary outcomes. There was no change in the pooled results in any of the subgroups with regard to clinical effective rate (all *P* < 0.05). For SARS-CoV-2 nucleic acid conversion time, the results showed no statistical difference between CHM plus CWM groups and CWM groups in the subgroup where treatment duration was not reported. The detailed results of the subgroup analysis were shown in Supplementary Figures S1–S8. We implemented sensitivity analysis by means of the leave-one-out method for the primary outcomes (Supplementary Figures S9, S10). In the meta-analysis of clinical effective rate, the *I*^2^ was 7% after removing 1 study (67), indicating this study might be the chief reason for the heterogeneity (Supplementary Figure S9).

### Publication bias

**Figure 9:**
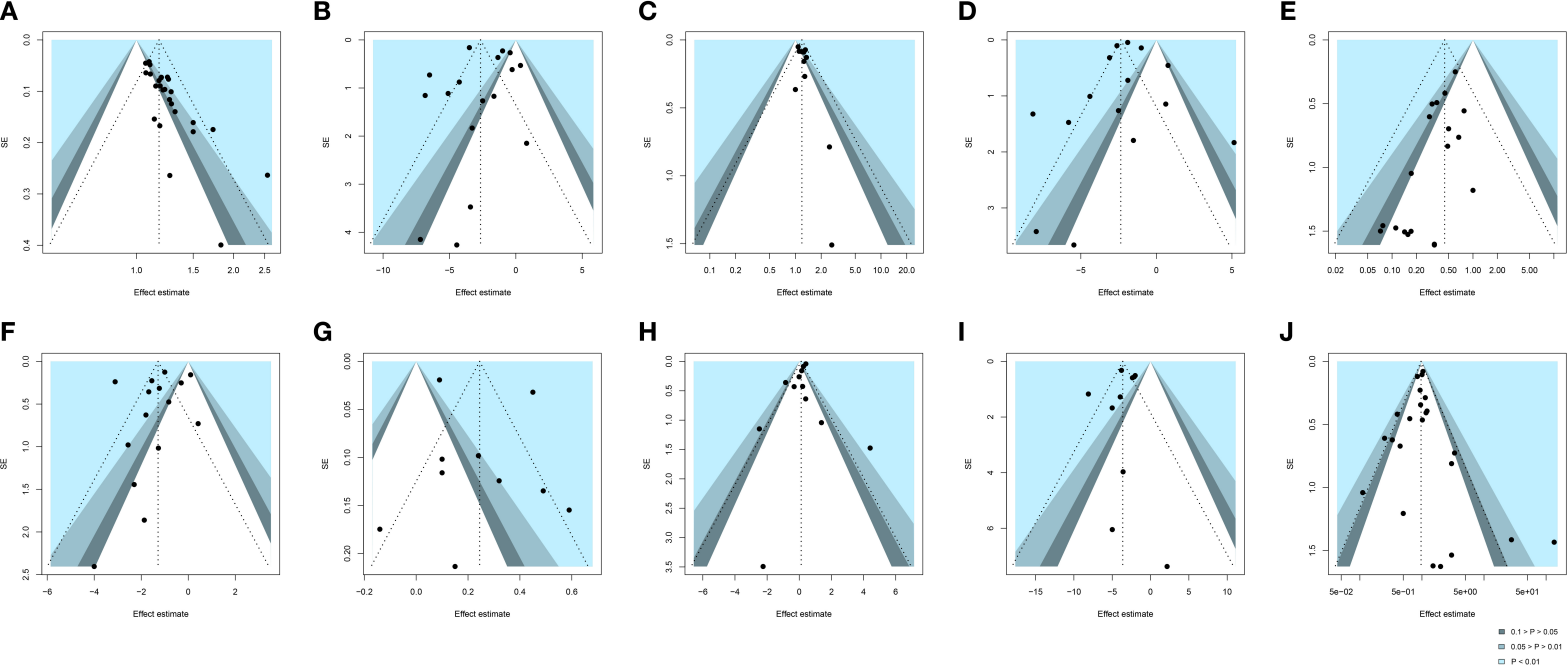
Funnel plot of **(A)** clinical effective rate, **(B)** SARS-CoV-2 nucleic acid conversion time, **(C)** chest image improvement, **(D)** duration of hospitalization, **(E)** conversion to severe cases, **(F)** fever recovery time, **(G)** LYM count, **(H)** WBC count, **(I)** CRP, and **(J)** adverse reactions. SE, standard error. Black dots mean different studies and contours mean the levels of statistical significance.

We assessed publication bias for 10 outcomes indicators (i.e., clinical effective rate, SARS-CoV-2 nucleic acid conversion time, chest image improvement, duration of hospitalization, conversion to severe cases, fever recovery time, LYM count, WBC count, CRP, and adverse reactions) which exceeded 10 studies. For clinical effective rate, an obviously asymmetric funnel plot illustrated considerable publication bias (Figure 9A). Moreover, Egger's test also supported the existence of publication bias, indicating the small-study effects (*P* < 0.0001). Eleven smaller studies were identified and trimmed with the trim-and-fill method, and the RR value after adjusting for publication bias was 1.13 (95% CI [1.10, 1.17], *I*^2^= 47%, *P* < 0.0001) (Supplementary Figure S11). For conversion to severe cases, a potential publication bias was supported by the mild asymmetry based on visual inspection of Figure 9E. Egger's test (*P* < 0.0001) also found evidence of publication bias. The results of the trim-and-fill analysis showed an insignificant change in the RR value (RR = 0.49, 95% CI [0.37, 0.64], *I*^2^ = 0%, *P* < 0.0001) (Supplementary Figure S12). Symmetric funnel plots and the Egger's test results suggested no publication bias with respect to other 8 outcomes (Figure 9).

## Discussion

CHM has been employed to combat sundry epidemic and endemic diseases for thousands of years. In the 17^th^ century, the world's first medical work on the systematic study of acute infectious diseases was published in China, in which etiology, pathogenesis, symptoms, and treatment of plague were elaborated. Likewise, Chinese traditional therapy has established many representative theories of infectious diseases (92–94). In 2003, CHM therapy was used to prevent and treat severe acute respiratory syndrome (SARS) in China (95). CHM therapy has been reported effective in improving pneumonia symptoms, quality of life, and absorption of pulmonary infiltration, while reducing clinical deterioration and decreasing corticosteroid dosage in SARS patients (96, 97). Similar to SARS-CoV, SARS-CoV-2 infection leads to an over-reaction of the immune system, causing a cytokine storm, which is closely related to the severity of COVID-19 (98). Some components of Chinese botanical drugs were reported beneficial for the suppression of excessive inflammatory responses (99). Furthermore, in 2009, CHM was nominated to treat influenza in the *Guidelines for Management of Pandemic (H1N1) 2009 Influenza*, published by the Ministry of Health of China (100). Thus, it is widely believed that CHM therapy could potentially provide an effective therapy against COVID-19. Our systematic review, including 50 RCTs, ranging from 2020 to 2023, evaluated and discussed the efficacy and safety of CHM for COVID-19.

Many CHM prescriptions were used repeatedly in these 50 RCTs, including LHQW, JYH, QFPD, JHQG, HSBD, MXSG, and so on. LHQW was the most frequently used CHM prescription, a classical prescription made from Yinqiao powder and MXSG, and contained over a dozen Chinese botanical drugs including *Lianqiao* [Forsythia suspensa (Thunb.) Vahl], *Jinyinhua* (Lonicera japonica Thunb.), *Mahuang* (Ephedra sinica Stapf.), *Kuxingren* (Prunus armeniaca L.), etc. Researches indicated LHQW can significantly inhibit SARS-CoV-2 replication, alter virus morphology, and exert anti-inflammatory activity *in vitro* (101). Wang et al. found that LHQW contains 22 key compounds which may have targets with SARS-CoV-2 by Network Pharmacology (102). We listed the chemical structures of 6 main compounds (i.e., quercetin, luteolin, kaempferol, sitosterin, naringenin, and acacetin) of LHQW in Supplementary Figure S13. Quercetin has the largest number of targets. Furthermore, it is also the chief ingredient of *Lianqiao* and *Jinyinhua* used in LHQW (102). Meanwhile, systematic reviews of clinical evidence have revealed LHQW could obviously alleviate clinical symptoms, inhibit clinical deterioration, and shorten the course of COVID-19 (25, 103, 104). We have summarized the Chinese botanical drugs that were commonly used in these 50 RCTs. *Gancao* (Glycyrrhiza uralensis Fisch. ex DC.) was mentioned most frequently. This Chinese botanical drug can be used to improve the symptoms of the respiratory tract, i.e., cough, sore throat (105). In animal models of acute pneumonia, the flavonoids in *Gancao* reduced the infiltration of neutrophils and inhibited the expression of pro-inflammatory mediators, thus exerting anti-inflammatory activity (106). Besides, glycyrrhetinic acid (Supplementary Figure S14A) in *Gancao* suppress viral replication and release in host cells (107). *Mahuang* (Ephedra sinica Stapf.) is a commonly used specie in CHM preparations. The main bioactive components contained in *Mahuang* are ephedrine and pseudoephedrine (Supplementary Figures S14B, C). *Kuxingren* (Prunus armeniaca L.) with the main active ingredient of amygdalin (Supplementary Figure S14D), also a very frequently used drug, paired with Mahuang is widely employed to combat respiratory diseases (108). This herb pair (*Mahuang-Kuxingren*) is popularly used for the treatment of bronchitis and asthma in accordance with the principle of mutual reinforcement and assistance. *Kuxingren* is defined as a poison based on TCM theory, since amygdalin is a major component of *Mahuang* but the main source of its toxicity, which can be converted to cyanide in the body and lead to fatal cyanide poisoning (109, 110); whereas the combination with *Mahuang* can prevented and antagonized the toxicity of *Kuxingren* and allow the safe use of *Kuxingren* in the clinic with few associated adverse effects (108). *Lianqiao* (Forsythia suspensa (Thunb.) Vahl) is widely used as a CHM in Asia, its main function is to clear heat and detoxify (111). Modern pharmacological studies have found that it has anti-inflammatory, antibacterial, antiviral, antioxidant, anti-tumor, neuroprotective and liver protective pharmacological effects (112). However, it is worth noting that for the treatment of COVID-19, combination formulations of multiple herbs are often used, and the possible efficacy and safety risks are often more than just the accumulation of individual herbs, due to the interaction of different herbs. The efficacy and safety of CHM are to some extent related to the dosage, proportioning, processing methods, and quality control of the herbs. In addition, standardized assessment and quality control on many CHM is indeed lacking and further research and standards are needed in the future. Furthermore, WHO reviewed three reports on Chinese herbal medicine and COVID-19 provided by Chinese experts and 12 registered and published RCTs, reached a consensus and recommended a combination of CHM and CWM therapies for the treatment of COVID-19, however, research on the specific dual mechanisms of combining CHM and CWM is lacking and this still needs to be further explored in depth in the future (113).

We found that CHM preparations were effective in treating COVID-19 without increasing the probability of adverse reactions by meta-analysis. As for the clinical effective rate, the results of subgroup and sensitivity analyses were similar to those of the meta-analysis. When a study was removed from the meta-analysis, the *I*^2^ was 7%. Thus, we thought this study was the main source of heterogeneity (67). Although the Egger's test suggested the existence of publication bias, the trim-and-fill analysis indicated that the publication bias did not affect the results of the meta-analysis. Therefore, the conclusion from the meta-analysis was considered robust. From meta-analysis results, SARS-CoV-2 nucleic acid conversion time of patients in CHM plus CWM group was 2.66 days shorter than that in CWM group. Sensitivity analysis also did not reveal a significant change when omitting any one study at a time by the leave-one-out method. However, we found no statistical difference between CHM plus CWM groups and CWM groups in the subgroup where treatment duration was not reported. Perhaps the effect depended on the duration of treatment. Additionally, the meta-analysis results indicated that CHM plus CWM could promote the improvement of chest imaging, shorten the duration of hospitalization, suppress clinical deterioration, reduce mortality, shorten the clinical symptoms (i.e., fever, cough, fatigue and shortness of breath) recovery time, reduce the TCM syndrome score, and contribute to the return of some laboratory indicators to the normal levels. Although evidence of publication bias was found for conversion to severe cases by asymmetric funnel plot and Egger's test, trim-and-fill analysis demonstrated the robustness of the pooled results. Overall, no serious adverse reactions have been identified. There was no statistically significant difference in the incidence of adverse reactions between two arms.

There are several noteworthy advantages in our systematic review. We conducted the research under the guidance of the latest international statement for systematic review and meta-analysis (31). We systematically screened the literature that assess the efficacy and safety of CHM in the context of COVID-19. The process of literature selection, data extraction, and evidence quality evaluation was conducted by following the back-to-back principle rigorously. We employed RoB2 to evaluate all the 210 outcomes included in our study, whereas most other systematic reviews only evaluated the overall quality of the original researches. We also measured the certainty of evidence through the GRADE system to determine the confidence of the effect estimates. Additionally, to ensure the high quality of the original evidence, our study only incorporated RCTs and excluded observational studies. Based on an overview that summarized the published systematic reviews regarding CHM and COVID-19, we found that the number of RCTs (50 RCTs) included in our study was more than that in other published systematic reviews (114). Compared with the previous systematic reviews, the sample size of this work is large and the data are updated, making the evidence more reliable. Importantly, another strength of our study is the thorough analysis of the possible adverse reaction produced by use of different CHM preparations. To our knowledge, this article is the first systematic review to include data on patients infected with SARS-CoV-2 Omicron variant to evaluate CHM treatment for COVID-19.

However, some limitations also exist in this study. There were inconsistent treatments with CHM in intervention groups and inconsistency in CWM treatment methods of control groups, however, we did not perform subgroup analyses according to the treatment difference. The heterogeneity among some pooled analysis studies for secondary outcomes was significant, but no subgroup and sensitivity analysis were implemented. Only electronic databases and clinical trial registers were searched. Therefore, some literature from other source that meet the inclusion criteria may have been left out. Ongoing trials and unpublished studies were not included in this work either. As COVID-19 epidemic is a sudden health event, a lack of double-blinded RCTs is widespread, and few RCTs used placebos. Allocation concealment and blind methods were not conducted in many RCTs, so the number of enrolled RCTs with low risk of bias was inadequate. The overall quality of evidence was low. The evidence certainty was mostly low or very low, which meant that differences might exist between the actual and estimated effect. To some extent, perhaps the benefits of CHM have been overstated. Additionally, a multi-center study design was scarce in this study, which could lead to some selection bias. The vast majority of RCTs are conducted in China, leading to limited representativeness of this study, which constituted a major disadvantage. We searched the ongoing RCTs concerning CHM for treating COVID-19 from trial registers. A total of 22 RCTs is being investigated in mainland China, 2 in United States, and 1 in Taiwan. These studies will be included in the next update. Further, the evidence for asymptomatic patients was absent, and we were unable to interpret the effects of CHM on such cases in a full extent.

## Conclusions

Based on current evidence, we concluded that CHM therapy was potentially an effective and safe adjunctive treatment for COVID-19. This study provided a list of CHM preparations for the treatment of COVID-19. Further, more double-blinded, multi-center, and large-sample size RCTs of high-quality are needed to perform meta-analysis yielding more accurate results.

## Data availability statement

The original contributions presented in the study are included in the article/Supplementary material, further inquiries can be directed to the corresponding author.

## Author contributions

LT conceived the study and registered the protocol. ZM and YZ searched the literature. YZ, SY, YY, JL, and JH screened and included the articles. ZM and YZ assessed the quality of included studies. SY, YY, and FW collected the data. JL and JH re-checked the data. ZM performed data-analysis, interpreted the results, and drafted the manuscript. LT, SY, FW, and ZM participated in manuscript revision. All authors re-read and re-checked the final manuscript.
