# Supplementary material for: Combination of Chinese herbal medicine and conventional western medicine for coronavirus disease 2019: a systematic review and meta-analysis
Source: Front Med (Lausanne). 2023 Jul 17;10:1175827. doi: 10.3389/fmed.2023.1175827 (PMC10387529; doi:10.3389/fmed.2023.1175827)
Supplement: Supplementary file 1 [file Data_Sheet_1.PDF]

Table S1. Search strategies of all databases and registers.

Search strategies

Embase:

('coronavirus disease 2019'/exp OR ('ncp':ti,ab OR 'novel coronavirus':ti,ab OR 'novel coronavirus pneumonia':ti,ab OR 'corona virus':ti,ab OR 'sars-cov-2':ti,ab OR 'covid-19':ti,ab OR 'coronavirus':ti,ab OR '2019-ncov':ti,ab)) AND ('random* controlled trial*':ti,ab OR 'random*':ti,ab OR 'placebo':ti,ab) AND ('chinese medicine'/exp OR ('medicine, chinese traditional':ti,ab OR 'traditional medicine, chinese':ti,ab OR 'chinese medicine, traditional':ti,ab OR 'chinese traditional medicine':ti,ab OR 'chinese medicine':ti,ab OR 'chinese herb*':ti,ab OR 'chinese plant extracts':ti,ab OR 'chinese patent medicine':ti,ab OR 'herb*':ti,ab OR 'plant extracts, chinese':ti,ab OR 'extracts, chinese plant':ti,ab))

Web of scinece:

#1 TS=(Corona virus OR COVID-19 OR 2019-nCoV OR SARS-CoV-2 OR Coronavirus OR Coronavirus disease 2019 OR Nov el Coronavirus Pneumonia OR novel coronavirus OR NCP)

#2 TS=(Medicine, Chinese Traditional OR Drugs, Chinese Herbal OR Medicine, Chinese Traditional OR Traditional Medicine, Chinese OR Chinese Medicine, Traditional OR Chinese Traditional Medicine OR Chinese Medicine OR Chinese Herb* OR Chi nese Drugs, Plant OR Herb* OR Plant Extracts, Chinese OR Chinese Plant Extracts OR Extracts, Chinese Plant OR Chinese pate nt medicine)

#3 TS=(random* controlled trial* OR random* OR placebo)

#4 TS=(#1 AND #2 AND #3)

PubMed：

((((Medicine, Chinese Traditional[MeSH Terms]) OR (Drugs, Chinese Herbal[MeSH Terms])) OR (((((((((((Medicine, Chinese Traditional[Title/Abstract]) OR (Traditional Medicine, Chinese[Title/Abstract])) OR (Chinese Medicine, Traditional[Title/Abstra ct])) OR (Chinese Traditional Medicine[Title/Abstract])) OR (Chinese Medicine[Title/Abstract])) OR (Chinese Herb*[Title/Abst ract])) OR (Chinese Drugs, Plant[Title/Abstract])) OR (Herb*[Title/Abstract])) OR (Plant Extracts, Chinese[Title/Abstract])) OR (Chinese Plant Extracts[Title/Abstract])) OR (Extracts, Chinese Plant[Title/Abstract])) OR (Chinese patent medicine[Title/Abstr act]))) AND (((Coronavirus[MeSH Terms]) OR (COVID-19[Supplementary Concept])) OR (((((((2019-nCoV[Title/Abstract]) O R (SARS-CoV-2[Title/Abstract])) OR (Corona virus[Title/Abstract])) OR (Coronavirus disease 2019[Title/Abstract])) OR (Nove l Coronavirus Pneumonia[Title/Abstract])) OR (novel coronavirus[Title/Abstract])) OR (NCP[Title/Abstract])))) AND (randomiz ed controlled trial[Publication Type] OR randomized[Title/Abstract] OR placebo[Title/Abstract])

Cochrane library:

#1 Search MeSH descriptor: [Medicine, Chinese Traditional] explode all trees

#2 Search (Medicine, Chinese Traditional):ti,ab,kw OR (Traditional Medicine,Chinese):ti,ab,kw OR (Chinese Medicine, Traditional):ti,ab,kw OR (Chinese Traditional Medicine):ti,ab,kw OR (Chinese Medicine):ti,ab,kw

#3 Search (Chinese Herb*):ti,ab,kw OR (Chinese Drugs, Plant):ti,ab,kw OR (Herb*):ti,ab,kw OR (Plant Extracts, Chinese):ti,ab,kw OR (Chinese Plant Extracts):ti,ab,kw

#4 Search (Extracts, Chinese Plant):ti,ab,kw OR (Chinese patent medicine):ti,ab,kw

#5 Search #1or#2or#3or#4

#6 Search MeSH descriptor: [Coronavirus] explode all trees

#7 Search (COVID 19):ti,ab,kw OR (2019 nCoV):ti,ab,kw OR (SARS CoV 2):ti,ab,kw OR (Corona virus):ti,ab,kw OR (Coronavirus disease 2019):ti,ab,kw

#8 Search (Novel Coronavirus Pneumonia):ti,ab,kw OR (novel coronavirus):ti,ab,kw OR

(NCP):ti,ab,kw

#9 Search #6or#7or#8

#10 Search #5and#9

CNKI：

（主题：中医（精确））OR（主题：中药（精确））OR（主题：中医药（精确））OR（主题：中成药（精确））OR（主题：中药材（精确））OR（主题：中草药（精确））OR（主题：天然药物（精确））OR（主题：自然医学（精确））OR（主题：TCM（精确））OR（主题：CHM（精确）） AND （（主题：新冠（精确））OR（主题：新型冠状病毒肺炎（精确））OR（主题：新冠肺炎（精确））OR（主题：新型冠状病毒（精确））OR（主题：冠状病毒肺炎（精确））OR（主题：2019 冠状病毒病（精确））OR（主题：NCP（精确））OR（主题：COVID-19（精确））OR（主题：2019-nCoV（精确））） AND （（摘要：随机（精确））OR（摘要：RCT（精确））OR（摘要：RCTs（精确）））

VIP：

((((((((((题名或关键词=中医 OR 题名或关键词=中药) OR 题名或关键词=中医药) OR 题名或关键词=中成药) OR 题名或关键词=中药材) OR 题名或关键词=中草药) OR 题名或关键词=天然药物) OR 题名或关键词=自然医学) OR 题名或关键词=TCM) OR 题名或关键词=CHM) AND ((((((((题名或关键词=新冠 OR 题名或关键词=新型冠状病毒肺炎) OR 题名或关键词=新冠肺炎) OR 题名或关键词=新型冠状病毒) OR 题名或关键词=冠状病毒肺炎) OR 题名或关键词=2019 冠状病毒病) OR 题名或关键词=NCP) OR (题名或关键词=COVID AND (NOT 题名或关键词=19))) OR (题名或关键词=2019 AND (NOT 题名或关键词=nCoV)))) AND ((摘要=随机 OR 摘要=RCT) OR 摘要=RCTs))

CBM：

((随机) OR ("随机对照试验"[不加权:扩展])) AND (("中草药"[不加权:扩展]) OR ("中成药"[不加权:扩展]) OR (CHM) OR (TCM) OR (自然医学) OR (天然药物) OR (中药材) OR (中药) OR (中医)) AND ((2019-nCoV) OR (COVID-19) OR (NCP) OR (2019 冠状病毒病) OR (冠状病毒肺炎) OR (新型冠状病毒) OR (新冠肺炎) OR (新型冠状病毒肺炎) OR (新冠))

Wanfang:

(主题:(中医 or 中药 or 中医药 or 中成药 or 中药材 or 中草药 or 天然药物 or 自然医学 or TCM or CHM) and 主题:(新冠 or 新型冠状病毒肺炎 or 新冠肺炎 or 新型冠状病毒 or 冠状病毒肺炎 or 2019 冠状病毒病 or NCP or COVID-19 or 2019-nCoV) and 摘要:(随机 or RCT or RCTs))

ChiCTR:

1.注册题目：COVID-19 或者 新型冠状病毒肺炎； 2.干预措施：中药 或者 中医 或者 中医药； 3.公开试验结果文件：是；4.上传试验结果文件：是

ClinicalTrials.gov:

1. Condition or disease: COVID-19; 2. Other terms: Chinese medicine OR Chinese herbal medicine; 3. Study types: All Studies; 4. Study Results: Studies With Results; 5. Search: Condition or disease AND Other terms AND Study types AND Study Results

WHO ICTRP:

1. Title: coronavirus disease 2019 OR covid-19 OR sars-cov-2

2. Condition: coronavirus disease 2019 OR ncp OR novel coronavirus OR novel coronavirus pneumonia OR corona virus OR sars-cov-2 OR covid-19 OR coronavirus OR 2019-ncov

3. Intervention: chinese medicine OR medicine, chinese traditional OR traditional medicine, chinese OR chinese medicine, traditional OR chinese traditional medicine OR chinese medicine OR chinese plant extracts OR chinese patent medicine OR herb*

CNKI = Chinese National knowledge Infrastructure Database, VIP = Chinese Science and Technology Journals Database, CBM = Chinese Biomedical Literature Database, ChiCTR = Chinese Clinical Trial Registration Center, WHO ICTRP = WHO International Clinical Trials Registry platform.

Table S2. Summary of funding sources for the included studies.

Study	Funding source
Ping 2021	Pneumonia Emergency Research Project of the Prevention and Treatment of COVID-19 with Traditional Chinese Medicine (2020J003).
Qiu 2020	Traditional Chinese Medicine Special Project for COVID-19 Emergency of National Administration of Traditional Chinese Medicine (2020ZYLCYJ02-2); National Chinese Medicine Innovation Talent Training Program.
Sun 2020	NA
Sun 2021	Clinical Study on Traditional Chinese Medicine Treatment of COVID-19 of Science and Technology Commission of Shanghai Municipality (20411950200002); Shanghai Infectious Diseases TCM Prevention and Treatment Capacity Training Project (ZYYB-NLPY-09, ZYYB-NLPY-15); Shanghai Key Clinical Specialty Project (shslczdzk05101); Shanghai Key Laboratory of Clinical Chinese Medicine (20DZ2272200); Shanghai "Medical Garden Rising Star" Youth Medical Talent Training Funding Program in 2020; Chinese Medicine Talent Program of Shanghai University of Traditional Chinese Medicine in 2020.
Ai 2020	Project of Administration of Traditional Chinese Medicine of Guangdong Province of China (No.2020ZYYJ01).
Chen 2021	NA
Duan 2020	National Key R&D Program "Public Security Risk Prevention and Control and Emergency Technology and Equipment" Project (2020YFC0841600); Hubei Provincial Department of Science and Technology Novel Coronavirus Pneumonia Prevention and Control Emergency Special Project (2020FCA027).
Fu 2020	National "Thirteenth Five-Year Plan" Major Special Project (2018ZX10101001); Guangdong Province Novel Coronavirus Pneumonia Prevention and Control Emergency Special Project (2020B111115001); Scientific Research Project of Administration of Traditional Chinese Medicine of Guangdong Province of China (20201271).
He 2021	NA
Hu 2021a	NA
Hu 2021b	National Key Research and Development Plan for the Emergency Management of Novel Coronavirus Pneumonia (2020YFC0845100); Guangdong Administration of Traditional Chinese Medicine Management for the Emergency Management of Novel Coronavirus Pneumonia with Traditional Chinese Medicine (2020ZYYJ05); Beijing Municipal Science and Technology Commission NCP Emergency Project; Hebei Provincial Department of Science and Technology NCP Prevention and Control Emergency Scientific Research Project (20277708D); The Science Research Project of the Guangdong Province (2020B111110001); The Foundation of Macau University of Science and Technology (2020A0042).
Liao2020	NA
Liu 2021a	National Key Research and Development Plan for the Emergency Management of Novel Coronavirus Pneumonia (2020YFC0841500).
Liu 2021b	NA
Luo 2021	No funding was received for the conduct of this study.
Xu 2021	National Natural Science Foundation of China (62041701); Special Project of Ministry of Science and Technology of China (2020YFC0841600); National Major Scientific and Technological Project (2017ZX10305501).
Yang 2021	Jining Municipal National Key Research and Development Program (2020JKNS015).
Ye 2020	COVID-19 Project, Dongzhimen Hospital, Beijing University of Chinese Medicine (2020-dzmyy-lczx-yj001); Ten-Thousand Talents Program (W02020052).
Ye 2021	Internal Grants from the People's Hospital Affiliated to Fujian University of Traditional Chinese Medicine, "Research and Product Development of Novel Coronavirus Pneumonia Prevention and Treatment Methods" (XG202011).
Yu 2020	NA
Zeng 2021	Wenzhou Municipal Science and Technology Bureau (CN) (ZY202003).
Zhang 2020	NA
Zhang 2021	Key Projects of Jiangxi Province (2020YBBGW0008).
Zhao 2020	National Natural Science Fund (81903994).
Zheng 2020	NA
Zhou 2021	Emergency Committee of the World Federation of Chinese Medicine Societies and Shanghai Society of Traditional Chinese Medicine; Novel Coronavirus Pneumonia Emergency Tackling Key Project (SJZLJZ.N01); National Key Research and Development Program of China (2018YFC1705900).
Ni 2021	National Key R&D Program of China (2020YFC0841400); Tongji Hospital Clinical Research Project (XXGZBDYJ009, 2019YBKY019).
Wang 2020c	National Administration of Traditional Chinese Medicine Project (2020ZYLCYJ05-1); National Natural Science Foundation of China (81630100, 81721002, 81930110); China PLA Biosecurity Project (No. 19SWAQ13); China PLA Emergency Project (BWS20J006); Beijing Administration of Traditional Chinese Medicine Project (YJ2020-03, SYFY202011).
An 2021	New Coronavirus Infection Pneumonia Chinese Medicine Emergency Project (2020ZYLCYJ04-1,3,4); Traditional Chinese Medicine Special Project for COVID-19 Emergency of National Administration of Traditional Chinese Medicine (2020ZYLCYJ04-1).
Li 2020	National Key Research and Development Program of Shanxi Province of China (201603D321105).
Tan 2021	NA

Wang 2020a	National Natural Science Foundation of China (81771349); Project of Shaanxi Provincial Administration of Traditional Chinese Medicine (2020-YJ012).
Wang 2020b	Health Commission of Shijianzhuang 2020 Shijiazhuang Municipal Special Scientific Research Fund Projects (201460513A -3); Shijiazhuang Science and Technology Bureau Novel Coronavirus Pneumonia Prevention and Control Emergency Project.
Wang 2021	NA
Wen 2020	Hunan Province Innovation-Type Novel Coronavirus Pneumonia Prevention and Control Emergency Special Project (2020SK3014); Natural Science Foundation of Hunan Province (2018JJ2452); Medical and Health Research Project of Hunan Province (B2017209).
Xiao 2020	Special Project for Emergency of the Ministry of Science and Technology (2020YFC0845000); Traditional Chinese Medicine Special Project for COVID-19 Emergency of National Administration of Traditional Chinese Medicine (2020ZYLCYJ04-1, 2020ZYLCYJ04-3).
Xiong 2020	Wuhan Municipal Health Commission (2020100).
Zhang 2022	Key Research and Development Plan of Hebei Province (20372504D); the Special Project of Ministry of Science and Technology of China (2020YFC0841600); National Major Scientific and Technological Project (2017ZX10305501).
Zhao 2021a	National Key R&D Program of China (2020YFC0841500).
Chai 2021	Key Research and Development Emergency Projects of Science Technology Department of Zhejiang Province (2020C03127).
Yang 2022	National Natural Science Foundation of China (81771349); Shaanxi Administration of Traditional Chinese Medicine (2020-YJ012).
Zhao 2021b	Jinzhou Municipal Special Funds of the National Natural Science Foundation (2020CB21-02).
Soleiman 2022	AJA University of Medical Sciences (97001131).
Wang 2023	Shanghai University of Chinese Medicine Emergency Response Project for Omicron COVID-19 in 2022 (2022YJ-07) and National Natural Science Foundation of China (81973102).
Zhang 2020b	National Natural Science Foundation of China (82104783).
Chen 2022b	Projects to be established by Hubei Provincial Department of Science and Technology on new pneumonia emergency science and technology research.
Hu 2022	NA
Wang 2022	Special funding project for the prevention and control of the new crown epidemic in general universities of the Guangdong Provincial Education Department (2020KZDZX1053).
Xu 2023	NA
Zhang 2022d	Ministry of Science and Technology “Thirteenth Five-Year Plan” Major New Drug Creation Project (2017ZX09304002); State Administration of Traditional Chinese Medicine 2022 National Famous Old Chinese Medicine Experts Inheritance Studio Construction Project; Shanghai University of Traditional Chinese Medicine 2022 Annual Emergency Research and Research Project for Responding to Pneumonia Infected with Novel Coronavirus by Omicron (2022YJ-21).

Table S3. Sources and components of CHM used in the included studies.

Study	Chinese Herbal Medicine (CHM)	Sources	Components	Quality control reported? (Y/N)	Chemical analysis reported? (Y/N)
Ping 2021	Jiawei Yupingfeng powder	NA	**Jiawei Yupingfeng powder:** Astragalus mongholicus Bunge [Fabaceae; Astragali radix] 30 g, Atractylodes macrocephala Koidz. [Asteraceae; Atractylodis macrocephalae rhizoma] 12 g, Saposhnikovia divaricata (Turcz. ex Ledeb.) Schischk. [Apiaceae; Saposhnikoviae radix] 10 g, Smilax glabra Roxb. [Smilacaceae; Smilacis glabrae rhizoma] 10 g, Atractylodes lancea (Thunb.) DC. [Asteraceae; Atractylodis rhizoma] 10 g, Pogostemon cablin (Blanco) Benth. [Lamiaceae; Pogostemonis herba] 10 g, Perilla frutescens (L.) Britton [Lamiaceae; Perillae caulis] 10 g, Pinellia ternata (Thunb.) Makino [Araceae; Pinelliae rhizoma praeparatum] 8 g, Wurfbainia villosa (Lour.) Skornick. & A.D.Poulsen [Zingiberaceae; Amomi fructus] 6 g, Zingiber officinale Roscoe [Zingiberaceae; Zingiberis rhizoma recens] 6 g.	N	N
Qiu 2020	Maxing Xuanfei Jiedu Decoction	Chongqing Traditional Chinese Medicine Hospital	**Maxing Xuanfei Jiedu Decoction:** Ephedra sinica Stapf [Ephedraceae; Ephedrae herba praeparata cum melle] 9 g, Prunus armeniaca L. [Rosaceae; Armeniacae semen amarum] 12 g, Gypsum Fibrosum 15~30 g, Fritillaria thunbergii Miq. [Liliaceae; Fritillariae thunbergii bulbus] 12 g, Cryptotympana pustulata Fabricius [Cicadidae; Cicadae periostracum] 10 g, body of sick Bombyx mori Linnaeus [Bombycidae; Bombyx batryticatus] 15 g, Curcuma longa L. [Zingiberaceae; Curcumae longae rhizoma] 12 g, Platycodon grandiflorus (Jacq.) A.DC. [Campanulaceae; Platycodonis radix] 12 g, Citrus × aurantium L. [Rutaceae; Aurantii fructus] 12 g, Amomum tsao-ko Crevost and Lemarié [Zingiberaceae; Tsaoko fructus] 9 g, Amomurn kravanh Pierre ex Gagnep. [Zingiberaceae; Amomi fructus rotundus] 12 g.	N	N
Sun 2020	Lianhua Qingke granule	Shijiazhuang Yiling Pharmaceutical	**Lianhua Qingke granule:** Ephedra sinica Stapf [Ephedraceae; Ephedrae herba praeparata cum melle], Forsythia suspensa (Thunb.) Vahl [Oleaceae; Forsythiae fructus], Morus alba L. [Moraceae; Mori cortex], Prunus armeniaca L. [Rosaceae; Armeniacae semen amarum], Lonicera confusa DC. [Caprifoliaceae; Lonicerae flos], Rheum palmatum L. [Polygonaceae; Rhei radix et rhizoma].	N	N
Sun 2021	Liushen pills	Shanghai Leiyunshang Pharmaceutical	**Liushen pills:** Abelmoschus moschatus Medik. [Malvaceae; Moschus], Bufonis venenum, bovis calculus, Senecio bonariensis Hook. & Arn. [Asteraceae; Margarita] et al.	N	N
Ai 2020	Pneumonia No. 1 formula	NA	**Pneumonia No. 1 formula:** Artemisia annua L. [Asteraceae; Artemisiae annuae herba] 10 g, Astragalus mongholicus Bunge [Fabaceae; Astragali radix] 45 g, Cremastra appendiculata (D.Don) Makino [Orchidaceae; Cremastrae pseudobulbus pleiones pseudobulbus] 20 g, Forsythia suspensa (Thunb.) Vahl [Oleaceae; Forsythiae fructus] 30 g, Scutellaria baicalensis Georgi [Lamiaceae; Scutellariae radix] 10 g, Lonicera japonica Thunb. [Caprifoliaceae; Lonicerae japonicae flos]15 g, Isatis tinctoria L. [Brassicaceae; Isatidis folium] 10 g, Bupleurum chinense DC. [Apiaceae; Bupleuri radix] 5 g, Cryptotympana pustulata Fabricius [Cicadidae; Cicadae periostracum] 10 g, Kitagawia praeruptora (Dunn) Pimenov [Apiaceae; Peucedani radix] 5 g, Fritillaria cirrhosa D.Don [Liliaceae; Fritillariae cirrhosae bulbus] 10 g, Fritillaria thunbergii Miq. [Liliaceae; Fritillariae thunbergii bulbus]10 g, Prunus mume (Siebold) Siebold and Zucc. [Rosaceae; Mume fructus] 30 g, Scrophularia ningpoensis Hemsl. [Scrophulariaceae; Scrophulariae radix] 10 g, Poria cocos (Schw.) Wolf [Polyporaceae; Poria] 30 g,	N	N

			Pseudostellaria heterophylla (Miq.) Pax [Caryophyllaceae; Pseudostellariae radix] 15 g.		
Chen 2021	Lianhua Qingwen capsule	Shijiazhuang Yiling Pharmaceutical	**Lianhuaqingwen capsule:** Forsythia suspensa (Thunb.) Vahl [Oleaceae; Forsythiae fructus], Lonicera japonica Thunb. [Caprifoliaceae; Lonicerae japonicae flos], Ephedra sinica Stapf [Ephedraceae; Ephedrae herba praeparata cum melle], Prunus armeniaca L. [Rosaceae; Armeniacae semen amarum], Gypsum Fibrosum, Isatis tinctoria L. [Brassicaceae; Isatidis radix], Dryopteris crassirhizoma Nakai [Polypodiaceae; Dryopteridis crassirhizomatis rhizoma], Houttuynia cordata Thunb. [Saururaceae; Houttuyniae herba], Pogostemon cablin (Blanco) Benth. [Lamiaceae; Pogostemonis herba], Rheum palmatum L. [Polygonaceae; Rhei radix et rhizoma], Rhodiola crenulata (Hook.f. and Thomson) H.Ohba [Crassulaceae; Rhodiolae crenulatae radix et rhizoma], l-menthol, Glycyrrhiza uralensis Fisch. ex DC. [Fabaceae; Glycyrrhizae radix et rhizoma].	N	N
Duan 2020	Jinhua Qinggan granule	Juxechang (Beijing) Pharmaceutical	**Jinhua Qinggan granule:** Lonicera japonica Thunb. [Caprifoliaceae; Lonicerae japonicae flos], Gypsum Fibrosum, Ephedra sinica Stapf [Ephedraceae; Ephedrae herba praeparata cum melle], Prunus armeniaca L. [Rosaceae; Armeniacae semen amarum], Scutellaria baicalensis Georgi [Lamiaceae; Scutellariae radix], Forsythia suspensa (Thunb.) Vahl [Oleaceae; Forsythiae fructus], Fritillaria thunbergii Miq. [Liliaceae; Fritillariae thunbergii bulbus], Anemarrhena asphodeloides Bunge [Asparagaceae; Anemarrhenae rhizoma], Arctium lappa L. [Asteraceae; Arctii fructus], Artemisia annua L. [Asteraceae; Artemisiae annuae herba], Mentha canadensis L. [Lamiaceae; Menthae haplocalycis herba], Glycyrrhiza uralensis Fisch. ex DC. [Fabaceae; Glycyrrhizae radix et rhizoma].	N	N
Fu 2020	Toujie Quwen granule	Guangdong E-fong Pharmaceutical	**Toujiequwen granule:** Forsythia suspensa (Thunb.) Vahl [Oleaceae; Forsythiae fructus]30 g, Cremastra appendiculata (D.Don) Makino [Orchidaceae; Cremastrae pseudobulbus pleiones pseudobulbus] 20 g, Lonicera japonica Thunb. [Caprifoliaceae; Lonicerae japonicae flos]15 g, Scutellaria baicalensis Georgi [Lamiaceae; Scutellariae radix] 10 g, Isatis tinctoria L. [Brassicaceae; Isatidis folium] 10 g, Bupleurum chinense DC. [Apiaceae; Bupleuri radix]5 g, Artemisia annua L. [Asteraceae; Artemisiae annuae herba] 10 g, Cryptotympana pustulata Fabricius [Cicadidae; Cicadae periostracum] 10 g, Kitagawia praeruptora (Dunn) Pimenov [Apiaceae; Peucedani radix]5 g, Fritillaria cirrhosa D.Don [Liliaceae; Fritillariae cirrhosae bulbus] 10 g, Fritillaria thunbergii Miq. [Liliaceae; Fritillariae thunbergii bulbus] 10 g, Prunus mume (Siebold) Siebold and Zucc. [Rosaceae; Mume fructus] 30 g, Scrophularia ningpoensis Hemsl. [Scrophulariaceae; Scrophulariae radix] 10 g, Astragalus mongholicus Bunge [Fabaceae; Astragali radix] 45 g, Poria cocos (Schw.) Wolf [Polyporaceae; Poria] 30 g, Pseudostellaria heterophylla (Miq.) Pax [Caryophyllaceae; Pseudostellariae radix] 15 g.	N	N
He 2021	Buzhong Yiqi decoction	NA	**Buzhong yiqi decoction:** Astragalus mongholicus Bunge [Fabaceae; Astragali radix] 10 g, Panax ginseng C.A.Mey. [Araliaceae; Ginseng Radix et Rhizoma] 3 g, Glycyrrhiza uralensis Fisch. ex DC. [Fabaceae; Glycyrrhizae radix et rhizoma praeparata cum melle]5 g, Atractylodes macrocephala Koidz. [Asteraceae; Atractylodis macrocephalae rhizoma] 3 g, Citrus reticulata Blanco [Rutaceae; Citri reticulatae pericarpium] 3 g, Angelica sinensis (Oliv.) Diels [Apiaceae; Angelicae sinensis radix] 3 g, Actaea racemosa L. [Ranunculaceae; Cimicifugae rhizoma] 3 g, Bupleurum chinense DC. [Apiaceae; Bupleuri radix] 3 g.	N	N
Hu 2021a	Jinyinhua oral liquid	Hubei Zhenao Jinyinhua Pharmaceutical	**Jinyinhua oral liquid:** Lonicera japonica Thunb. [Caprifoliaceae; Lonicerae japonicae flos].	N	N
Hu 2021b	Lianhua Qingwen capsule	Shijiazhuang Yiling Pharmaceutical	**Lianhuaqingwen capsule:** Forsythia suspensa (Thunb.) Vahl [Oleaceae; Forsythiae fructus], Lonicera japonica Thunb. [Caprifoliaceae; Lonicerae japonicae flos], Ephedra sinica Stapf [Ephedraceae;	Y	Y

			Ephedrae herba praeparata cum melle], Isatis tinctoria L. [Brassicaceae; Isatidis radix], Pogostemon cablin (Blanco) Benth. [Lamiaceae; Pogostemonis herba], Rheum palmatum L. [Polygonaceae; Rhei radix et rhizoma], Glycyrrhiza uralensis Fisch. ex DC. [Fabaceae; Glycyrrhizae radix et rhizoma], Dryopteris crassirhizoma Nakai [Polypodiaceae; Dryopteridis crassirhizomatis rhizoma], Rhodiola crenulata (Hook.f. and Thomson) H.Ohba [Crassulaceae; Rhodiolae crenulatae radix et rhizoma], Houttuynia cordata Thunb. [Saururaceae; Houttuyniae herba], Prunus armeniaca L. [Rosaceae; Armeniacae semen amarum], Gypsum Fibrosum, l-menthol.		
Liao 2020	CHM decoction	NA	**CHM decoction:** Prunus armeniaca L. [Rosaceae; Armeniacae semen amarum] 10 g, Gypsum Fibrosum 30 g, Rheum palmatum L. [Polygonaceae; Rhei radix et rhizoma] 6 g, Prunus persica (L.) Batsch [Rosaceae; Persicae semen] 10 g, Atractylodes lancea (Thunb.) DC. [Asteraceae; Atractylodis rhizoma] 10 g, Glycyrrhiza uralensis Fisch. ex DC. [Fabaceae; Glycyrrhizae radix et rhizoma] 4 g.	N	N
Liu 2021a	Huashi Baidu granule	NA	**Huashibaidu granule:** Ephedra sinica Stapf [Ephedraceae; Ephedrae herba praeparata cum melle], Prunus armeniaca L. [Rosaceae; Armeniacae semen amarum], Gypsum Fibrosum, Glycyrrhiza uralensis Fisch. ex DC. [Fabaceae; Glycyrrhizae radix et rhizoma], Pogostemon cablin (Blanco) Benth. [Lamiaceae; Pogostemonis herba], Magnolia officinalis Rehder and E.H.Wilson [Magnoliaceae; Magnoliae officinalis cortex], Atractylodes macrocephala Koidz. [Asteraceae; Atractylodis macrocephalae rhizoma], Amomum tsao-ko Crevost and Lemarié [Zingiberaceae; Tsaoko fructus], Pinellia ternata (Thunb.) Makino [Araceae; Pinelliae rhizoma praeparatum], Poria cocos (Schw.) Wolf [Polyporaceae; Poria], Rheum palmatum L. [Polygonaceae; Rhei radix et rhizoma].	Y	N
Liu 2021b	Lianhua Qingwen capsule	Shijiazhuang Yiling Pharmaceutical	**Lianhuaqingwen capsule:** Lonicera japonica Thunb. [Caprifoliaceae; Lonicerae japonicae flos], Forsythia suspensa (Thunb.) Vahl [Oleaceae; Forsythiae fructus], Houttuynia cordata Thunb. [Saururaceae; Houttuyniae herba], Rheum palmatum L. [Polygonaceae; Rhei radix et rhizoma], Glycyrrhiza uralensis Fisch. ex DC. [Fabaceae; Glycyrrhizae radix et rhizoma], Gypsum Fibrosum, Prunus armeniaca L. [Rosaceae; Armeniacae semen amarum], Ephedra sinica Stapf [Ephedraceae; Ephedrae herba praeparata cum melle], Isatis tinctoria L. [Brassicaceae; Isatidis radix], l-menthol, Rhodiola crenulata (Hook.f. and Thomson) H.Ohba [Crassulaceae; Rhodiolae crenulatae radix et rhizoma].	N	N
	Pneumonia No. 2 formula	NA	**Pneumonia No. 2 formula:** Prunus armeniaca L. [Rosaceae; Armeniacae semen amarum], Ephedra sinica Stapf [Ephedraceae; Ephedrae herba praeparata cum melle], Ginkgo biloba L. [Ginkgoaceae; Ginkgo semen], Pheretima aspergillum (E.Perrier) [Megascolecidae; Pheretima], Descurainia sophia (L.) Webb ex Prantl [Brassicaceae; Descurainiae semen], Schisandra chinensis (Turcz.) Baill. [Schisandraceae; Schisandrae chinensis fructus], Pinellia ternata (Thunb.) Makino [Araceae; Pinelliae rhizoma], Glycyrrhiza uralensis Fisch. ex DC. [Fabaceae; Glycyrrhizae radix et rhizoma], Perilla frutescens (L.) Britton [Lamiaceae; Perillae fructus], Morus alba L. [Moraceae; Mori cortex], Tussilago farfara L. [Asteraceae; Farfarae flos].		
Luo 2021	Xuebijing injection	Tianjin Chase Sun Pharmaceutical	**Xuebijing injection:** Paeonia lactiflora Pall. [Paeoniaceae; Paeoniae radix rubra], Angelica sinensis (Oliv.) Diels [Apiaceae; Angelicae sinensis radix], Ligusticum striatum DC. [Apiaceae; Chuanxiong rhizoma], Carthamus tinctorius L. [Asteraceae; Carthami flos], Salvia miltiorrhiza Bunge [Lamiaceae; Salviae miltiorrhizae radix et rhizoma].	N	N
Xu 2021	Reduning injection	NA	**Reduning injection:** Artemisia annua L. [Asteraceae; Artemisiae annuae herba], Lonicera japonica Thunb. [Caprifoliaceae; Lonicerae japonicae flos], Gardenia jasminoides J.Ellis [Rubiaceae; gardeniae	N	N

			fructus].		
Yang 2021	CHM decoction	NA	**CHM decoction for cold-dampness syndrome:** Poria cocos (Schw.) Wolf [Polyporaceae; Poria] 30 g, Astragalus mongholicus Bunge [Fabaceae; Astragali radix] 30 g, Forsythia suspensa (Thunb.) Vahl [Oleaceae; Forsythiae fructus]15 g, Codonopsis pilosula (Franch.) Nannf. [Campanulaceae; Codonopsis radix] 15 g, Atractylodes macrocephala Koidz. [Asteraceae; Atractylodis macrocephalae rhizoma] 15 g, Lonicera japonica Thunb. [Caprifoliaceae; Lonicerae japonicae flos]15 g, Phragmites australis (Cav.) Trin. ex Steud. [Poaceae; Phragmitis rhizoma] 24 g, Saposhnikovia divaricata (Turcz. ex Ledeb.) Schischk. [Apiaceae; Saposhnikoviae radix] 12 g, Rhodiola crenulata (Hook.f. and Thomson) H.Ohba [Crassulaceae; Rhodiolae crenulatae radix et rhizoma] 9 g, Amomum tsao-ko Crevost and Lemarié [Zingiberaceae; Tsaoko fructus] 6 g, Pogostemon cablin (Blanco) Benth. [Lamiaceae; Pogostemonis herba] 9 g.<br>**CHM decoction for damp-heat syndrome:** Poria cocos (Schw.) Wolf [Polyporaceae; Poria] 30 g, Astragalus mongholicus Bunge [Fabaceae; Astragali radix] 30 g, Forsythia suspensa (Thunb.) Vahl [Oleaceae; Forsythiae fructus]15 g, Codonopsis pilosula (Franch.) Nannf. [Campanulaceae; Codonopsis radix] 15 g, Atractylodes macrocephala Koidz. [Asteraceae; Atractylodis macrocephalae rhizoma] 15 g, Lonicera japonica Thunb. [Caprifoliaceae; Lonicerae japonicae flos]15 g, Phragmites australis (Cav.) Trin. ex Steud. [Poaceae; Phragmitis rhizoma] 24 g, Saposhnikovia divaricata (Turcz. ex Ledeb.) Schischk. [Apiaceae; Saposhnikoviae radix] 12 g, Rhodiola crenulata (Hook.f. and Thomson) H.Ohba [Crassulaceae; Rhodiolae crenulatae radix et rhizoma] 9 g, Coix lacryma- jobi var. ma-yuen (Rom.Caill.) Stapf [Poaceae; Coicis semen] 24 g, Scutellaria baicalensis Georgi [Lamiaceae; Scutellariae radix] 9 g.	N	N
Ye 2020	CHM decoction	Jiangyin Tianjiang Pharmaceutical	**Modified maxingshigan formula:** Prunus armeniaca L. [Rosaceae; Armeniacae semen amarum] 10 g, Gypsum Fibrosum 30 g, Trichosanthes kirilowii Maxim. [Cucurbitaceae; Trichosanthis fructus] 30 g, Rheum palmatum L. [Polygonaceae; Rhei radix et rhizoma] 6 g, Ephedra sinica Stapf [Ephedraceae; Ephedrae herba] 6 g, Ephedra sinica Stapf [Ephedraceae; Ephedrae herba praeparata cum melle] 6 g, Descurainia sophia (L.) Webb ex Prantl [Brassicaceae; Descurainiae semen]10 g, Prunus persica (L.) Batsch [Rosaceae; Persicae semen] 10 g; Amomum tsao-ko Crevost and Lemarié [Zingiberaceae; Tsaoko fructus] 6 g, Areca catechu L. [Arecaceae; Arecae semen] 10 g, Atractylodes lancea (Thunb.) DC. [Asteraceae; Atractylodis rhizoma] 10 g.<br>**Modified Shengfutang formula:** Panax ginseng C.A.Mey. [Araliaceae; Ginseng Radix et Rhizoma] 15 g, Aconitum carmichaelii Debeaux [Ranunculaceae; Aconiti lateralis radix praeparata] 10 g , Cornus officinalis Siebold and Zucc. [Cornaceae; Corni fructus] 15 g.	Y	N
	Chinese patent medicine	NA	**Chinese patent medicine:** Suhexianwan or Angongniuhuangwan.		
Ye 2021	Modified Shengjiang Powder	The Affiliated People's Hospital of Fujian University of Traditional Chinese Medicine	**Modified Shengjiang Powder:** body of sick Bombyx mori Linnaeus [Bombycidae; Bombyx batryticatus] 10 g, Cryptotympana pustulata Fabricius [Cicadidae; Cicadae periostracum] 4 g, Curcuma longa L. [Zingiberaceae; Curcumae longae rhizoma] 8 g, Rheum palmatum L. [Polygonaceae; Rhei radix et rhizoma] 4 g, Astragalus mongholicus Bunge [Fabaceae; Astragali radix] 30 g, Atractylodes macrocephala Koidz. [Asteraceae; Atractylodis macrocephalae rhizoma] 15 g, Saposhnikovia divaricata (Turcz. ex Ledeb.) Schischk. [Apiaceae; Saposhnikoviae radix] 9 g, Isatis tinctoria L. [Brassicaceae; Isatidis folium] 15 g, Houttuynia cordata Thunb. [Saururaceae; Houttuyniae herba] 15 g, Pogostemon cablin (Blanco) Benth. [Lamiaceae; Pogostemonis herba] 9 g.	N	N
Yu 2020	Lianhua Qingwen Granules	Beijing Yiling	**Lianhua Qingwen Granules:** Forsythia suspensa (Thunb.) Vahl [Oleaceae; Forsythiae fructus],	N	N

		Pharmaceutical	Lonicera japonica Thunb. [Caprifoliaceae; Lonicerae japonicae flos], Ephedra sinica Stapf [Ephedraceae; Ephedrae herba praeparata cum melle], Prunus armeniaca L. [Rosaceae; Armeniacae semen amarum], Gypsum Fibrosum, Isatis tinctoria L. [Brassicaceae; Isatidis radix], Dryopteris crassirhizoma Nakai [Polypodiaceae; Dryopteridis crassirhizomatis rhizoma], Houttuynia cordata Thunb. [Saururaceae; Houttuyniae herba], Pogostemon cablin (Blanco) Benth. [Lamiaceae; Pogostemonis herba], Rheum palmatum L. [Polygonaceae; Rhei radix et rhizoma], Rhodiola crenulata (Hook.f. and Thomson) H.Ohba [Crassulaceae; Rhodiolae crenulatae radix et rhizoma], l-menthol, Glycyrrhiza uralensis Fisch. ex DC. [Fabaceae; Glycyrrhizae radix et rhizoma].		
Zeng 2021	Maxingshigan-Weijing decoction	NA	**Maxingshigan-Weijing decoction:** Ephedra sinica Stapf [Ephedraceae; Ephedrae herba praeparata cum melle] 10 g, Prunus armeniaca L. [Rosaceae; Armeniacae semen amarum] 10 g, Gypsum Fibrosum 45 g, Coptis chinensis Franch. [Ranunculaceae; Coptidis rhizoma] 30 g, Prunus persica (L.) Batsch [Rosaceae; Persicae semen] 20 g, Trichosanthes kirilowii Maxim. [Cucurbitaceae; Trichosanthis radix] 20 g, Citrus reticulata Blanco [Rutaceae; Citri reticulatae pericarpium] 12 g, Makino [Araceae; Pinelliae rhizoma praeparatum cum zingibere et alumine] 12 g, Bambusa tuldoides Munro [Poaceae; Bambusae caulis in taenias] 12 g, Descurainia sophia (L.) Webb ex Prantl [Brassicaceae; Descurainiae semen] 30 g, Acorus calamus var. angustatus Besser [Acoraceae; acori tatarinowii rhizoma] 15 g, Curcuma longa L. [Zingiberaceae; Curcumae rhizoma] 10 g, Glycyrrhiza uralensis Fisch. ex DC. [Fabaceae; Glycyrrhizae radix et rhizoma] 5 g.	N	N
Zhang 2020	Jinyinhua Oral Liquid	Zhenao jinyinhua pharmaceutical	**Jinyinhua Oral Liquid:** Lonicera japonica Thunb. [Caprifoliaceae; Lonicerae japonicae flos]et al.	N	N
Zhang 2021	Xiyanping injection	Jiangxi Qingfeng Pharmaceutical	**Xiyanping injection:** Andrographis paniculata (Burm.f.) Nees [Acanthaceae; Andrographis herba] et al.	N	Y
Zhao 2020	Yidu-toxicity blocking lung decoction	Guangdong E-fong Pharmaceutical	**Yidu-toxicity blocking lung decoction:** Prunus armeniaca L. [Rosaceae; Armeniacae semen amarum] 10 g, Gypsum Fibrosum 30 g, Trichosanthes kirilowii Maxim. [Cucurbitaceae; Trichosanthis fructus] 30 g, Rheum palmatum L. [Polygonaceae; Rhei radix et rhizoma] 6 g, Ephedra sinica Stapf [Ephedraceae; Ephedrae herba] 6 g, Ephedra sinica Stapf [Ephedraceae; Ephedrae herba praeparata cum melle] 6 g, Descurainia sophia (L.) Webb ex Prantl [Brassicaceae; Descurainiae semen] 10 g, Prunus persica (L.) Batsch [Rosaceae; Persicae semen] 10 g, Amomum tsao-ko Crevost and Lemarié [Zingiberaceae; Tsaoko fructus] 6 g, Areca catechu L. [Arecaceae; Arecae semen] 10 g, Atractylodes lancea (Thunb.) DC. [Asteraceae; Atractylodis rhizoma] 10 g.	N	N
Zheng 2020	Xiaochaihu decoction with Maxing Shigan decoction	NA	**Xiaochaihu decoction with Maxing Shigan decoction:** Bupleurum chinense DC. [Apiaceae; Bupleuri radix] 20 g, Scutellaria baicalensis Georgi [Lamiaceae; Scutellariae radix] 12 g, Pinellia ternata (Thunb.) Makino [Araceae; Pinelliae rhizoma praeparatum] 12 g, Codonopsis pilosula (Franch.) Nannf. [Campanulaceae; Codonopsis radix] 15 g, Zingiber officinale Roscoe [Zingiberaceae; Zingiberis rhizoma] 10 g, Ziziphus jujuba Mill. [Rhamnaceae; Jujubae fructus] 12 g, Glycyrrhiza uralensis Fisch. ex DC. [Fabaceae; Glycyrrhizae radix et rhizoma praeparata cum melle] 10 g, Ephedra sinica Stapf [Ephedraceae; Ephedrae herba praeparata cum melle] 10 g, Prunus armeniaca L. [Rosaceae; Armeniacae semen amarum] 12 g, Gypsum Fibrosum 30 g, Phragmites australis (Cav.) Trin. ex Steud. [Poaceae; Phragmitis rhizoma] 30 g, Aster tataricus L.f. [Asteraceae; Asteris radix et rhizoma] 15 g, Tussilago farfara L. [Asteraceae; Farfarae flos] 15 g, Cryptotympana pustulata Fabricius [Cicadidae; Cicadae periostracum] 10 g, Coix lacryma- jobi var. ma-yuen (Rom.Caill.) Stapf [Poaceae; Coicis semen] 20 g,	N	N

			Hordeum vulgare L. [Poaceae; Hordei fructus germinatus] 20 g.		
	Sanren decoction	NA	**Sanren decoction:** Prunus armeniaca L. [Rosaceae; Armeniacae semen amarum] 10 g, Amomurn kravanh Pierre ex Gagnep. [Zingiberaceae; Amomi fructus rotundus] 10 g, Coix lacryma- jobi var. ma-yuen (Rom.Caill.) Stapf [Poaceae; Coicis semen] 30 g, Magnolia officinalis Rehder and E.H.Wilson [Magnoliaceae; Magnoliae officinalis cortex] 10 g, Pinellia ternata (Thunb.) Makino [Araceae; Pinelliae rhizoma praeparatum] 10 g, Tetrapanax papyrifer (Hook.) K.Koch [Araliaceae; Tetrapanacis medulla] 10 g, Glycyrrhiza uralensis Fisch. ex DC. [Fabaceae; Glycyrrhizae radix et rhizoma] 10 g, Talci pulvis10 g, Anemarrhena asphodeloides Bunge [Asparagaceae; Anemarrhenae rhizoma] 10 g, Scutellaria baicalensis Georgi [Lamiaceae; Scutellariae radix] 10 g, Ephedra sinica Stapf [Ephedraceae; Ephedrae herba praeparata cum melle] 8 g, Poria cocos (Schw.) Wolf [Polyporaceae; Poria] 10 g, Bupleurum chinense DC. [Apiaceae; Bupleuri radix] 15 g, Lophatherum gracile Brongn. [Poaceae; Lophatheri herba] 10 g.		
Zhou 2021	Shenhuang Granule	Beijing Tcmages Pharmaceutical	**Shenhuang Granule:** Panax ginseng C.A.Mey. [Araliaceae; Ginseng Radix et Rhizoma] 50 g, Rheum palmatum L. [Polygonaceae; Rhei radix et rhizoma] 40 g, Sargentodoxa cuneata (Oliv.) Rehder & E.H.Wilson [Lardizabalaceae; Sargentodoxae caulis] 30 g, Taraxacum sect. Taraxacum F.H.Wigg. [Asteraceae;Taraxaci herba] 30 g, Aconitum carmichaelii Debeaux [Ranunculaceae; Aconiti lateralis radix praeparata] 50 g, Hirudo 6 g.	N	N
Ni 2021	Shuanghuanglian oral liquid	Sanchine Pharmaceutical, Harbin Pharmaceutical Group	**Shuanghuanglian oral liquid:** Lonicera japonica Thunb. [Caprifoliaceae; Lonicerae japonicae flos], Scutellaria baicalensis Georgi [Lamiaceae; Scutellariae radix], Forsythia suspensa (Thunb.) Vahl [Oleaceae; Forsythiae fructus].	N	N
Wang 2020c	Keguan-1	Beijing Tcmages Pharmaceutical	**Keguan-1:** Lonicera japonica Thunb. [Caprifoliaceae; Lonicerae japonicae flos]30 g, Forsythia suspensa (Thunb.) Vahl [Oleaceae; Forsythiae fructus] 30 g, Morus alba L. [Moraceae; Mori folium] 15 g, Chrysanthemum × morifolium (Ramat.) Hemsl. [Asteraceae; Chrysanthemi flos] 10 g, Coix lacryma-jobi var. ma-yuen (Rom.Caill.) Stapf [Poaceae; Coicis semen] 30 g, Fritillaria thunbergii Miq. [Liliaceae; Fritillariae thunbergii bulbus] 15 g, Prunus armeniaca L. [Rosaceae; Armeniacae semen amarum] 9 g.	Y	Y
An 2021	Jinhua Qinggan granules	Juxiechang (Beijing) Pharmaceutical	**Jinhua Qinggan granules:** Lonicera japonica Thunb. [Caprifoliaceae; Lonicerae japonicae flos], Gypsum Fibrosum, Ephedra sinica Stapf [Ephedraceae; Ephedrae herba praeparata cum melle], Prunus armeniaca L. [Rosaceae; Armeniacae semen amarum], Scutellaria baicalensis Georgi [Lamiaceae; Scutellariae radix], Forsythia suspensa (Thunb.) Vahl [Oleaceae; Forsythiae fructus], Fritillaria thunbergii Miq. [Liliaceae; Fritillariae thunbergii bulbus], Anemarrhena asphodeloides Bunge [Asparagaceae; Anemarrhenae rhizoma], Arctium lappa L. [Asteraceae; Arctii fructus], Artemisia annua L. [Asteraceae; Artemisiae annuae herba], Mentha canadensis L. [Lamiaceae; Menthae haplocalycis herba], Glycyrrhiza uralensis Fisch. ex DC. [Fabaceae; Glycyrrhizae radix et rhizoma].	Y	N
Li 2020	Qingfei Paidu decoction	NA	**Qingfei Paidu decoction:** Ephedra sinica Stapf [Ephedraceae; Ephedrae herba praeparata cum melle] 9 g, Glycyrrhiza uralensis Fisch. ex DC. [Fabaceae; Glycyrrhizae radix et rhizoma praeparata cum melle] 6 g, Prunus armeniaca L. [Rosaceae; Armeniacae semen amarum] 9 g, Gypsum Fibrosum 15~30 g, Cinnamomum cassia (L.) J.Presl. [Lauraceae; Cinnamomi ramulus] 9 g, Alisma plantago-aquatica L. [Alismataceae; Alismatis rhizoma] 9 g, Polyporus umbellatus (Pers.) Fries [Polyporaceae; Polyporus] 9 g, Atractylodes macrocephala Koidz. [Asteraceae; Atractylodis macrocephalae rhizoma] 9 g, Poria cocos (Schw.) Wolf [Polyporaceae; Poria] 15 g, Bupleurum chinense DC. [Apiaceae; Bupleuri radix] 16 g, Scutellaria baicalensis Georgi [Lamiaceae; Scutellariae radix] 6 g, Makino [Araceae; Pinelliae rhizoma praeparatum cum zingibere et alumine] 9 g, Zingiber officinale Roscoe [Zingiberaceae; Zingiberis	N	N

			rhizoma recens] 9 g, Aster tataricus L.f. [Asteraceae; Asteris radix et rhizoma] 9 g, Tussilago farfara L. [Asteraceae; Farfarae flos] 9 g, Iris domestica (L.) Goldblatt and Mabb. [Iridaceae; Belamcandae rhizoma] 9 g, Asarum sieboldii Miq. [Aristolochiaceae; Asari radix et rhizoma] 6 g, Dioscorea oppositifolia L. [Dioscoreaceae; Dioscoreae rhizoma] 12 g, Citrus × aurantium L. [Rutaceae; Aurantii fructus immaturus] 6 g, Citrus reticulata Blanco [Rutaceae; Citri reticulatae pericarpium] 6 g, Pogostemon cablin (Blanco) Benth. [Lamiaceae; Pogostemonis herba] 9 g.		
Tan 2021	Lianhua Qingwen capsule	Beijing Yiling Pharmaceutical	**Lianhua Qingwen capsule:** Forsythia suspensa (Thunb.) Vahl [Oleaceae; Forsythiae fructus], Lonicera japonica Thunb. [Caprifoliaceae; Lonicerae japonicae flos], Ephedra sinica Stapf [Ephedraceae; Ephedrae herba praeparata cum melle], Prunus armeniaca L. [Rosaceae; Armeniacae semen amarum], Gypsum Fibrosum, Isatis tinctoria L. [Brassicaceae; Isatidis radix], Dryopteris crassirhizoma Nakai [Polypodiaceae; Dryopteridis crassirhizomatis rhizoma], Houttuynia cordata Thunb. [Saururaceae; Houttuyniae herba], Pogostemon cablin (Blanco) Benth. [Lamiaceae; Pogostemonis herba], Rheum palmatum L. [Polygonaceae; Rhei radix et rhizoma], Rhodiola crenulata (Hook.f. and Thomson) H.Ohba [Crassulaceae; Rhodiolae crenulatae radix et rhizoma], l-menthol, Glycyrrhiza uralensis Fisch. ex DC. [Fabaceae; Glycyrrhizae radix et rhizoma] .	N	N
Wang 2020a	Buzhong Yiqi formula	NA	**Buzhong Yiqi formula:** Astragalus mongholicus Bunge [Fabaceae; Astragali radix] 30 g, Panax ginseng C.A.Mey. [Araliaceae; Ginseng Radix et Rhizoma] 15 g, Glycyrrhiza uralensis Fisch. ex DC. [Fabaceae; Glycyrrhizae radix et rhizoma] 15 g, Atractylodes macrocephala Koidz. [Asteraceae; Atractylodis macrocephalae rhizoma] 10 g, Citrus reticulata Blanco [Rutaceae; Citri reticulatae pericarpium] 6 g, Angelica sinensis (Oliv.) Diels [Apiaceae; Angelicae sinensis radix] 10 g, Ziziphus jujuba Mill. [Rhamnaceae; Jujubae fructus] 6, Zingiber officinale Roscoe [Zingiberaceae; Zingiberis rhizoma recens] 9 slices, Bupleurum chinense DC. [Apiaceae; Bupleuri radix] 12 g, Actaea racemosa L. [Ranunculaceae; Cimicifugae rhizoma] 6 g.	N	N
	Huhuang Paidu formula	NA	**Huhuang Paidu formula:** Coptis chinensis Franch. [Ranunculaceae; Coptidis rhizoma] 20 g, Rheum palmatum L. [Polygonaceae; Rhei radix et rhizoma] 10 g, Scutellaria baicalensis Georgi [Lamiaceae; Scutellariae radix] 10 g, Atractylodes lancea (Thunb.) DC. [Asteraceae; Atractylodis rhizoma] 10 g, Aster tataricus L.f. [Asteraceae; Asteris radix et rhizoma] 10 g, Houttuynia cordata Thunb. [Saururaceae; Houttuyniae herba] 10 g, Taraxacum sect. Taraxacum F.H.Wigg. [Asteraceae;Taraxaci herba] 10 g, Reynoutria japonica Houtt. [Polygonaceae; Polygoni cuspidati rhizoma et radix] 10 g, Astragalus mongholicus Bunge [Fabaceae; Astragali radix] 20 g.		
	Baimu Qingre Jiedu formula	NA	**Baimu Qingre Jiedu formula:** Pueraria montana var. lobata (Willd.) Maesen & S.M.Almeida ex Sanjappa & Predeep [Fabaceae; Puerariae lobatae radix] 15 g, Angelica dahurica (Hoffm.) Benth. & Hook.f. ex Franch. & Sav. [Apiaceae;Angelicae dahuricae radix] 12 g, Magnolia biondii Pamp. [Magnoliaceae; Magnoliae flos] 9 g, Isatis tinctoria L. [Brassicaceae; Isatidis radix] 30 g, Forsythia suspensa (Thunb.) Vahl [Oleaceae; Forsythiae fructus] 15 g, Fritillaria thunbergii Miq. [Liliaceae; Fritillariae thunbergii bulbus] 12 g.		
	CHM inhalation formula	NA	**CHM inhalation formula:** Coptis chinensis Franch. [Ranunculaceae; Coptidis rhizoma] 20 g, Rheum palmatum L. [Polygonaceae; Rhei radix et rhizoma] 10 g, Scutellaria baicalensis Georgi [Lamiaceae; Scutellariae radix] 10 g, Atractylodes lancea (Thunb.) DC. [Asteraceae; Atractylodis rhizoma] 10 g, Aster tataricus L.f. [Asteraceae; Asteris radix et rhizoma] 10 g, Houttuynia cordata Thunb. [Saururaceae; Houttuyniae herba] 10 g, Taraxacum sect. Taraxacum F.H.Wigg. [Asteraceae;Taraxaci herba] 10 g,		

			Reynoutria japonica Houtt. [Polygonaceae; Polygoni cuspidati rhizoma et radix] 10 g, Astragalus mongholicus Bunge [Fabaceae; Astragali radix] 20 g.		
Wang 2020b	Qingre Kangdu oral liquid<br>Lanxiang Jiedu oral liquid	The Fifth Hospital of Shijiazhuang City	NA	N	N
Wang 2021	Qingfei Paidu Decoction	Hefei China Resources Sanjiu, Pharmaceutical	**Qingfei Paidu Decoction:** Glycyrrhiza uralensis Fisch. ex DC. [Fabaceae; Glycyrrhizae radix et rhizoma praeparata cum melle] 6 g, Ephedra sinica Stapf [Ephedraceae; Ephedrae herba praeparata cum melle] 9 g, Gypsum Fibrosum 15-30 g, Prunus armeniaca L. [Rosaceae; Armeniacae semen amarum] 9 g, Polyporus umbellatus (Pers.) Fries [Polyporaceae; Polyporus] 9 g, Cinnamomum cassia (L.) J.Presl. [Lauraceae; Cinnamomi ramulus] 9 g, Atractylodes macrocephala Koidz. [Asteraceae; Atractylodis macrocephalae rhizoma] 9 g, Alisma plantago-aquatica L. [Alismataceae; Alismatis rhizoma] 9 g, Bupleurum chinense DC. [Apiaceae; Bupleuri radix] 16 g, Poria cocos (Schw.) Wolf [Polyporaceae; Poria] 15 g, Scutellaria baicalensis Georgi [Lamiaceae; Scutellariae radix] 6 g, Iris domestica (L.) Goldblatt and Mabb. [Iridaceae; Belamcandae rhizoma] 9 g, Makino [Araceae; Pinelliae rhizoma praeparatum cum zingibere et alumine] 9 g, Aster tataricus L.f. [Asteraceae; Asteris radix et rhizoma] 9 g, Zingiber officinale Roscoe [Zingiberaceae; Zingiberis rhizoma recens] 9 g, Pogostemon cablin (Blanco) Benth. [Lamiaceae; Pogostemonis herba] 9 g, Citrus × aurantium L. [Rutaceae; Aurantii fructus immaturus] 6 g, Citrus reticulata Blanco [Rutaceae; Citri reticulatae pericarpium] 6 g, Asarum sieboldii Miq. [Aristolochiaceae; Asari radix et rhizoma] 6 g, Dioscorea oppositifolia L. [Dioscoreaceae; Dioscoreae rhizoma] 12 g, Tussilago farfara L. [Asteraceae; Farfarae flos] 9 g.	N	N
Wen 2020	Xuebijing injection	NA	**Xuebijing injection:** Carthamus tinctorius L. [Asteraceae; Carthami flos], Paeonia lactiflora Pall. [Paeoniaceae; Paeoniae radix rubra], Ligusticum striatum DC. [Apiaceae; Chuanxiong rhizoma], Salvia miltiorrhiza Bunge [Lamiaceae; Salviae miltiorrhizae radix et rhizoma], Angelica sinensis (Oliv.) Diels [Apiaceae; Angelicae sinensis radix].	N	N
Xiao 2020	Huoxiang Zhengqi dropping pills	Tianjin Tasly Pharmaceutical Group	**Huoxiang Zhengqi dropping pills:** Pogostemon cablin (Blanco) Benth. [Lamiaceae; Pogostemonis herba], Atractylodes lancea (Thunb.) DC. [Asteraceae; Atractylodis rhizoma], Citrus reticulata Blanco [Rutaceae; Citri reticulatae pericarpium], Areca catechu L. [Arecaceae; Arecae semen], Glycyrrhiza uralensis Fisch. ex DC. [Fabaceae; Glycyrrhizae radix et rhizoma], Poria cocos (Schw.) Wolf [Polyporaceae; Poria], Magnolia officinalis Rehder and E.H.Wilson [Magnoliaceae; Magnoliae officinalis cortex], Pinellia ternata (Thunb.) Makino [Araceae; Pinelliae rhizoma], Perilla frutescens (L.) Britton [Lamiaceae, Perillae folium], Angelica dahurica (Hoffm.) Benth. & Hook.f. ex Franch. & Sav. [Apiaceae;Angelicae dahuricae radix].	Y	N
	Lianhua Qingwen granules	Beijing Yiling Pharmaceutical	**Lianhua Qingwen granules:** Forsythia suspensa (Thunb.) Vahl [Oleaceae; Forsythiae fructus], Ephedra sinica Stapf [Ephedraceae; Ephedrae herba praeparata cum melle], Lonicera japonica Thunb. [Caprifoliaceae; Lonicerae japonicae flos], Gypsum Fibrosum, Isatis tinctoria L. [Brassicaceae; Isatidis radix], Mentha canadensis L. [Lamiaceae; Menthae haplocalycis herba], Dryopteris crassirhizoma Nakai [Polypodiaceae; Dryopteris crassirhizomatis rhizoma], Rhodiola crenulata (Hook.f. and Thomson) H.Ohba [Crassulaceae; Rhodiolae crenulatae radix et rhizoma], Pogostemon cablin (Blanco) Benth. [Lamiaceae; Pogostemonis herba], Rheum palmatum L. [Polygonaceae; Rhei radix et rhizoma], Houttuynia cordata Thunb. [Saururaceae; Houttuyniae herba], Glycyrrhiza uralensis Fisch. ex DC. [Fabaceae; Glycyrrhizae radix et rhizoma].		
Xiong 2020	Xuanfei Baidu decoction	NA	**Xuanfei Baidu decoction:** Ephedra sinica Stapf [Ephedraceae; Ephedrae herba praeparata cum melle] 8	N	N

			g, Prunus armeniaca L. [Rosaceae; Armeniacae semen amarum] 15 g, Gypsum Fibrosum 30 g, Atractylodes lancea (Thunb.) DC. [Asteraceae; Atractylodis rhizoma] 10 g, Coix lacryma- jobi var. ma-yuen (Rom.Caill.) Stapf [Poaceae; Coicis semen] 30 g, Pogostemon cablin (Blanco) Benth. [Lamiaceae; Pogostemonis herba] 15 g, Reynoutria japonica Houtt. [Polygonaceae; Polygoni cuspidati rhizoma et radix] 20 g, Descurainia sophia (L.) Webb ex Prantl [Brassicaceae; Descurainiae semen] 15 g, Verbena officinalis L. [Verbenaceae; Verbenae herba] 30 g, Phragmites australis (Cav.) Trin. ex Steud. [Poaceae; Phragmitis rhizoma] 30 g, Artemisia annua L. [Asteraceae; Artemisiae annuae herba] 25 g, Citrus reticulata Blanco [Rutaceae; Citri exocarpium rubrum] 20 g, Glycyrrhiza uralensis Fisch. ex DC. [Fabaceae; Glycyrrhizae radix et rhizoma] 10 g.		
Zhang 2022	Lianhua Qingke Tablets	Shijiazhuang Yiling Pharmaceutical	NA	N	N
Zhao 2021a	Huashibaidu granule	Beijing Huayi Pharmaceutical	**Huashibaidu granule:** Ephedra sinica Stapf [Ephedraceae; Ephedrae herba praeparata cum melle] 6 g, Prunus armeniaca L. [Rosaceae; Armeniacae semen amarum] 9 g, Gypsum Fibrosum 15 g, Glycyrrhiza uralensis Fisch. ex DC. [Fabaceae; Glycyrrhizae radix et rhizoma] 3g, Pogostemon cablin (Blanco) Benth. [Lamiaceae; Pogostemonis herba] 10 g, Magnolia officinalis Rehder & E.H.Wilson [Magnoliaceae; Magnoliae officinalis cortex] (Houpu) 10 g, Atractylodes lancea (Thunb.) DC. [Asteraceae; Atractylodis rhizoma] 15 g, Amomum tsao-ko Crevost and Lemarié [Zingiberaceae; Tsaoko fructus] 10 g, Pinellia ternata (Thunb.) Makino [Araceae; Pinelliae rhizoma praeparatum] 9 g, Poria cocos (Schw.) Wolf [Polyporaceae; Poria] 15 g, Rheum palmatum L. [Polygonaceae; Rhei radix et rhizoma] 5 g, Astragalus mongholicus Bunge [Fabaceae; Astragali radix] 10 g, Descurainia sophia (L.) Webb ex Prantl [Brassicaceae; Descurainiae semen] 10 g, Paeonia lactiflora Pall. [Paeoniaceae; Paeoniae radix rubra] 10 g.	Y	N
Chai 2021	Pneumonia No. 1 formula	NA	**Pneumonia No. 1 formula:** Tetrastigma hemsleyanum Diels & Gilg [Vitaceae; Tetrastigma hemsleyanum diels et gilg] 20 g, Nepeta tenuifolia Benth. [Lamiaceae; Schizonepetae herba] 12 g, Saposhnikovia divaricata (Turcz. ex Ledeb.) Schischk. [Apiaceae; Saposhnikoviae radix] 9 g, Forsythia suspensa (Thunb.) Vahl [Oleaceae; Forsythiae fructus] 18 g, Lonicera japonica Thunb. [Caprifoliaceae; Lonicerae japonicae flos]9 g, Kitagawia praeruptora (Dunn) Pimenov [Apiaceae; Peucedani radix] 9 g, Pogostemon cablin (Blanco) Benth. [Lamiaceae; Pogostemonis herba] 9 g, Atractylodes lancea (Thunb.) DC. [Asteraceae; Atractylodis rhizoma] 12 g, Phragmites australis (Cav.) Trin. ex Steud. [Poaceae; Phragmitis rhizoma] 30 g, Glycyrrhiza uralensis Fisch. ex DC. [Fabaceae; Glycyrrhizae radix et rhizoma] 3 g.	N	N
	Pneumonia No. 2 formula	NA	**Pneumonia No. 2 formula:** Rheum palmatum L. [Polygonaceae; Rhei radix et rhizoma] 9 g, Gypsum Fibrosum 30 g, Natrii sulfas 9 g, bubali cornu 30 g, Atractylodes lancea (Thunb.) DC. [Asteraceae; Atractylodis rhizoma] 15 g, Scrophularia ningpoensis Hemsl. [Scrophulariaceae; Scrophulariae radix] 15 g, Curcuma longa L. [Zingiberaceae; curcumae radix] 15 g, Fritillaria thunbergii Miq. [Liliaceae; Fritillariae thunbergii bulbus] 15 g, Descurainia sophia (L.) Webb ex Prantl [Brassicaceae; Descurainiae semen] 20 g, Paeonia × suffruticosa Andrews [Paeoniaceae; Moutan cortex] 15 g, Paeonia lactiflora Pall. [Paeoniaceae; Paeoniae radix rubra] 15 g, Panax ginseng C.A.Mey. [Araliaceae; Ginseng Radix et Rhizoma] 6 g, Glycyrrhiza uralensis Fisch. ex DC. [Fabaceae; Glycyrrhizae radix et rhizoma] 9 g.		
	Pneumonia No. 3 formula	NA	**Pneumonia No. 3 formula:** Astragalus mongholicus Bunge [Fabaceae; Astragali radix] 30g, Pseudostellaria heterophylla (Miq.) Pax [Caryophyllaceae; Pseudostellariae radix] 15		

			g, Adenophora stricta Miq. [Campanulaceae; Adenophorae radix] 15 g, Poria cocos (Schw.) Wolf [Polyporaceae; Poria] 15 g, Atractylodes macrocephala Koidz. [Asteraceae; Atractylodis macrocephalae rhizoma] 12 g, Ophiopogon japonicus (Thunb.) Ker Gawl. [Asparagaceae; Ophiopogonis radix] 12 g, Schisandra chinensis (Turcz.) Baill. [Schisandraceae; Schisandrae chinensis fructus] 6 g, Wurfbainia villosa (Lour.) Skornick. & A.D.Poulsen [Zingiberaceae; Amomi fructus] 5 g, Glycyrrhiza uralensis Fisch. ex DC. [Fabaceae; Glycyrrhizae radix et rhizoma praeparata cum melle] 6 g.		
Yang 2022	Qi-nourishing essence-replenishing decoction	Xi'an International Medical Center Hospital Pharmacy	**Qi-nourishing essence-replenishing decoction:** Astragalus mongholicus Bunge [Fabaceae; Astragali radix] 30 g, Panax ginseng C.A.Mey. [Araliaceae; Ginseng Radix et Rhizoma] 15 g, Glycyrrhiza uralensis Fisch. ex DC. [Fabaceae; Glycyrrhizae radix et rhizoma]15 g, Atractylodes macrocephala Koidz. [Asteraceae; Atractylodis macrocephalae rhizoma] 10 g, Citrus reticulata Blanco [Rutaceae; Citri reticulatae pericarpium] 6 g, Angelica sinensis (Oliv.) Diels [Apiaceae; Angelicae sinensis radix] 10 g, Ziziphus jujuba Mill. [Rhamnaceae; Jujubae fructus] 6 objects, Zingiber officinale Roscoe [Zingiberaceae; Zingiberis rhizoma recens] 9 pieces, Bupleurum chinense DC. [Apiaceae; Bupleuri radix] 12 g, Actaea racemosa L. [Ranunculaceae; Cimicifugae rhizoma] 6 g.	N	N
	Hu-Huang decoction	Xi'an International Medical Center Hospital Pharmacy	**Hu-Huang decoction:** Coptis chinensis Franch. [Ranunculaceae; Coptidis rhizome] 20 g, Rheum palmatum L. [Polygonaceae; Rhei radix et rhizoma] 10 g, Scutellaria baicalensis Georgi [Lamiaceae; Scutellariae radix] 10 g, Atractylodes lancea (Thunb.) DC. [Asteraceae; Atractylodis rhizoma] 10 g, Aster tataricus L.f. [Asteraceae; Asteris radix et rhizoma] 10 g, Houttuynia cordata Thunb. [Saururaceae; Houttuyniae herba] 10 g, Taraxacum sect. Taraxacum F.H.Wigg. [Asteraceae;Taraxaci herba] 10 g, Reynoutria japonica Houtt. [Polygonaceae; Polygoni cuspidati rhizoma et radix] 10 g, Astragalus mongholicus Bunge [Fabaceae; Astragali radix] 10 g.		
	Bai-Mu decoction	Xi'an International Medical Center Hospital Pharmacy	**Bai-Mu decoction:** Pueraria montana var. lobata (Willd.) Maesen & S.M.Almeida ex Sanjappa & Predeep [Fabaceae; Puerariae lobatae radix] 15 g, Angelica dahurica (Hoffm.) Benth. & Hook.f. ex Franch. & Sav. [Apiaceae;Angelicae dahuricae radix] 12 g, Magnolia sprengeri Pamp. [Magnoliaceae; Magnolia sprengeri pampan] 9 g, Wrightia laevis Hook.f. [Apocynaceae; Wrightia laevis] 30 g, Forsythia suspensa (Thunb.) Vahl [Oleaceae; Forsythiae fructus] 15 g, Fritillaria thunbergii Miq. [Liliaceae; Fritillariae thunbergii bulbus] 12 g.		
Zhao 2021b	Anti-virus No.1 formula	Jingzhou Hospital of Traditional Chinese Medicine	**Anti-virus No.1 formula:** Atractylodes lancea (Thunb.) DC. [Asteraceae; Atractylodis rhizoma] 15 g, Citrus reticulata Blanco [Rutaceae; Citri reticulatae pericarpium] 10 g, Magnolia officinalis Rehder and E.H.Wilson [Magnoliaceae; Magnoliae officinalis cortex] 10 g, Pogostemon cablin (Blanco) Benth. [Lamiaceae; Pogostemonis herba] 10 g, Amomum tsao-ko Crevost and Lemarié [Zingiberaceae; Tsaoko fructus] 15 g, Ephedra sinica Stapf [Ephedraceae; Ephedrae herba] 5 g, Hansenia weberbaueriana (Fedde ex H.Wolff) Pimenov & Kljuykov [Apiaceae; Notopterygii rhizoma et radix] 10 g, Zingiber officinale Roscoe [Zingiberaceae; Zingiberis rhizoma recens] 10 g, Prunus armeniaca L. [Rosaceae; Armeniacae semen amarum] 10g, Amomurn kravanh Pierre ex Gagnep. [Zingiberaceae; Amomi fructus rotundus] 10 g, Coix lacryma- jobi var. ma-yuen (Rom.Caill.) Stapf [Poaceae; Coicis semen] 15 g, Astragalus mongholicus Bunge [Fabaceae; Astragali radix] 20 g, Panax ginseng C.A.Mey. [Araliaceae; Ginseng Radix et Rhizoma] 20 g, Pinellia ternata (Thunb.) Makino [Araceae; Pinelliae rhizoma praeparatum] 15 g, Panax notoginseng (Burkill) F.H.Chen [Araliaceae; Notoginseng radix et rhizoma] 15 g.	N	N
Zhang 2022d	Ganjiang Xiaochaihu Decoction	Shanghai Wanshi Cheng Pharmaceutical	**Ganjiang Xiaochaihu Decoction:** Bupleurum chinense DC. [Apiaceae; Bupleuri radix] 9 g, Scutellaria baicalensis Georgi [Lamiaceae; Scutellariae radix] 12 g, Pinellia ternata (Thunb.) Makino [Araceae;	N	N

			Pinelliae rhizoma] 9 g, Panax ginseng C.A.Mey. [Araliaceae; Ginseng Radix et Rhizoma] 9 g, Zingiber officinale Roscoe [Zingiberaceae; Zingiberis rhizoma] 3 g, Glycyrrhiza uralensis Fisch. ex DC. [Fabaceae; Glycyrrhiza uralensis Fisch. ex DC. [Fabaceae; Glycyrrhizae radix et rhizoma] praeparata cum melle] 9 g, Ziziphus jujuba Mill. [Rhamnaceae; Jujubae fructus] 6 g		
Xu 2023	Reyanning mixture	Tsinghua Deren Xi'an Happiness Pharmaceutical	**Reyanning mixture:** Taraxacum sect. Taraxacum F.H.Wigg. [Asteraceae;Taraxaci herba] 372 g, Reynoutria japonica Houtt. [Polygonaceae; Polygoni cuspidati rhizoma et radix] 372 g, Thlaspi arvense L. [Brassicaceae Burnett; Patrinia villosa] 372 g, Scutellaria barbata D. Don [Labiatae; Scutellaria barbata] 186 g	Y	Y
Wang 2022	CHM decoction	Hubei Provincial Hospital of Integrated Chinese and Western Medicine	NA	N	N
Hu 2022	CHM decoction	NA	**CHM decoction 1:** Lonicera japonica Thunb. [Caprifoliaceae; Lonicerae japonicae flos] 15 g, Forsythia suspensa (Thunb.) Vahl [Oleaceae; Forsythiae fructus] 15 g, Morus alba L. [Moraceae; Mori folium] 12 g, Chrysanthemum × morifolium (Ramat.) Hemsl. [Asteraceae; Chrysanthemi flos] 12 g, Rosa damascena Mill. [Rosaceae, Rosa × damascena Herrm] 12 g, Bupleurum chinense DC. [Apiaceae; Bupleuri radix] 12 g, Platycodon grandiflorus (Jacq.) A.DC. [Campanulaceae; Platycodonis radix] 9g, Mentha canadensis L. [Lamiaceae; Menthae haplocalycis herba] 9g, Schizonepetae spica 9g, Sojae semen praeparatum 9g, Arctium lappa L. [Asteraceae; Arctii fructus] 9g, Lophatherum gracile Brongn. [Poaceae; Lophatheri herba] 9g, Scutellaria baicalensis Georgi [Lamiaceae; Scutellariae radix] 9g, Panax ginseng C.A.Mey. [Araliaceae; Ginseng Radix et Rhizoma] 9g, Pinellia ternata (Thunb.) Makino [Araceae; Pinelliae rhizoma] 9g, Zingiber officinale Roscoe [Zingiberaceae; Zingiberis rhizoma] recens 9 g, Prunus armeniaca L. [Rosaceae; Armeniacae semen amarum] 6g, Glycyrrhiza uralensis Fisch. ex DC. [Fabaceae; Glycyrrhizae radix et rhizoma] 6 g.<br>**CHM decoction 2:** Trichosanthes kirilowii Maxim. [Cucurbitaceae; Trichosanthis fructus] 20 g, Pinellia ternata (Thunb.) Makino [Araceae; Pinelliae rhizoma] 12g, Nepeta tenuifolia Benth. [Lamiaceae; Schizonepetae herba] 9g, Aster tataricus L.f. [Asteraceae; Asteris radix et rhizoma] 9g, Stemonae radix 9g, Cynanchi stauntonii rhizoma et radix 9g, Morus alba L. [Moraceae; Mori folium] 9g, Fritillaria cirrhosa D.Don [Liliaceae; Fritillariae cirrhosae bulbus] 9 g, Coptis chinensis Franch. [Ranunculaceae; Coptidis rhizoma] 6g, Platycodon grandiflorus (Jacq.) A.DC. [Campanulaceae; Platycodonis radix] 6g, Citrus reticulata Blanco [Rutaceae; Citri reticulatae pericarpium] 6g, Glycyrrhiza uralensis Fisch. ex DC. [Fabaceae; Glycyrrhizae radix et rhizoma praeparata cum melle] 6 g.<br>**CHM decoction 3:** Phragmites australis (Cav.) Trin. ex Steud. [Poaceae; Phragmitis rhizoma]20 g, Coix lacryma- jobi var. ma-yuen (Rom.Caill.) Stapf [Poaceae; Coicis semen] 12 g,Pogostemon cablin (Blanco) Benth. [Lamiaceae; Pogostemonis herba] 9g, Magnolia officinalis Rehder and E.H.Wilson [Magnoliaceae; Magnoliae officinalis cortex] 9g, Pinellia ternata (Thunb.) Makino [Araceae; Pinelliae rhizoma] 9g, Makino [Araceae; Pinelliae rhizoma praeparatum cum zingibere et alumine] 9g, Poria cocos (Schw.) Wolf [Polyporaceae; Poria] 9g, Prunus armeniaca L. [Rosaceae; Armeniacae semen amarum] 9g, Polyporus umbellatus (Pers.) Fries [Polyporaceae; Polyporus] 9g, Sojae semen praeparatum 9g, Alisma plantago-aquatica L. [Alismataceae; Alismatis rhizoma] 9g, Gardeniae fructus praeparatus 9 g,Amomurn kravanh Pierre ex Gagnep. [Zingiberaceae; Amomi fructus rotundus] 6g, Coptis chinensis Franch. [Ranunculaceae; Coptidis rhizoma] 6g, acori tatarinowii rhizoma 6 g.	N	N

Chen 2022b	CHM decoction	NA	**CHM decoction 1:** Ephedra sinica Stapf [Ephedraceae; Ephedrae herba praeparata cum melle] 6g, Prunus armeniaca L. [Rosaceae; Armeniacae semen amarum] 15g, Gypsum Fibrosum, Isatis tinctoria L. [Brassicaceae; Isatidis radix] 30g, Coix lacryma- jobi var. ma-yuen (Rom.Caill.) Stapf [Poaceae; Coicis semen] 30g, Atractylodes lancea (Thunb.) DC. [Asteraceae;Atractylodes lancea (Thunb.) DC. [Asteraceae; Atractylodis rhizoma]]10g, Pogostemon cablin (Blanco) Benth. [Lamiaceae; Pogostemonis herba] 15g, Artemisia annua L. [Asteraceae; Artemisiae annuae herba] 12g, Reynoutria japonica Houtt. [Polygonaceae; Polygoni cuspidati rhizoma et radix] 20g, Verbena officinalis L. [Verbenaceae; Verbenae herba] 30g, Phragmites australis (Cav.) Trin. ex Steud. [Poaceae; Phragmitis rhizoma] 30g, Descurainiae semen lepidii semen 15g, Citri grandis exocarpium 15g, Glycyrrhiza uralensis Fisch. ex DC. [Fabaceae; Glycyrrhizae radix et rhizoma] 10g.<br>**CHM decoction 2:** Atractylodes lancea (Thunb.) DC. [Asteraceae; Atractylodis rhizoma] 15g, Citrus reticulata Blanco [Rutaceae; Citri reticulatae pericarpium] 10g, Magnolia officinalis Rehder and E.H.Wilson [Magnoliaceae; Magnoliae officinalis cortex] 10g, Pogostemon cablin (Blanco) Benth. [Lamiaceae; Pogostemonis herba] 10g, Amomum tsao-ko Crevost and Lemarié [Zingiberaceae; Tsaoko fructus] 6g, Ephedra sinica Stapf [Ephedraceae; Ephedrae herba praeparata cum melle] 6g, Hansenia weberbaueriana (Fedde ex H.Wolff) Pimenov & Kljuykov [Apiaceae; Notopterygii rhizoma et radix] 10g,Zingiber officinale Roscoe [Zingiberaceae; Zingiberis rhizoma] recens 10g, Areca catechu L. [Arecaceae; Arecae semen] 10g.	N	N
Zhang 2022b	Shufeng Jiedu capsule	NA	**Shufeng Jiedu capsule:** Reynoutria japonica Houtt. [Polygonaceae; Polygoni cuspidati rhizoma et radix], Forsythia suspensa (Thunb.) Vahl [Oleaceae; Forsythiae fructus], Gypsum Fibrosum, Isatis tinctoria L. [Brassicaceae; Isatidis radix], Bupleurum chinense DC. [Apiaceae; Bupleuri radix], Thlaspi arvense L. [Brassicaceae Burnett; Patrinia villosa], Verbena officinalis L. [Verbenaceae; Verbenae herba], Phragmites australis (Cav.) Trin. ex Steud. [Poaceae; Phragmitis rhizoma], and Glycyrrhiza uralensis Fisch. ex DC. [Fabaceae; Glycyrrhizae radix et rhizoma].	N	N
Wang 2023	Longyizhengqi granule	Longhua Hospital	**Longyizhengqi granule:** Pogostemon cablin (Blanco) Benth. [Lamiaceae; Pogostemonis herba],Forsythia suspensa (Thunb.) Vahl [Oleaceae; Forsythiae fructus], Lonicera japonica Thunb. [Caprifoliaceae; Lonicerae japonicae flos],Atractylodes macrocephala Koidz. [Asteraceae; Atractylodis macrocephalae rhizoma],and Glycyrrhiza uralensis Fisch. ex DC. [Fabaceae; Glycyrrhizae radix et rhizoma].	N	N
Soleiman 2022	Licorice syrup	NA	**Licorice syrup:** Glycyrrhiza uralensis Fisch. ex DC. [Fabaceae; Glycyrrhizae radix et rhizoma], Ziziphus jujuba Mill. [Rhamnaceae; Jujubae fructus], Crocus sativus L. [Iridaceae; Crocus sativus L.], Rosa damascena Mill. [Rosaceae, Rosa × damascena Herrm], Rheum palmatum L. [Polygonaceae; Rhei radix et rhizoma].	N	N

NA = not applicable, Y = yes, N= no.

Table S4. Risk of bias summary in included studies.

Unique ID	D1	D2	D3	D4	D5	Overall Bias
Ai 2020-1	S	H	L	S	L	H
Ai 2020-10-LYM	S	H	L	L	L	H
Ai 2020-4	S	H	L	S	L	H
Ai 2020-9	S	H	L	S	L	H
Ai 2020-ARs	S	H	L	S	L	H
An 2021-5	L	L	H	L	S	H
Chai 2021-1	S	L	L	S	L	S
Chai 2021-2	S	L	L	L	L	S
Chai 2021-8-fever	S	L	L	L	L	S
Chai 2021-9	S	L	L	S	L	S
Chai 2021-ARs	S	L	L	S	L	S
Chen 2021-10-hsCRP	S	S	S	L	L	S
Chen 2021-10-PCT	S	S	S	L	L	S
Chen 2021-2	S	S	S	L	L	S
Chen 2021-5	S	H	L	S	L	H
Chen 2021-8-cough	S	S	S	S	S	S
Chen 2021-8-fatigue	S	S	S	S	S	S
Chen 2021-8-fever	S	S	S	L	L	S
Chen 2021-8-shortness of breath	S	S	S	S	S	S
Chen 2021-ARs	S	S	L	S	L	S
Duan 2020-5	S	S	S	S	S	S
Duan 2020-9	S	S	S	S	S	S
Duan 2020-ARs	S	S	S	S	S	S
Fu 2020-1	S	L	L	S	L	S
Fu 2020-10-CRP	S	L	L	L	L	S
Fu 2020-10-LYM	S	L	L	L	L	S
Fu 2020-10-WBC	S	L	L	L	L	S
Fu 2020-5	S	L	L	S	L	S
Fu 2020-ARs	S	L	L	S	L	S
He 2021-1	S	L	L	S	L	S
He 2021-10-ESR	S	L	L	L	L	S
He 2021-10-hsCRP	S	L	L	L	L	S
He 2021-9	S	L	L	S	L	S
Hu 2021a-2	S	H	H	L	S	H
Hu 2021a-4	S	H	H	S	S	H
Hu 2021a-5	S	H	H	S	S	H
Hu 2021a-ARs	S	H	H	S	S	H
Hu 2021b-1	L	L	L	L	L	L
Hu 2021b-2	L	L	L	L	L	L
Hu 2021b-3	L	L	L	L	L	L
Hu 2021b-5	L	L	L	L	L	L

Hu 2021b-8-cough	L	L	L	L	L	L
Hu 2021b-8-fatigue	L	L	L	L	L	L
Hu 2021b-8-fever	L	L	L	L	L	L
Hu 2021b-ARs	L	L	L	L	L	L
Li 2020-1	S	L	L	S	L	S
Li 2020-10-WBC	S	L	L	L	L	S
Li 2020-4	S	L	L	S	L	S
Li 2020-ARs	S	L	L	S	L	S
Liao 2020-ARs	S	L	L	S	L	S
Liu 2021a-2	L	L	L	L	L	L
Liu 2021a-3	L	L	L	L	L	L
Liu 2021a-5	L	L	L	S	L	S
Liu 2021a-8-fever	L	L	L	L	L	L
Liu 2021a-ARs	L	L	L	S	L	S
Liu 2021b-1	S	L	L	S	L	S
Liu 2021b-ARs	S	L	L	S	L	S
Luo 2021-10-CRP	L	L	S	L	L	S
Luo 2021-10-LYM	L	L	S	L	L	S
Luo 2021-10-TNF	L	L	S	L	L	S
Luo 2021-10-WBC	L	L	S	L	L	S
Luo 2021-5	L	L	H	L	L	H
Luo 2021-6	L	L	L	L	L	L
Luo 2021-7	L	L	H	L	L	H
Luo 2021-8-cough	L	L	S	L	L	S
Luo 2021-8-fatigue	L	L	S	L	L	S
Luo 2021-8-fever	L	L	S	L	L	S
Luo 2021-8-shortness of breath	L	L	S	L	L	S
Luo 2021-ARs	L	L	L	L	L	L
Ni 2021-10-TNF	L	H	L	L	L	H
Ni 2021-4	L	H	L	S	L	H
Ni 2021-ARs	L	H	L	S	L	H
Ping 2021-1	S	L	L	S	L	S
Ping 2021-10-ESR	S	L	H	L	S	H
Ping 2021-10-hsCRP	S	L	H	L	S	H
Ping 2021-10-LYM	S	L	H	L	S	H
Ping 2021-2	S	L	H	L	S	H
Ping 2021-4	S	L	H	S	S	H
Ping 2021-ARs	S	L	H	S	S	H
Qiu 2020-3	S	L	L	S	L	S
Qiu 2020-5	S	L	L	L	L	S
Qiu 2020-8-cough	S	L	L	S	L	S
Qiu 2020-8-fever	S	L	L	L	L	S

Qiu 2020-9	S	L	L	S	L	S
Sun 2020-3	S	H	L	S	L	H
Sun 2020-5	S	H	L	S	L	H
Sun 2020-8-cough	S	H	L	S	L	H
Sun 2020-ARs	S	H	L	S	L	H
Sun 2021-1	L	L	L	L	L	L
Sun 2021-8-fever	L	L	L	L	L	L
Sun 2021-9	L	L	L	S	L	S
Sun 2021-ARs	L	L	L	S	L	S
Tan 2021-1	H	L	L	S	L	H
Wang 2020a-3	S	L	L	S	L	S
Wang 2020b-2	S	L	L	L	L	S
Wang 2020b-3	S	L	L	S	L	S
Wang 2020b-4	S	L	L	S	L	S
Wang 2020b-8-fever	S	L	L	L	L	S
Wang 2020c-10-NEU	L	S	L	L	L	S
Wang 2020c-10-WBC	L	S	L	L	L	S
Wang 2020c-2	L	S	L	L	L	S
Wang 2020c-3	L	S	L	L	L	S
Wang 2020c-7	L	H	L	L	L	H
Wang 2020c-8-fever	L	S	L	L	L	S
Wang 2020c-ARs	L	S	L	L	L	S
Wang 2021-1	S	L	L	S	L	S
Wang 2021-10-CRP	S	L	L	L	L	S
Wang 2021-10-LYM	S	L	L	L	L	S
Wang 2021-10-WBC	S	L	L	L	L	S
Wang 2021-4	S	L	L	S	L	S
Wang 2021-9	S	L	L	S	L	S
Wang 2021-ARs	S	L	L	S	L	S
Wen 2020-10-CRP	S	L	L	L	L	S
Wen 2020-10-ESR	S	L	L	L	L	S
Wen 2020-10-LYM	S	L	L	L	L	S
Wen 2020-10-WBC	S	L	L	L	L	S
Wen 2020-5	S	L	L	S	L	S
Wen 2020-6	S	L	L	S	L	S
Wen 2020-ARs	S	L	L	S	L	S
Xiao 2020-5	L	L	H	L	L	H
Xiong 2020-ARs	S	L	L	S	L	S
Xu 2021-2	H	L	L	L	L	H
Xu 2021-4	H	L	L	S	L	H
Xu 2021-7	H	L	L	L	L	H
Xu 2021-8-fever	H	L	L	L	L	H

Xu 2021-ARs	H	L	L	S	L	H
Yang 2021-1	S	L	L	S	L	S
Yang 2022-10-CRP	S	L	L	L	L	S
Yang 2022-10-ESR	S	L	L	L	L	S
Yang 2022-10-LYM	S	L	L	L	L	S
Yang 2022-10-NEU	S	L	L	L	L	S
Yang 2022-10-PCT	S	L	L	L	L	S
Yang 2022-10-WBC	S	L	L	L	L	S
Yang 2022-2	S	L	L	L	L	S
Ye 2020-1	L	L	L	L	L	L
Ye 2020-10-CRP	L	L	L	L	L	L
Ye 2020-10-ESR	L	L	L	L	L	L
Ye 2020-10-hsCRP	L	L	L	L	L	L
Ye 2020-10-LYM	L	L	L	L	L	L
Ye 2020-10-NEU	L	L	L	L	L	L
Ye 2020-10-PCT	L	L	L	L	L	L
Ye 2020-10-WBC	L	L	L	L	L	L
Ye 2020-3	L	L	H	L	L	H
Ye 2020-5	L	L	L	L	L	L
Ye 2020-6	L	L	L	L	L	L
Ye 2020-7	L	L	L	L	L	L
Ye 2021-1	S	L	L	S	L	S
Ye 2021-10-CRP	S	L	L	L	L	S
Ye 2021-10-LYM	S	L	L	L	L	S
Ye 2021-10-NEU	S	L	L	L	L	S
Ye 2021-10-WBC	S	L	L	L	L	S
Ye 2021-ARs	S	L	L	S	L	S
Yu 2020-1	S	L	L	S	L	S
Yu 2020-10-CRP	S	L	L	L	L	S
Yu 2020-10-LYM	S	L	L	L	L	S
Yu 2020-10-PCT	S	L	L	L	L	S
Yu 2020-10-WBC	S	L	L	L	L	S
Yu 2020-3	S	L	L	L	L	S
Yu 2020-5	S	L	L	S	L	S
Yu 2020-7	S	L	L	L	L	S
Yu 2020-ARs	S	L	L	S	L	S
Zeng 2021-10-LYM	L	L	L	L	L	L
Zeng 2021-10-NEU	L	L	L	L	L	L
Zeng 2021-10-WBC	L	L	L	L	L	L
Zeng 2021-2	L	L	L	L	L	L
Zeng 2021-4	L	L	L	L	L	L
Zeng 2021-5	L	L	L	L	L	L

Zeng 2021-8-cough	L	L	L	L	L	L
Zeng 2021-8-fatigue	L	L	L	L	L	L
Zeng 2021-8-fever	L	L	L	L	L	L
Zeng 2021-8-shortness of breath	L	L	L	L	L	L
Zeng 2021-9	L	L	L	L	L	L
Zeng 2021-ARs	L	L	L	L	L	L
Zhang 2020-5	S	L	L	S	L	S
Zhang 2020-ARs	S	L	L	S	L	S
Zhang 2021-2	L	L	L	L	L	L
Zhang 2021-5	L	L	L	L	L	L
Zhang 2021-8-cough	L	L	L	L	L	L
Zhang 2021-8-fever	L	L	L	L	L	L
Zhang 2021-ARs	L	L	L	L	L	L
Zhang 2022-1	L	L	L	S	L	S
Zhang 2022-3	L	L	L	S	L	S
Zhang 2022-7	L	L	L	L	L	L
Zhang 2022-8-cough	L	L	L	S	L	S
Zhang 2022-8-fever	L	L	L	L	L	L
Zhang 2022-ARs	L	L	L	S	L	S
Zhao 2020-1	S	L	L	S	L	S
Zhao 2020-10-CRP	S	L	L	L	L	S
Zhao 2020-10-LYM	S	L	L	L	L	S
Zhao 2020-10-TNF	S	L	L	L	L	S
Zhao 2020-10-WBC	S	L	L	L	L	S
Zhao 2020-4	S	L	L	S	L	S
Zhao 2020-8-fever	S	L	L	L	L	S
Zhao 2021a-1	L	L	L	S	L	S
Zhao 2021a-4	L	L	L	S	L	S
Zhao 2021a-5	L	L	L	S	L	S
Zhao 2021a-7	L	L	L	L	L	L
Zhao 2021a-ARs	L	L	L	S	L	S
Zhao 2021b-1	S	L	L	S	L	S
Zhao 2021b-10-CRP	S	L	L	L	L	S
Zhao 2021b-10-LYM	S	L	L	L	L	S
Zhao 2021b-10-PCT	S	L	L	L	L	S
Zhao 2021b-3	S	L	L	S	L	S
Zhao 2021b-9	S	L	L	S	L	S
Zheng 2020-1	S	L	L	S	L	S
Zhou 2021-1	L	L	L	L	L	L
Zhou 2021-5	L	L	L	L	L	L
Zhou 2021-6	L	L	L	L	L	L
Zhou 2021-7	L	L	L	L	L	L

Zhou 2021-ARs	L	L	L	L	L	L
Zhang 2022b-1	S	L	L	L	S	S
Zhang 2022b-ARs	S	L	L	L	S	S
Zhang 2022b-5	S	L	L	L	S	S
Zhang 2022d-1	S	L	L	L	S	S
Zhang 2022d-2	S	L	L	L	S	S
Zhang 2022d-9	S	L	L	L	S	S
Wang 2022-1	S	L	L	L	S	S
Wang 2022-2	S	L	L	L	S	S
Wang 2022-7	S	L	L	L	S	S
Wang 2022-9	S	L	L	L	S	S
Wang 2022-10-2-WBC	S	L	L	L	S	S
Wang 2022-10-2-LYM	S	L	L	L	S	S
Wang 2022-10-2-CRP	S	L	L	L	S	S
Wang 2022-10-2-ESR	S	L	L	L	S	S
Wang 2022-ARs	S	L	L	L	S	S
Hu 2022-1	S	L	L	L	S	S
Hu 2022-4	S	L	L	L	S	S
Hu 2022-5	S	L	L	L	S	S
Hu 2022-7	S	L	L	L	S	S
Hu 2022-8-3-fever	S	L	L	L	S	S
Hu 2022-ARs	S	L	L	L	S	S
Chen 2022-1	S	L	L	L	S	S
Chen 2022-2	S	L	L	L	S	S
Chen 2022-8-3-fever	S	L	L	L	S	S
Chen 2022-10-2-WBC	S	L	L	L	S	S
Chen 2022-10-2-CRP	S	L	L	L	S	S
Wang 2023-2	H	L	L	L	L	H
Wang 2023-4	H	L	L	L	L	H
Wang 2023-ARs	H	L	L	L	L	H
Soleiman 2022-4	L	L	L	L	L	L
Soleiman 2022-5	L	L	S	L	L	S
Soleiman 2022-7	L	L	L	L	L	L
Soleiman 2022-ARs	L	L	H	L	L	H
Xu 2023-2	L	L	L	L	L	L
Xu 2023-ARs	L	L	H	L	L	H
Xu 2023-5	L	L	L	L	L	L

D1 = randomization process; D2 = deviations from intended interventions; D3 = missing outcome data; D4 = measurement of the outcome; D5 = selection of the reported result; L = low risk; S = some concerns; H = high risk; 1 = clinical efficacy; 2 = SARS-CoV-2 nucleic acid conversion time; 3 = chest image improvement; 4 = duration of hospitalization; 5 = conversion to severe cases; 6 = conversion to mild cases; 7 = death; 8 = clinical symptoms recovery time; 9 = total score of TCM syndrome; 10 = laboratory indicators; CRP = C-reactive protein; LYM = lymphocyte; TNF-α = tumor necrosis factor-α; WBC = white blood cell; NEU = neutrophil; hsCRP = high sensitive C-reactive protein; ESR = erythrocyte sedimentation rate; PCT = procalcitonin; ARs = adverse reactions

Table S5. Incidence and difference of adverse reactions between two groups.

**CHM preparations**	**Study**	**Sample size (I/C)**	**Adverse reactions**	**Intervention group**		**Control group**		***P* value**
				**Event**	**%**	**Event**	**%**	
Jinhua Qinggan granules	Duan 2020	82/41	Diarrhea	27	32.9	0	0.0	**< 0.0001**
Shuanghuanglian oral liquids	Ni 2021	176 (Low dose: 56; Middle dose: 61; High dose: 59)/59	Rash	Low dose: 0	0.0	0	0.0	NA
				Middle dose: 1	1.6			1.0000
				High dose: 2	3.4			0.4957
				Total: 3	1.7			0.5745
			Skin allergies	Low dose: 1	1.8	0	0.0	0.4870
				Middle dose: 0	0.0			NA
				High dose: 0	0.0			NA
				Total: 1	0.6			1.0000
			Gastrointestinal dysfunction	Low dose: 0	0.0	0	0.0	NA
				Middle dose: 3	4.9			0.2439
				High dose: 2	3.4			0.4957
				Total: 5	2.8			0.3346
			Nausea	Low dose: 1	1.8	0	0.0	0.4914
				Middle dose: 0	0.0			NA
				High dose: 1	1.7			1.0000
				Total: 2	1.1			1.0000
			Vomiting	Low dose: 0	0.0	0	0.0	NA
				Middle dose: 0	0.0			NA
				High dose: 2	3.4			0.4957
				Total: 2	1.1			1.0000
			Diarrhea	Low dose: 1	1.8	0	0.0	0.487
				Middle dose: 0	0.0			NA
				High dose: 1	1.7			1.0000

		Total: 2	1.1			1.0000
	Abdominal discomfort	Low dose: 0	0.0	0	0.0	NA
		Middle dose: 0	0.0			NA
		High dose: 1	1.7			1.0000
		Total: 1	0.6			1.0000
	Loss of appetite	Low dose: 0	0.0	0	0.0	NA
		Middle dose: 1	1.6			1.0000
		High dose: 0	0.0			NA
		Total: 1	0.6			1.0000
	Hypokalemia	Low dose: 0	0.0	0	0.0	NA
		Middle dose: 1	1.6			1.0000
		High dose: 0	0.0			NA
		Total: 1	0.6			1.0000
	Constipation	Low dose: 0	0.0	1	1.7	1.0000
		Middle dose: 0	0.0			0.4917
		High dose: 0	0.0			1.0000
		Total: 0	0.0			0.2521
	Urinary tract infection	Low dose: 1	1.8	0	0.0	0.4914
		Middle dose: 0	0.0			NA
		High dose: 0	0.0			NA
		Total: 1	0.6			1.0000
	Increased aspartate aminotransferase	Low dose: 2	3.6	0	0.0	0.2349
		Middle dose: 0	0.0			NA
		High dose: 1	1.7			1.0000
		Total: 3	1.7			0.5745
	Increased alanine aminotransferase	Low dose: 3	5.4	1	1.7	0.3555

				Middle dose: 0	0.0			0.4917
				High dose: 1	1.7			1.0000
				Total: 4	2.3			1.0000
			Increased g-glutamyl transpeptidase	Low dose: 3	5.4	0	0.0	0.1123
				Middle dose: 0	0.0			NA
				High dose: 1	1.7			1.0000
				Total: 4	2.3			0.5745
			Hyperlipidemia	Low dose: 0	0.0	0	0.0	NA
				Middle dose: 2	3.3			0.2349
				High dose: 0	0.0			NA
				Total: 2	1.1			1.0000
Qingfei Paidu decoction	Li 2020	6/6	Itchy skin	1	1.7	0	0.0	1.0000
	Wang 2021	70/70	Flustered	0	0.0	2	2.9	0.4964
			Nausea	1	1.4	4	5.7	0.3659
			Fatigue	1	1.4	3	4.3	0.6195
Huashibaidu granule	Zhao 2021a	168/195	Diarrhea	6	3.6	2	1.0	0.1513
	Liu 2021a	100/102	Diarrhea	8	8.0	7	6.9	0.7944
			Abdominal discomfort	2	2.0	3	2.9	1.0000
			Loss of appetite	3	3.0	0	0.0	0.1195
			Anxiety	0	0.0	2	2.0	0.4976
			Oral ulcer	1	1.0	1	1.0	1.0000
			Shortness of breath	0	0.0	2	2.0	0.4976
			Constipation	0	0.0	1	1.0	1.0000
			Vomiting	1	1.0	0	0.0	0.495
			Itchy skin	0	0.0	1	1.0	1.0000
			Lower extremity edema	0	0.0	1	1.0	1.0000

			Dry eye	1	1.0	0	0.0	0.495
			Limb pain	0	0.0	1	1.0	1.0000
Jinyinhua oral liquid	Hu 2021a	116 (60 mL: 59; 120 mL: 57)/71	Diarrhea	60 mL: 0	0.0	0	0.0	NA
				120 mL: 1	1.8			0.4453
				Total: 1	0.9			1.0000
	Zhang 2020	80/40	Diarrhea	1	1.25	0	0.0	1.0000
Xuebijing injection	Luo 2021	29/28	Abnormal liver function	5	17.2	3	10.7	0.7057
			Renal dysfunction	3	10.3	4	14.3	0.7057
			Rash	2	6.9	1	3.6	1.0000
	Wen 2020	60/60	Total adverse reactions	0	0.0	NA	NA	NA
Keguan−1	Wang 2020c	24/24	Diarrhea	9	37.5	8	33.3	1.0000
			Loss of appetite	4	16.7	5	20.8	1.0000
			Nausea	2	8.3	3	12.5	1.0000
			Stomach pain	2	8.3	3	12.5	1.0000
			Allergic reaction	0	0.0	1	4.2	1.0000
			Sepsis	0	0.0	1	4.2	1.0000
Xiyanping injection	Zhang 2021	65/65	Total laboratory adverse reactions	45	69.2	38	58.8	0.2734
			Lymphocytopenia	13	20	11	16.9	0.8212
			Neutrophilia	14	21.5	8	12.3	0.2422
			Increased C-reactive protein	5	7.7	9	13.8	0.3969
			Increased alanine aminotransferase	7	10.8	7	10.8	1.0000
			Hyperbilirubinaemia	9	13.8	5	7.7	0.3969
			Decreased blood creatine kinase	8	12.3	3	4.6	0.2061
			Increased lactate dehydrogenase	5	7.7	6	9.2	1.0000
			Decreased white blood cell count	5	7.7	5	7.7	1.0000
			Chest pain	15	23.1	11	16.9	0.5107

			Diarrhea	10	15.4	15	23.1	0.3734
			Nausea	11	16.9	8	12.3	0.6195
			Fatigue	6	9.2	4	6.2	0.7439
			Abdominal discomfort	3	4.6	5	7.7	0.7178
			Shortness of breath	4	6.2	4	6.2	1.0000
			Dizziness	4	6.2	3	4.6	1.0000
Shenhuang granule	Zhou 2021		Total adverse reactions	56	98.2	54	100	1.0000
			Hypoalbuminemia	21	36.8	38	70.4	**0.0008**
			Hypokalemia	4	7.0	9	16.7	0.1448
			Increased blood glucose	30	52.6	43	79.6	**0.0002**
			Anemia	29	50.9	29	53.7	0.9141
			Rash	1	1.8	0	0.0	1.0000
			Thrombocytopenia	21	36.8	36	66.7	**0.0032**
			Increased total bilirubin	7	12.3	19	35.2	**0.0087**
			Increased blood lipids	19	33.3	24	44.4	0.3143
			Increased white blood cell count	23	40.4	43	79.6	**< 0.0001**
		57/54	Increased blood urea nitrogen	18	31.6	31	57.4	**0.0108**
			Increased neutrophil	30	52.6	49	90.7	**< 0.0001**
			Aspartate aminotransferase increased	23	40.4	35	64.8	**0.0169**
			Constipation	2	3.5	0	0.0	0.4958
			Nausea	1	1.8	4	7.4	0.3283
			Diarrhea	2	3.5	9	16.7	**0.0454**
			Vomiting	1	1.8	4	7.4	0.1980
			Abnormal serum sodium	7	12.3	29	53.7	**< 0.0001**
			Increased serum potassium	10	17.5	27	50	**0.0006**
			Total serious adverse reactions	45	78.9	53	98.1	**0.0044**

			Respiratory failure or acute respiratory distress syndrome	23	40.4	43	79.6	**< 0.0001**
			Cardiopulmonary failure	8	14.0	31	57.4	**< 0.0001**
			Pulmonary embolism	0	0.0	2	3.7	0.2344
			Cardiac arrest	16	28.1	30	55.6	**0.0060**
			Acute coronary syndrome	1	1.8	1	1.9	1.0000
			Tachycardia	6	10.5	6	11.1	1.0000
			Septic shock	6	10.5	5	9.3	1.0000
			Sepsis	7	12.3	4	7.4	0.5290
			Bronchitis	4	7.0	1	1.9	0.3644
			Thrombocytopenia	15	26.3	34	63	**0.0002**
			Increased D-dimer	43	75.4	51	94.4	**0.0119**
			Hemorrhage of lower digestive tract	4	7.0	0	0.0	0.1188
			Acute kidney injury	7	12.3	12	22.2	0.2552
			Multiple organ dysfunction syndrome	9	15.8	28	51.9	**0.0001**
Lianhua Qingwen capsule/granule	Zhang 2022	72/72	Heart dysfunction	0	0.0	1	1.39	1.0000
			Gastrointestinal dysfunction	1	1.39	6	8.33	0.1158
			Hepatobiliary dysfunction	1	1.39	1	1.39	1.0000
			Infections	1	1.39	1	1.39	1.0000
			Metabolic disorders	3	4.17	2	2.78	1.0000
			Neurological disorders	1	1.39	0	0.0	1.0000
	Sun 2020	57/57	Total adverse reactions	0	0.0	NA	NA	NA
	Chen 2021	28/29	Nausea and vomiting	2	7.1	2	6.9	1.0000
			Diarrhea	2	7.1	1	3.5	0.6115
			Abnormal liver function	4	14.3	3	10.34	0.7057
			Dizziness	1	3.6	2	6.9	1.0000

	Hu 2021b	142/142	Abnormal liver function	32	22.5	32	22.5	1.0000
			Renal dysfunction	8	5.6	11	7.7	0.6348
			Headache	1	0.7	1	0.7	1.0000
			Nausea	6	4.2	5	3.5	1.0000
			Vomiting	2	1.4	3	2.1	1.0000
			Diarrhea	8	5.6	19	13.4	**0.0431**
			Loss of appetite	8	5.6	6	4.2	0.7840
	Yu 2020	147/148	Total adverse reactions	0	0.0	0	0.0	NA
Reduning injection	Xu 2021	77/80	Death	0	0.0	3	3.8	0.2455
			Abnormal liver function	0	0.0	2	2.5	0.497
			Nausea	2	2.6	1	1.3	0.6154
			Diarrhea	1	1.3	0	0.0	0.4904
			Bitter taste	0	0.0	1	1.3	1.0000
			Stomach discomfort	1	1.3	0	0.0	0.4904
			Loss of appetite	0	0.0	1	1.3	1.0000
Jiawei Yupingfeng powder	Ping 2021	30/24	Nausea, vomiting, loss of appetite	3	10.00	8	33.33	0.0459
			Diarrhea	1	3.33	1	4.2	1.0000
			Chest tightness	2	6.67	2	8.3	1.0000
			Itchy skin	0	0.00	1	4.2	0.4444
CHM decoction	Liao 2020	35/35	Rash	0	0.0	1	2.9	1.0000
			Insomnia	1	2.86	4	11.4	0.3565
			Tremor	1	2.86	2	5.7	1.0000
			Itchy skin	1	2.86	2	5.7	1.0000
	Hu 2022	43/43	Diarrhea	1	2.33	0	0.0	1.0000
			Abnormal liver function	0	0.0	2	4.65	0.4941
	Wang 2022	80/40	Total adverse reactions	0	0.0	0	0.0	NA

Lianhua Qingwen capsule + “Pneumonia No.2” formula	Liu 2021b	44/44	Diarrhea	4	9.09	1	2.3	0.3604
			Headache	1	2.27	0	0.0	1.0000
			Dizziness	2	4.55	0	0.0	0.4943
			Nausea and vomiting	5	11.36	2	4.6	0.4336
“Pneumonia No.1” formula, “Pneumonia No.2” formula, “Pneumonia No.3” formula	Chai 2021	96/41	Total adverse reactions	0	0.0	0	0.0	NA
Toujie Quwen granule	Fu 2020	37/36	Total adverse reactions	0	0.0	0	0.0	NA
Liu Shen capsule	Sun 2021	40/40	Total adverse reactions	0	0.0	0	0.0	NA
Modified Shengjiang Powder	Ye 2021	50/50	Total adverse reactions	0	0.0	0	0.0	NA
Qi-nourishing essence-replenishing decoction + Hu-Huang decoction + Bai-Mu decoction	Yang 2022	20/20	Total adverse reactions	0	0.0	0	0.0	NA
Maxingshigan-Weijing decoction	Zeng 2021	30/29	Total adverse reactions	0	0.0	0	0.0	NA
“Pneumonia No.1” formula	Ai 2020	98/98	Total adverse reactions	0	0.0	NA	NA	NA
Xuanfei Baidu decoction	Xiong 2020	42/42	Total adverse reactions	0	0.0	NA	NA	NA
Licorice syrup	Soleiman 2022	91/104	Myocardial Infarction or Unstable angina	5	5.49	7	6.73	0.7736
			Creatinine rising more than 25%	3	3.30	2	1.92	0.6658
Longyizhengqi granule	Wang 2023	667/2576	Diarrhea	17	2.55	0	0.0	<0.0001
Shufeng Jiedu capsule	Zhang 2022b	117/117	Nausea	7	5.9	5	4.3	1.0000
			Diarrhea	4	3.4	6	5.1	1.0000
			Vomiting	3	2.6	4	3.4	1.0000
			Abdominal pain	6	5.1	5	4.3	1.0000
			Loss of appetite	5	4.3	4	3.4	1.0000
			Headache	3	2.6	6	5.1	0.4992
Reyanning mixture	Xu 2023	1411/1407	Diarrhea	30	2.1	33	2.3	0.8120

	Gastralgia	37	2.6	35	2.5	0.8905
	Insomnia	20	1.4	30	2.1	0.2044
	Constipation	15	1.1	17	1.2	0.8526
	Chest tightness	11	0.8	11	0.8	1.0000
	Dyspepsia	5	0.4	3	0.2	0.7262
	Flatulence	7	0.5	6	0.4	1.0000
	Dizziness	7	0.5	1	0.1	0.0699
	Headache	12	0.9	7	0.5	0.9959
	Limb pain	7	0.5	7	0.5	1.0000
	Conjunctivitis	5	0.4	4	0.3	1.0000
	Rash	6	0.4	7	0.5	0.9959

I = Chinese herbal medicine plus conventional western medicine group; C = conventional western medicine group. Bold prints indicate statistically significant effects.

Figure S1. Forest plot of the results of subgroup analysis by study design for clinical effective rate.

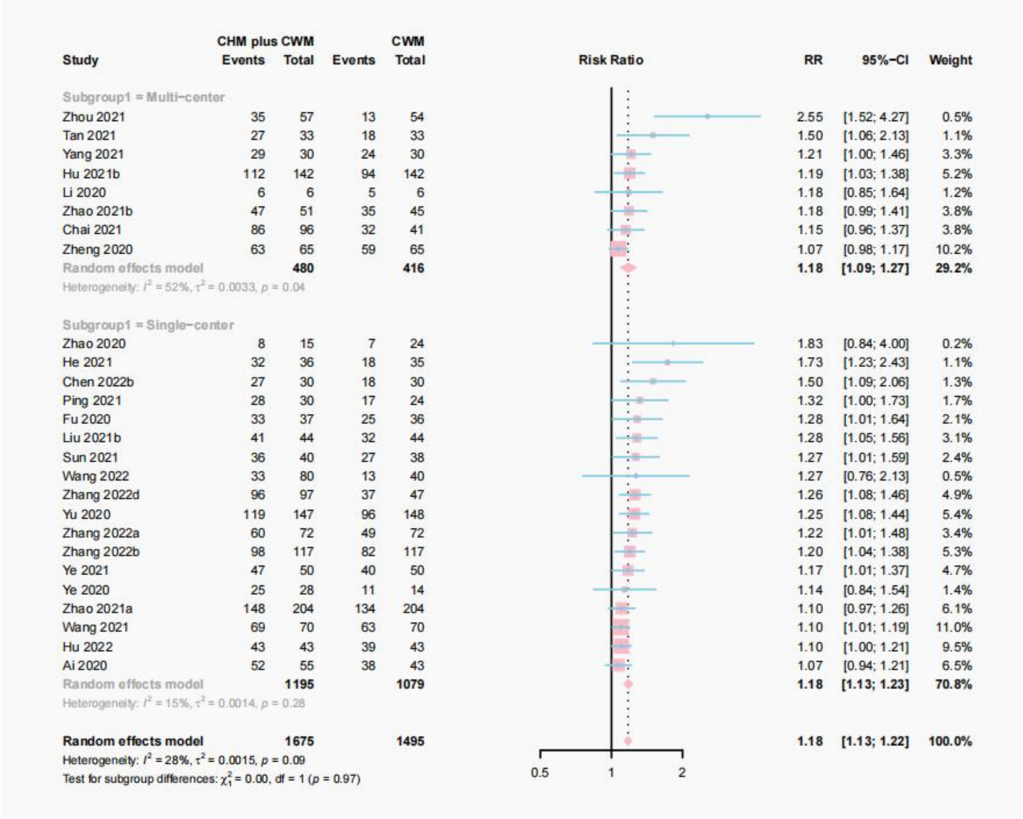

Figure S2. Forest plot of the results of subgroup analysis by disease severity for clinical effective rate.

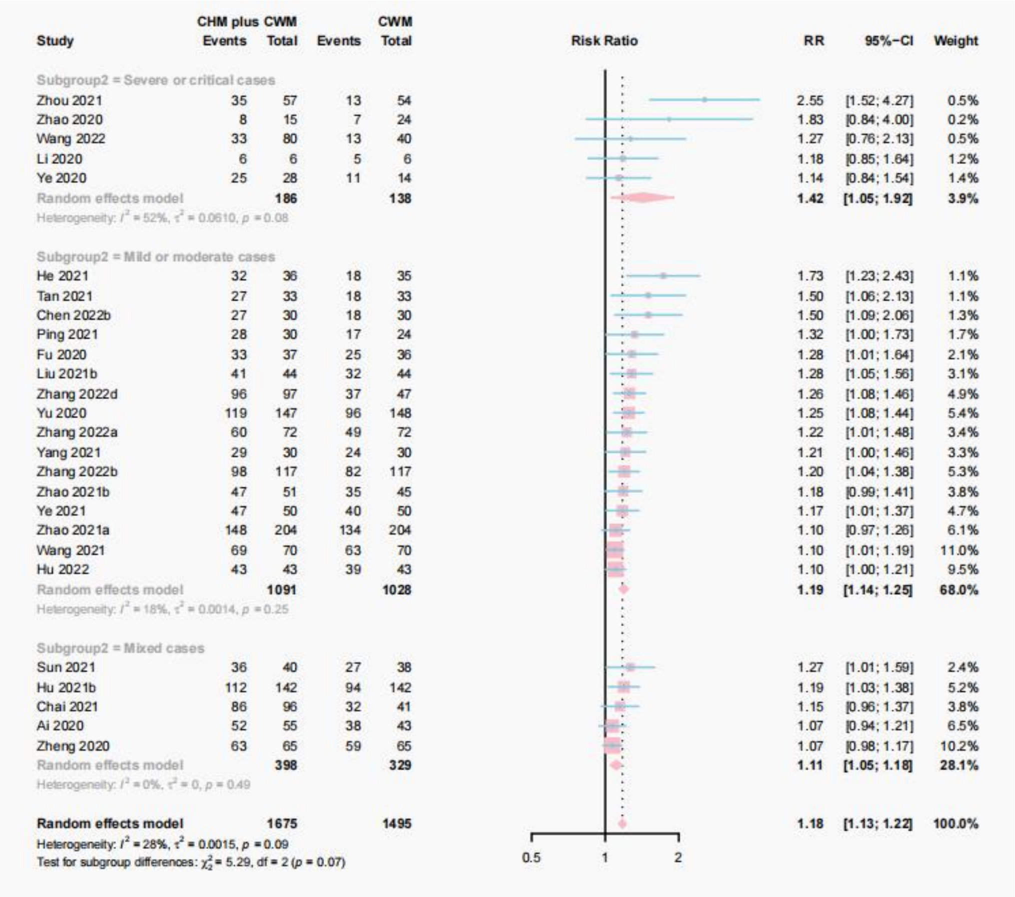

Figure S3. Forest plot of the results of subgroup analysis by treatment days for clinical effective rate.

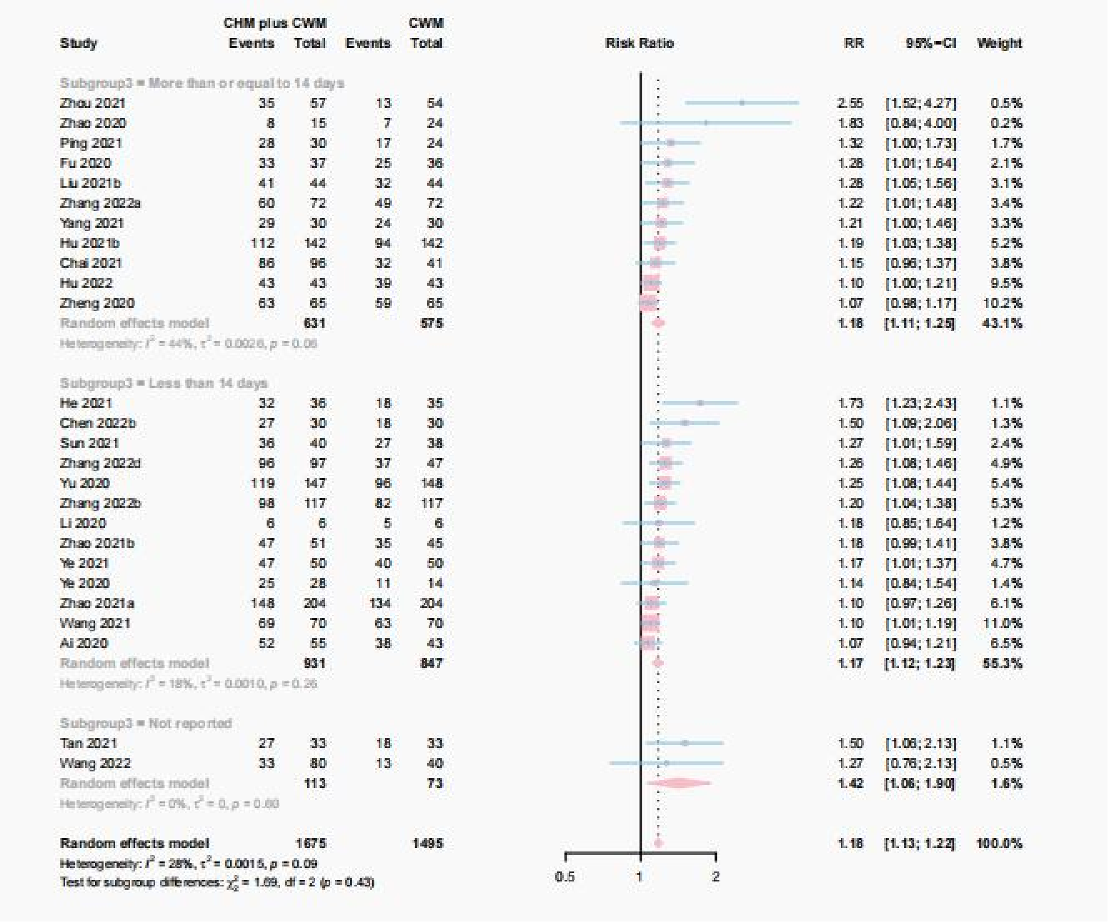

Figure S4. Forest plot of the results of subgroup analysis by CHM type for clinical effective rate.

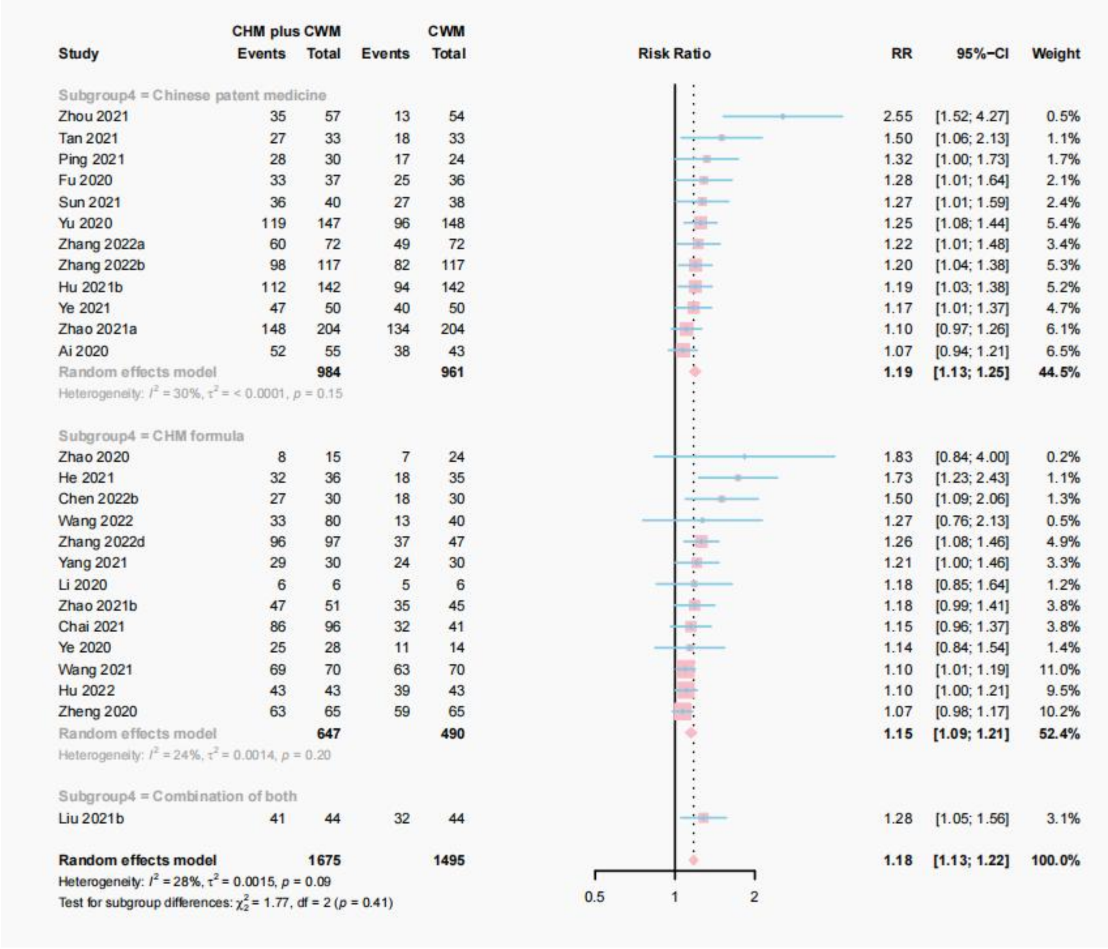

Figure S5. Forest plot of the results of subgroup analysis by study design for SARS-CoV-2 nucleic acid conversion time.

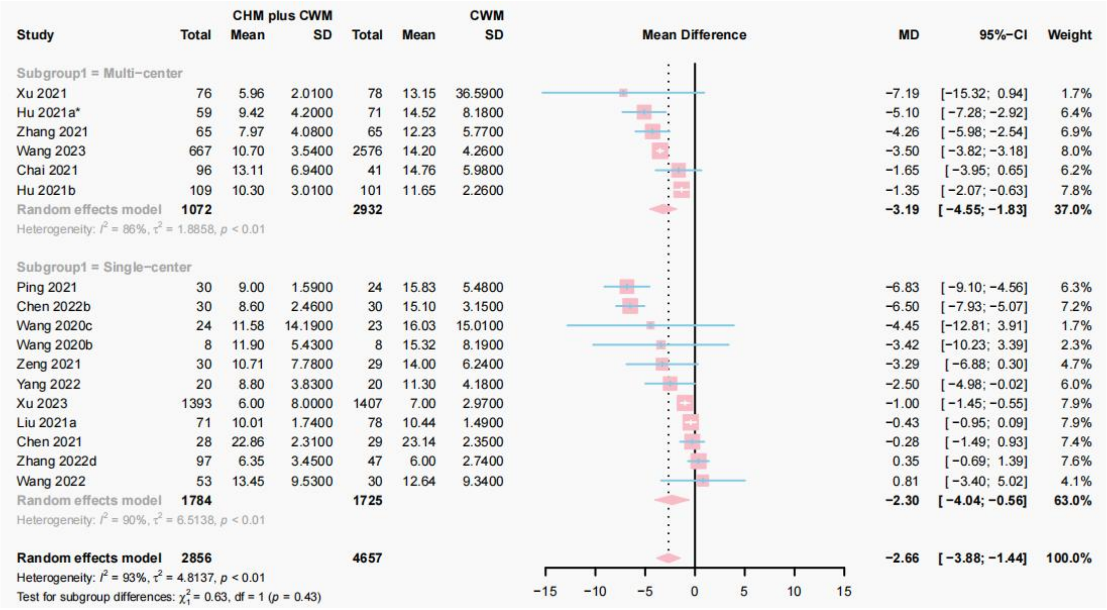

Figure S6. Forest plot of the results of subgroup analysis by with or without symptoms for SARS-CoV-2 nucleic acid conversion time.

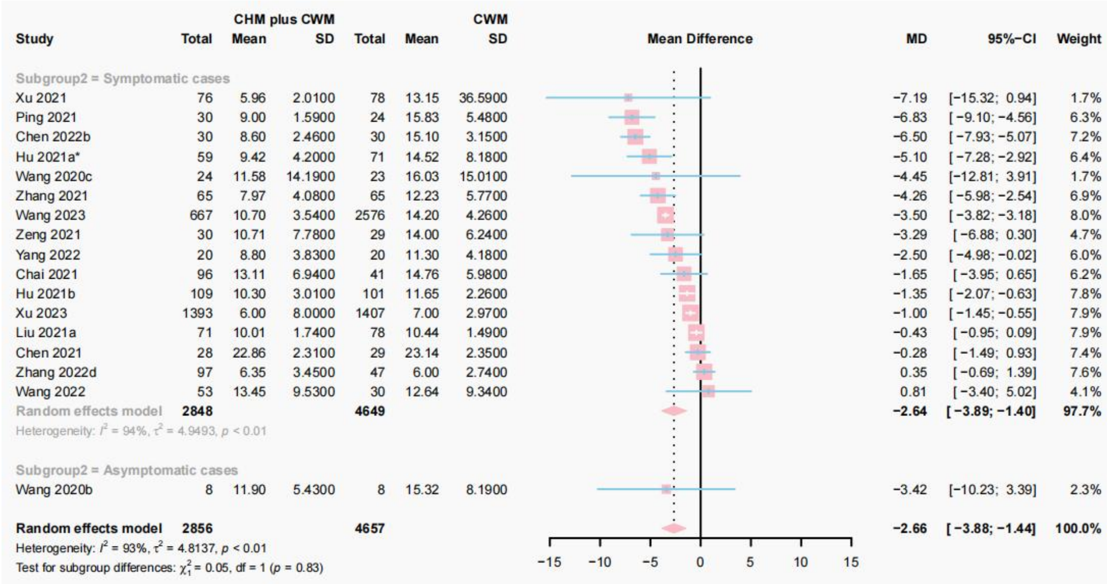

Figure S7. Forest plot of the results of subgroup analysis by treatment days for SARS-CoV-2 nucleic acid conversion time.

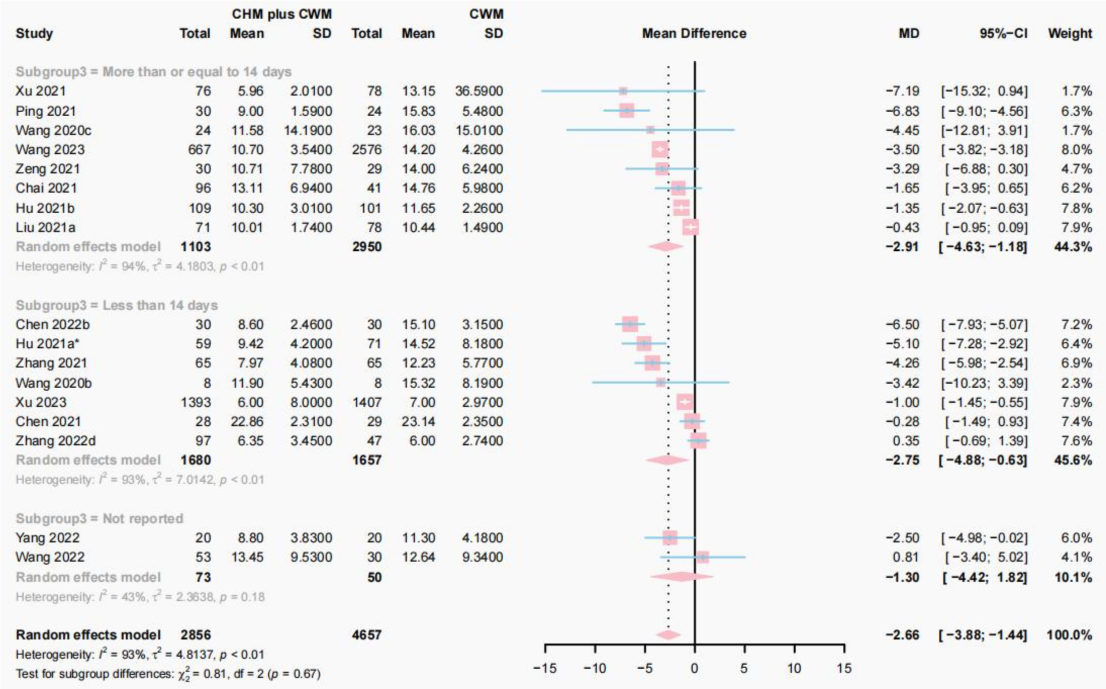

Figure S8. Forest plot of the results of subgroup analysis by administration method for SARS-CoV-2 nucleic acid conversion time.

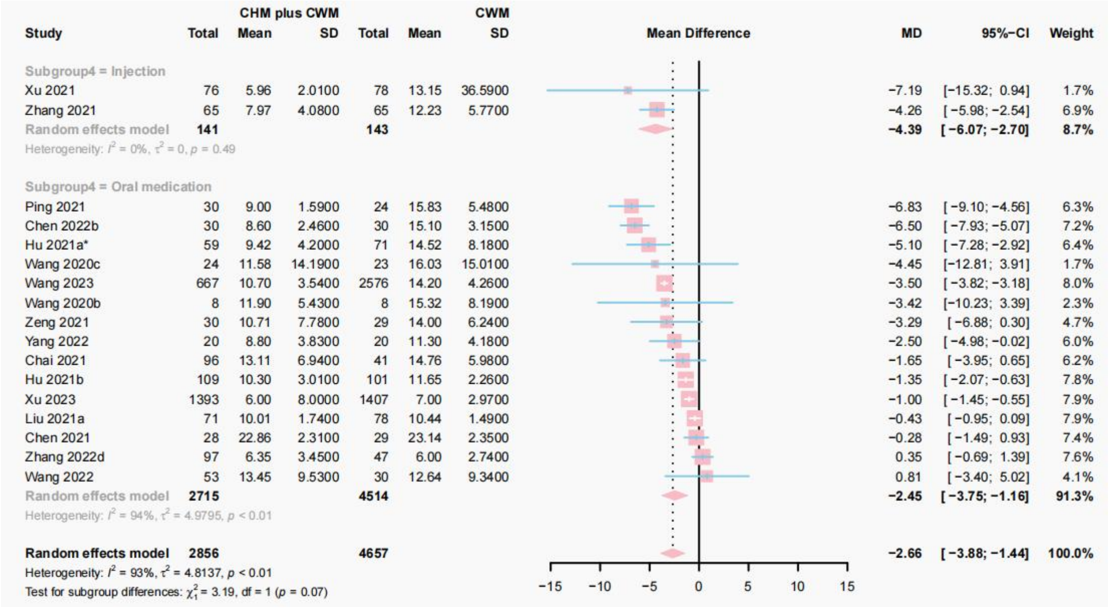

Figure S9. Forest plot of the results of leave-one-out analysis for clinical effective rate.

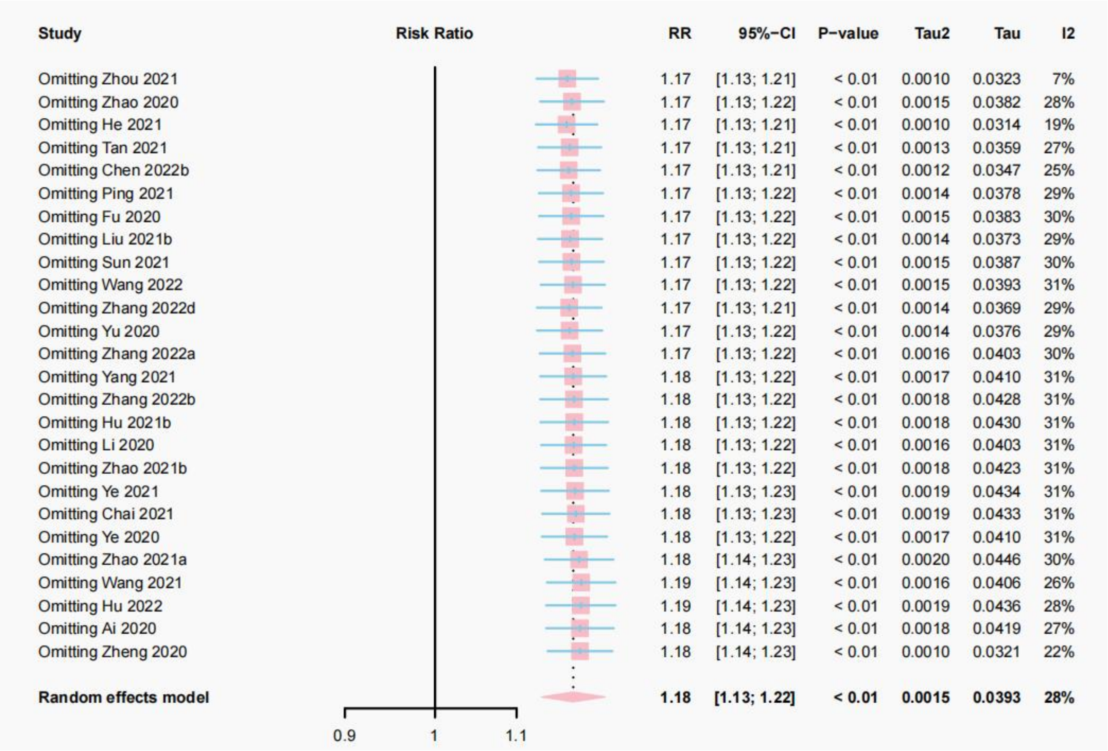

[*] Data from the 60 mL CHM group were included in meta-analysis.

Figure S10. Forest plot of the results of leave-one-out analysis for SARS-CoV-2 nucleic acid conversion time.

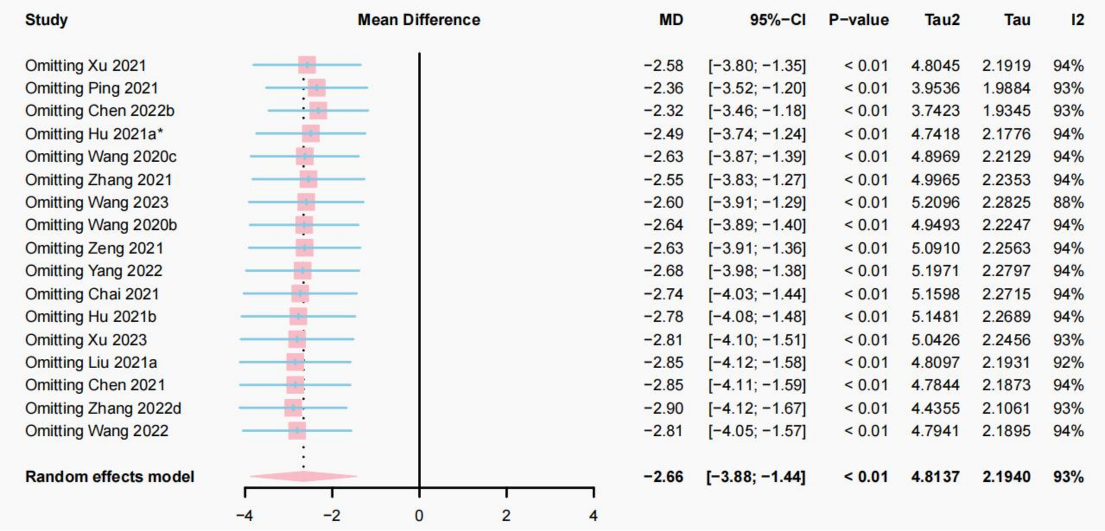

Figure S11. Forest plot of trim-and-fill analysis for clinical efficacy.

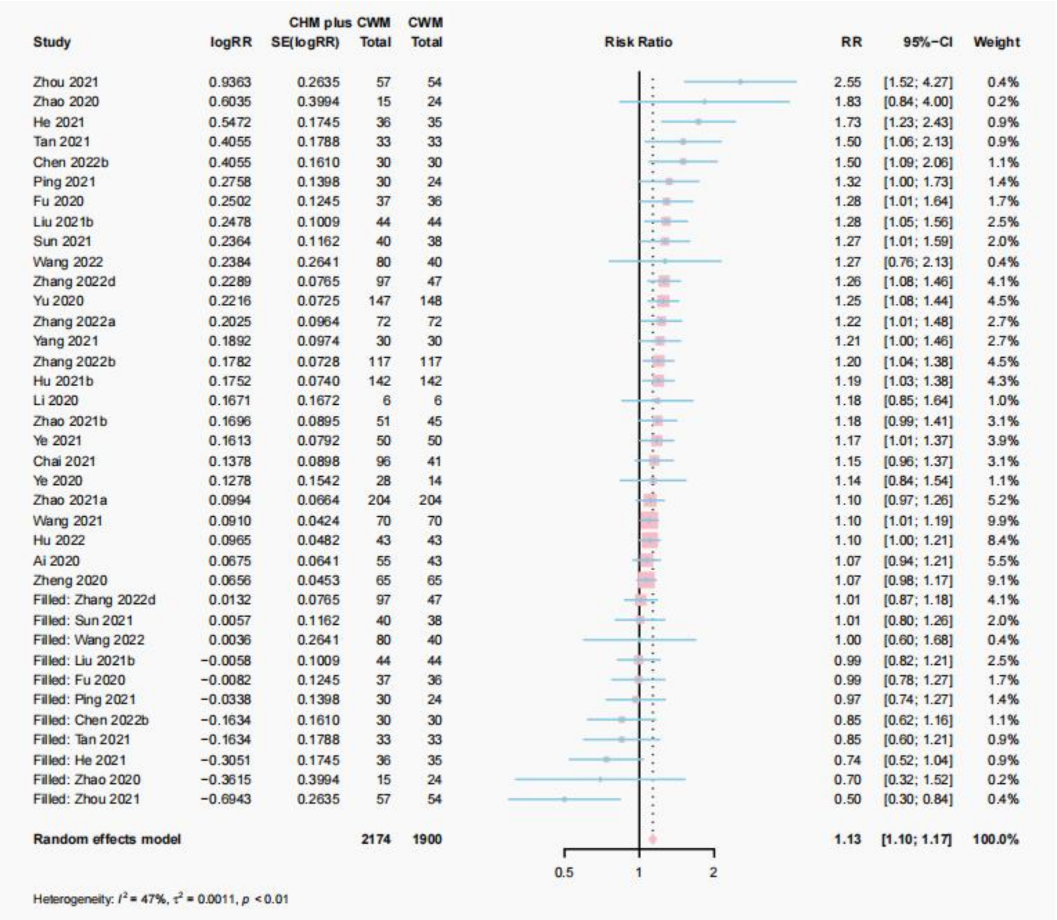

Figure S12. Forest plot of trim-and-fill analysis for conversion to severe cases.

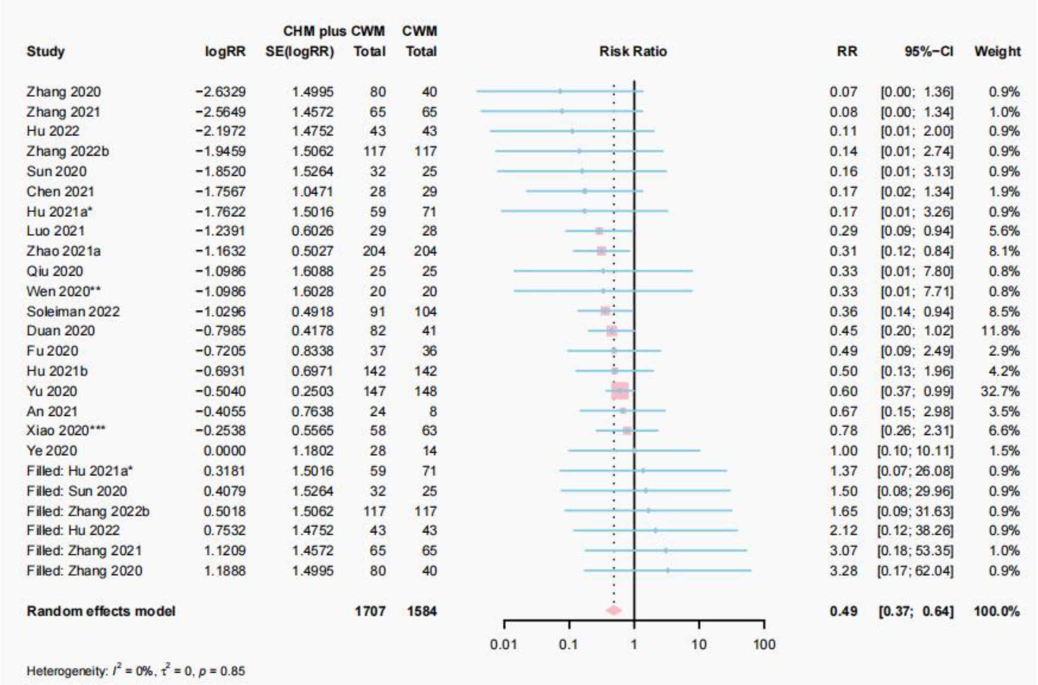

Figure S13. Six key compounds contained in LHQW.

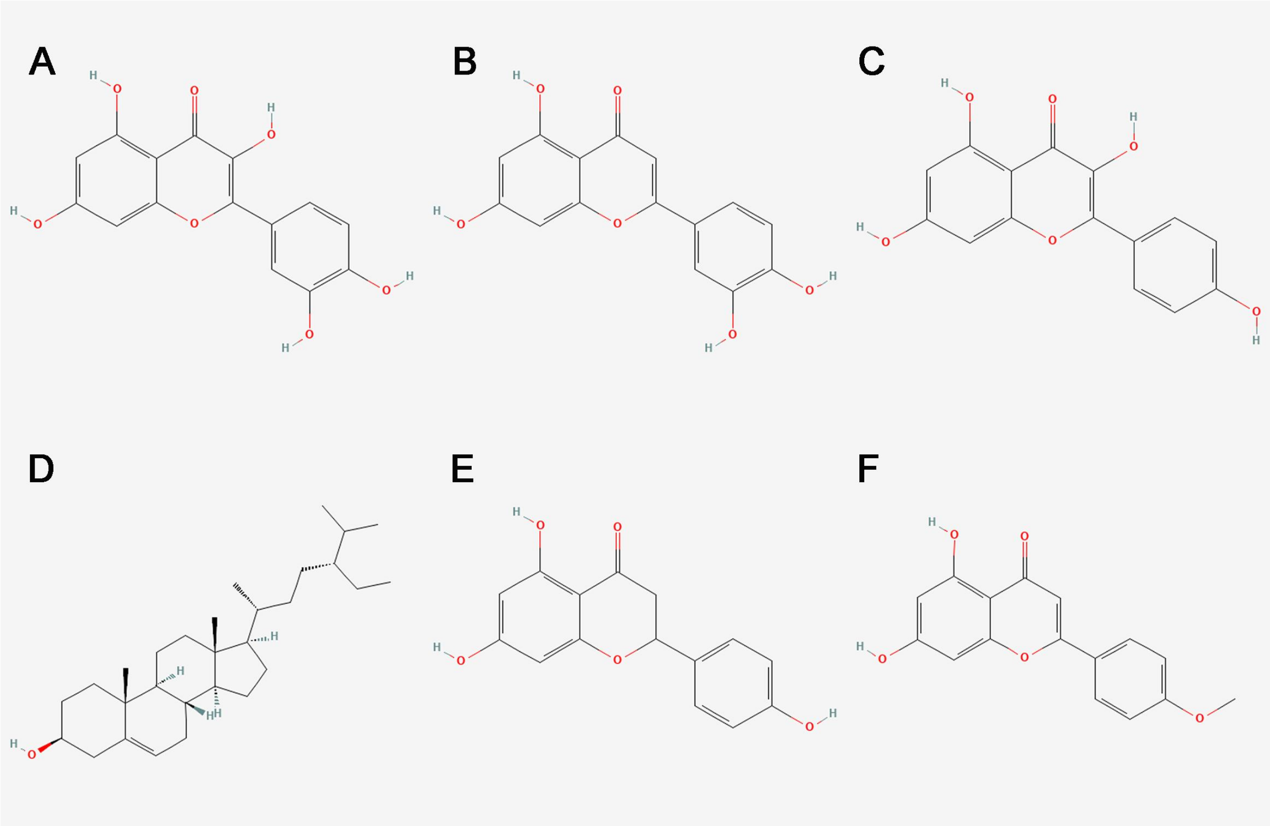

(A) quercetin, (B) luteolin, (C) kaempferol, (D) sitosterin, (E) naringenin, (F) acacetin. Chemical structures quoted from https://pubchem.ncbi.nlm.nih.gov/.

Figure S14. The main active ingredients of several commonly used Chinese herbs.

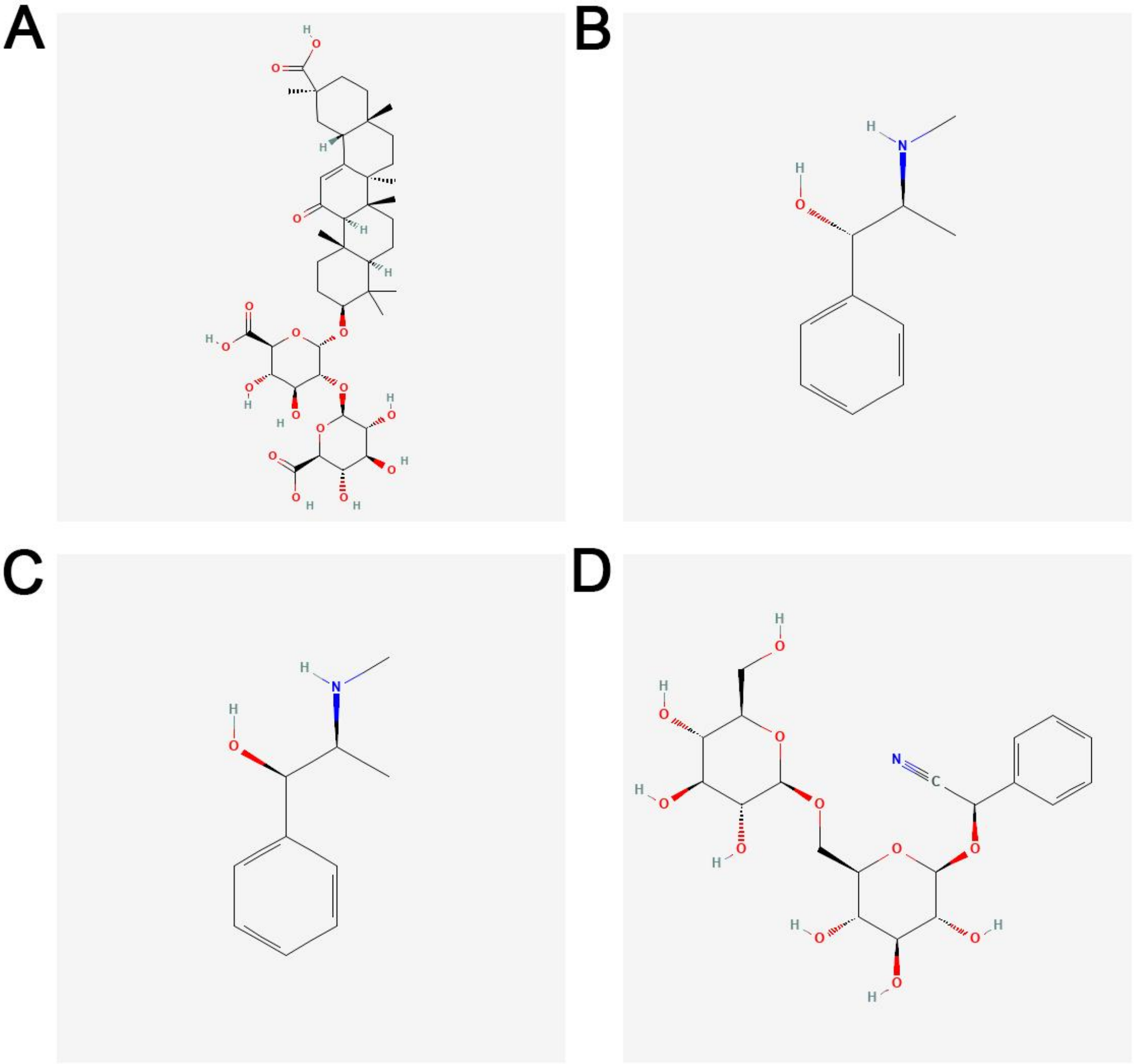

(A) glycyrrhetinic acid, (B) pseudoephedrine, (C) ephedrine, (D) amygdalin. Chemical structures quoted from https://pubchem.ncbi.nlm.nih.gov/.
